# Reinventing the Corey–Fuchs Reaction under Ball Milling Conditions: A Mechanochemical Pathway to Bromoalkynes

**DOI:** 10.1021/acsomega.6c06178

**Published:** 2026-07-24

**Authors:** Barbara Kaczmarek, Ireneusz Kownacki, Dawid Frąckowiak

**Affiliations:** † Faculty of Chemistry, 49562 Adam Mickiewicz University, Poznań 61-614, Poland; ‡ Center for Advanced Technologies, Adam Mickiewicz University, Poznań 61-614, Poland

## Abstract

The classical Corey–Fuchs reaction, a two-step transformation of aldehydes into alkyne derivatives, is successfully adapted to mechanochemical conditions. The solvent-free process involves two sequential ball-milling steps: (1) the formation of *gem*-dibromoalkenes from aldehydes using only half the conventional amount of triphenylphosphine, followed by (2) a base-promoted dehydrohalogenation that unexpectedly affords bromoalkynes instead of terminal alkynes. Although developing a one-pot protocol proved unfeasible, the stepwise approach provides excellent conversions, short reaction times, and significantly improved sustainability metrics (PMI, E-factor). The study shows that substrate electronics and milling parameters govern reaction efficiency and selectivity, expanding the mechanochemical toolbox for greener, phosphorus-mediated synthesis.

## Graphical abstract

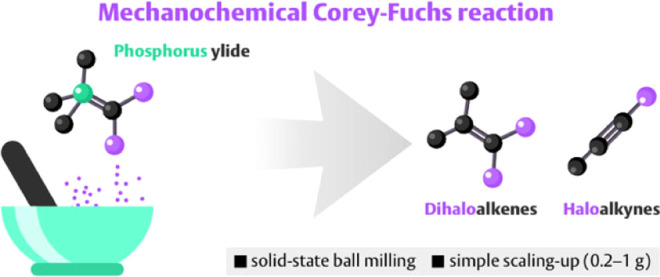

## 1 Introduction

Organophosphorus reagents are essential in organic synthesis due to their versatile reactivity and their capacity to introduce various functionalities into organic molecules.^1^ These reagents, including phosphines, phosphine oxides, and phosphoramidites, are used in a broad range of synthetic applications, both stoichiometric and catalytic.^2^ Among these, phosphorus ylide-based transformations are particularly significant,^3^ such as the Wittig,^4^ Horner–Wadsworth–Emmons,^5^ and Corey–Fuchs^6^ reactions. The Corey–Fuchs process stands out for enabling the simple conversion of readily available aldehydes into 1,1-dibromoalkenes (*gem*-dibromoalkenes) and alkynes.

The first historical synthesis of β,β-dibromostyrene from benzaldehyde using the CBr_4_/PPh_3_ system was described by F. Ramirez and co-workers in 1962 (Scheme 1A).^7^ In this process, the triphenylphosphine-tetrabromomethane system enabled the transformation of an aldehyde into a *gem*-dibromoalkene via a phosphorus *gem*-dihaloylide generated in situ. A decade later, E. J. Corey and A. Fuchs enhanced the Ramirez protocol (Scheme 1B).^6^ Zinc dust was introduced into the reaction mixture to reduce the initially formed Ph_3_PBr_2_ to ZnBr_2_ and PPh_3_. This modification is advantageous in many cases, as the isolation procedure becomes simpler and the yields of dibromoolefins increase. Moreover, CCl_4_ and Cl_4_ can also serve as alternative halogen sources. Corey and Fuchs also added a further step for obtaining the alkyne through the dehydrobromination of the *gem*-dibromoalkene (Fritsch–Buttenberg–Wiechell rearrangement) in the presence of a strong base, e.g., ^n^BuLi,^8^ NEt_3_,^9^ LDA (lithium diisopropylamide)^10^ or DBU (1,8-diazabicycloundec-7-ene),^11^ followed by hydrolysis (Scheme 1C).

**Scheme 1:**
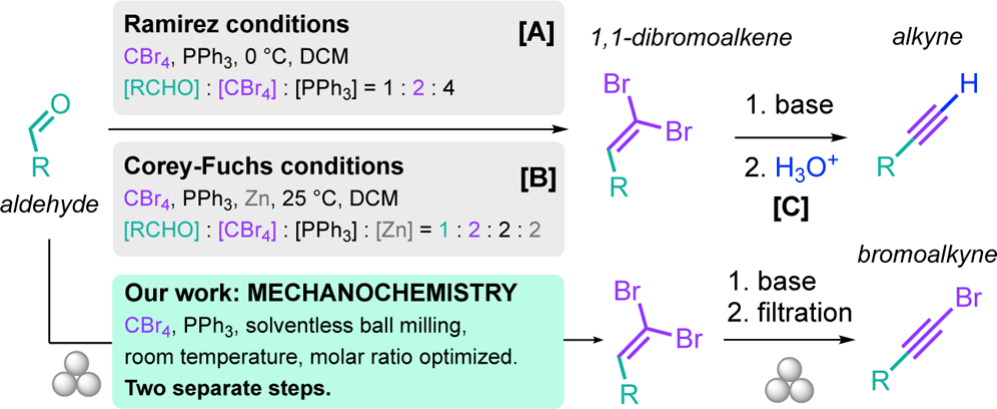
Classic Alkyne Synthesis via *gem*-Dibromoalkenes vs Ball Milling

The mechanism for both steps of the Corey–Fuchs reaction is presented in Scheme 2. Tetrabromomethane is attacked by triphenylphosphine, yielding tribromomethanide ion [CBr_3_]^−^ **a** and bromotriphenylphosphonium cation **b**, which are in equilibrium with triphenyl­(tribromomethyl)­phosphonium bromide **c**. The former is attacked by another PPh_3_ molecule, and the unstable *gem*-dihaloylide **d** is formed, along with reactive dibromotriphenylphosphorane **e**. Ylide **e** reacts with aldehyde **g**, leading to betaine **h**, which equilibrates to oxaphosphatane **i**, which decomposes to 1,1-dibromoalkene **j** with concomitant release of triphenylphosphine oxide (TPPO) via retro-[2 + 2] cycloaddition. It is worth noting that during the synthesis and/or isolation of product **a** (formed either initially or during the decomposition of **c**), a proton may be abstracted from water or another protic solvent, forming small amounts of bromoform **f** as a byproduct. In the second step of the CF reaction, base attacks the alkene proton, resulting in the formation of 1-bromoalkyne **k**. If an organolithium reagent is used as a base (e.g., ^n^BuLi), an alkyne **l** is formed.

**Scheme 2:**
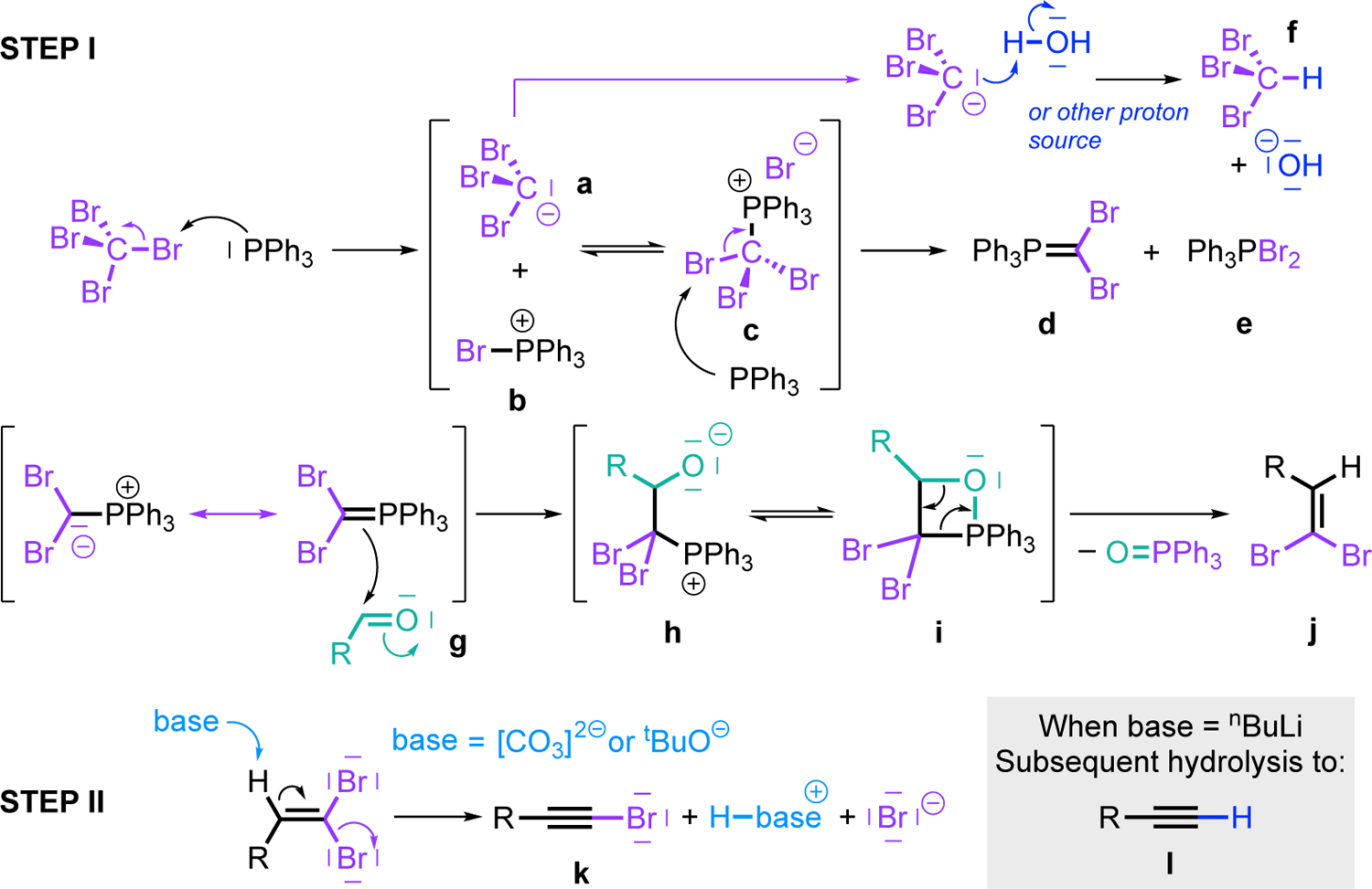
Mechanism for the Corey–Fuchs Reaction

*Gem*-dibromoalkenes are potent building blocks in organic chemistry, so the CF reaction is utilized in the synthesis of several natural products,^12^ such as (±)-roseophilin,^13^ 4,4a-didehydrohimandravine,^14^ (+)-taylorione or (±)-hirsutene.^15^ They are also involved in the synthesis of haloalkynes, heterocycles, carboxylic acids, esters, and thioamides.^16^ The CF protocol can also effectively convert ketones into *gem*-dibromoalkenes; however, extended heating is needed.^17^ Various modifications of this reaction are also known, including replacing PPh_3_ with alkyl phosphites such as P­(O^i^Pr)_3_ or P­(OMe)_3_.^10,11^ Phosphorus *gem*-dihaloylides, e.g., Br_2_C = PPh_3_, are too reactive to be isolated. They can also be generated in situ from the corresponding stable phosphonium salts, e.g., [Br_2_CHPPh_3_]^+^Br^–^.^18^

Unfortunately, the CF reaction has significant drawbacks, including the need for large volumes of solvent during both the reaction and subsequent purification. In some cases, pyrophoric reagents (e.g., ^n^BuLi) or high or low temperatures are required for the dehydrohalogenation of the resulting gem-dibromoalkene. Scaling these reactions or adapting them to one-pot conditions is also challenging. These problems run counter to current trends toward sustainable, environmentally friendly chemical transformations, particularly the 12 principles of green chemistry.^19^ Given the wide availability of PPh_3_, CBr_4_, and aldehydes, we employed the standard reagent system in the first step of the Corey–Fuchs (CF) reaction.^20^ In the second step, alternative bases such as Cs_2_CO_3_ were investigated under mechanochemical conditions. This approach aimed to develop a practical, solvent-reduced synthetic protocol.^11a,21^ .

In our view, many of these drawbacks can be resolved using mechanochemistry. Mechanochemistry investigates the changes in chemical and physicochemical properties induced by applying mechanical energy, such as grinding, crushing, shearing, or cavitation.^22^ Solid-state reactions can be carried out without solvents or with only minute amounts of solvent (LAG; Liquid-Assisted Grinding). One can use a mortar and pestle; however, specialized equipment, such as ball mills, is significantly more effective.^23^ Typically, two main types of ball mills are used for mechanochemical reactions: mixer mills and planetary mills. Such devices are equipped with appropriate vessels and grinding balls made of various materials (including stainless steel, zirconium oxide, and tungsten carbide), allowing for effective reactions.^24^ Currently, the utilization of phosphorus ylides in mechanochemical synthesis is primarily limited to the Wittig and the Horner–Wadsworth–Emmons reactions.^24a,24b^ In general, the field of mechanochemistry in forming P–C bonds (excluding studies on solid-state Kabachnik–Fields reaction)^24c^ and P–X bonds remains relatively underexplored. While Marret et al. obtained four *gem*-dibroalkenes via solid-state ball milling, it was not a classical CF procedure, but a reaction of a hexameric halogen-bonded phosphonium salt cage with aldehydes and ketones in the presence of K_2_CO_3_.^25^ Notably, there are no reports on the mechanochemical Corey–Fuchs reaction. Hence, our paper aims to address this gap by exploring this issue in detail.

## 2 Results and Discussion

When developing a model system to study the CF reaction in a solid state, we initially made several assumptions regarding the conditions, including that all reactants should be solids and the reaction will be carried out at room temperature, with the molar ratio of the reactants [aldehyde]: [PPh_3_]: [CBr_4_] = 1:4:2. We chose 4-nitrobenzaldehyde **1a** as a substrate due to its high reactivity at the carbonyl group tuned by the presence of an electron-withdrawing nitro substituent (Scheme 3).

**Scheme 3:**
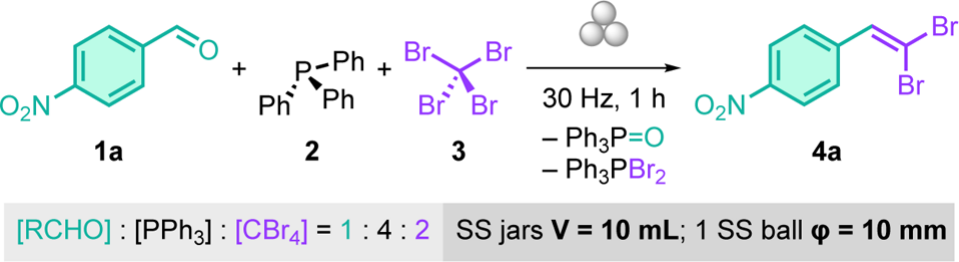
Synthesis of **4a**—Model CF Reaction in the Solid State

All tests were conducted on a 0.05 g scale of **1a** in stainless-steel grinding jars (SS; *V* = 10 mL) with one grinding ball of the same material (φ = 10 mm) using a Retsch MM400 mixer mill. Each reaction was performed simultaneously in two grinding jars to confirm process reproducibility. In the first test, the reagents were ball-milled for 1 h at 30 Hz. After milling, the powders were removed from the jars using polypropylene SmartSpatulas. These spatulas allow effortless material extraction without damaging the steel surfaces of the grinding vessels. Their use is optional but significantly facilitates product isolation and extends the lifespan of the vessels. The postreaction mixture was then analyzed by GC–MS.

The analysis showed complete conversion of the starting aldehyde to the expected dibromoalkene **4a**, along with the formation of triphenylphosphine oxide (TPPO) and a small amount of bromoform (HCBr_3_; Figure 1). The presence of HCBr_3_ results from protonation of the tribromomethanide ion, [CBr_3_]^−^, which is generated in situ by nucleophilic attack of PPh_3_ on CBr_4_ (as previously shown in Scheme 2).

**Figure 1:**
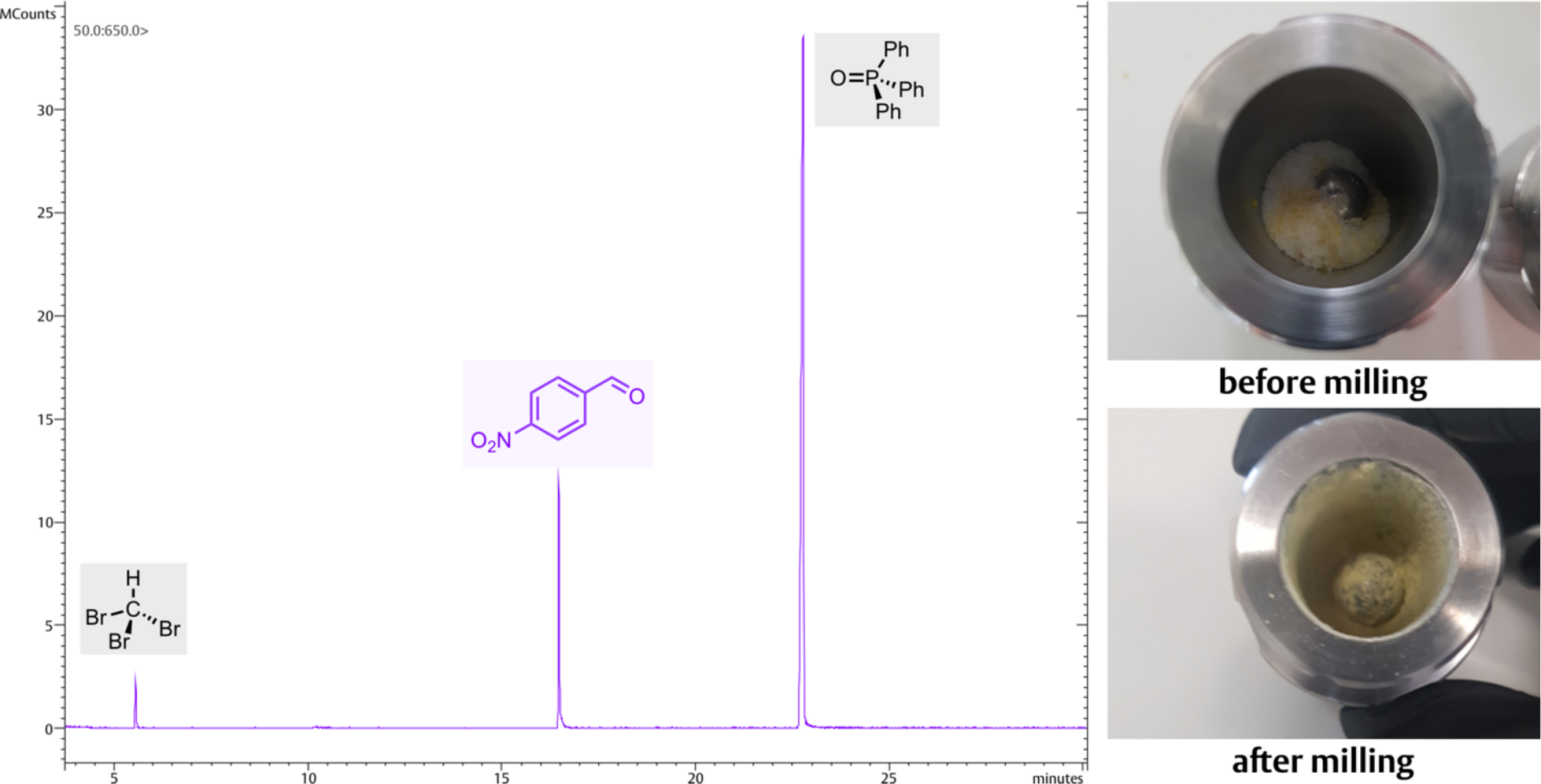
GC–MS analysis of the model reaction.

This phenomenon is usually suppressed by using low temperatures (0 °C) in solution-based protocols, and we also observed it during the synthesis of model compounds.

The next step was to optimize the conditions for the first stage of the CF reaction: oscillation frequency, duration, and substrate molar ratio. Reactions were carried out with 0.05 g of **1a** using 10 mL SS jars with one grinding ball of the same material (φ = 10 mm), and the crude postreaction mixtures were analyzed by GC–MS. The test parameters and results are presented in Table 1. While maintaining the initial frequency and time range (*f* = 30 Hz, *t* = 1 h), it was investigated how the molar ratio of substrates could be reduced compared to the original (i.e., [aldehyde]: [PPh_3_]: [CBr_4_] = 1:4:2). Subsequent studies revealed that complete conversion could be attained with a [aldehyde]: [PPh_3_]: [CBr_4_] ratio of 1:2:2, effectively halving the molar ratio of PPh_3_.

**Table 1:** Optimization of the Model Reaction

no	molar ratio			f [Hz]	t [min]	conv [%]^a^
	RCHO	PPh_3_	CBr_4_			
**1**	1	4	2	30	60	100
**2**	1	4	2	15	60	100
**3**	1	4	2	10	60	85
**4**	1	4	2	5	60	21
**5**	1	4	2	30	30	100
**6**	1	4	2	30	15	100
**7**	1	4	2	30	10	100
**8**	1	4	2	30	5	100
**9**	1	4	2	15	30	91
**10**	1	2	1	30	60	18
**11**	1	2.5	1	30	60	45
**12**	1	3	1	30	60	92
**13**	1	3	2	30	60	100
**14**	1	2	2	30	60	100
**15**	1	2	2	30	5	100

a Approximate conversion value monitored by GC–MS analyses.

Then, a series of tests was conducted under conditions analogous to the initial tests, with only the frequency varied. Complete conversion of **1a** to **4a** was achieved at both f = 30 Hz and f = 15 Hz (Table 1). Subsequent tests examined how milling time affected process efficiency. Therefore, runs were conducted with gradually shortened reaction times. At *f* = 30 Hz, complete conversion of **1a** to **4a** occurred within 5 min, whereas at *f* = 15 Hz, the tests produced a mixture of **1a** and **4a**.^31^

Since our goal was not only to simplify the execution of the CF reaction but also to create a more “green” variant of this process, we aimed to avoid column chromatography, the most commonly used purification technique for this reaction. According to the literature, significant amounts of TPPO can be removed by forming complexes with salts such as ZnCl_2_, which are poorly soluble in organic solvents.^26^ First, we tested whether the zinc complex could be obtained by ball milling. For the complexation reaction, we conducted tests in SS vessels (V = 10 mL) with one SS grinding ball (φ = 10 mm), using 0.4 g TPPO at a molar ratio of [TPPO]: [ZnCl_2_] = 2:1 (Scheme 4).

**Scheme 4:**
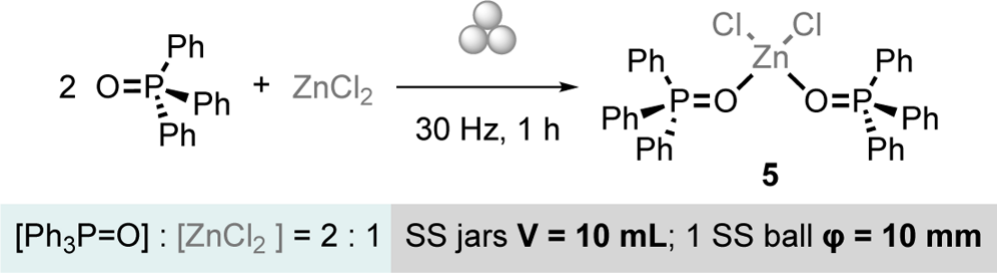
Synthesis of [ZnCl_2_(TPPO)_2_] **5** Using Ball Milling

The formation of **5** proceeded smoothly, as confirmed by ^31^P NMR analysis. The ^31^P NMR spectrum of this complex has not been reported in the literature, but the expected chemical shift toward a higher ppm value (δ = 38.15 ppm) was observed,^27^ and the TPPO signal (δ = 29.3 ppm)^28^ disappeared.

The first purification variant involved adding a stoichiometric amount of ZnCl_2_ directly to the vessel after formation of **4a**, followed by milling and precipitation in EtOH. Unfortunately, the products still contained TPPO impurities. Next, a different procedure was tested by dissolving the postreaction mixture in EtOH and then adding ZnCl_2_; however, the result was the same. It was decided that column chromatography would remain the purification method; the pure product **4a** was isolated, and its spectroscopic characteristics matched those reported in the literature.

To confirm that the model CF reaction proceeds in the solid state, the postreaction mixtures were analyzed by solvent-free ATR-FTIR and PXRD. Figure 2 shows the FT-IR spectra of 4-nitrobenzaldehyde **1a** and the mixtures obtained after grinding with CBr_4_ and PPh_3_, revealing the disappearance of the C=O band at 1701 cm^–1^, and appearance of the P=O band (1182 cm^–1^),^29^ indicating the formation of TPPO. Additionally, spectra of 1,1-dibromoalkene **4a**, obtained by traditional solution-phase synthesis and by grinding, were compared and found to be identical (in line with the literature).^11b^ The results of PXRD analyses are shown in Figure 3. The phase of the postreaction mixtures (Figure 3C) is partially crystalline, but there is also an amorphous part. No reflections from the aldehyde **1a** (Figure 3B) are observed (8.3°, 16.7°, 17.2°, 27.4°, for the pure compound), indicating the complete conversion to **4a**. The phase of the pure product mainly consists of three reflections at approximately 10.8°, 21.6°, and 32.6° (Figure 3A), which are identifiable in PXRD of the postreaction mixture (11.1°, 21.8°, 32.8°).^33^ The other identifiable component is TPPO. The remaining reflections most likely originate from other byproducts present in the system.^34^ Additionally, PXRD analysis of the postreaction mixture was performed to identify CBr_4_ (see Figure S3 in ESI for details).^35^

**Figure 2:**
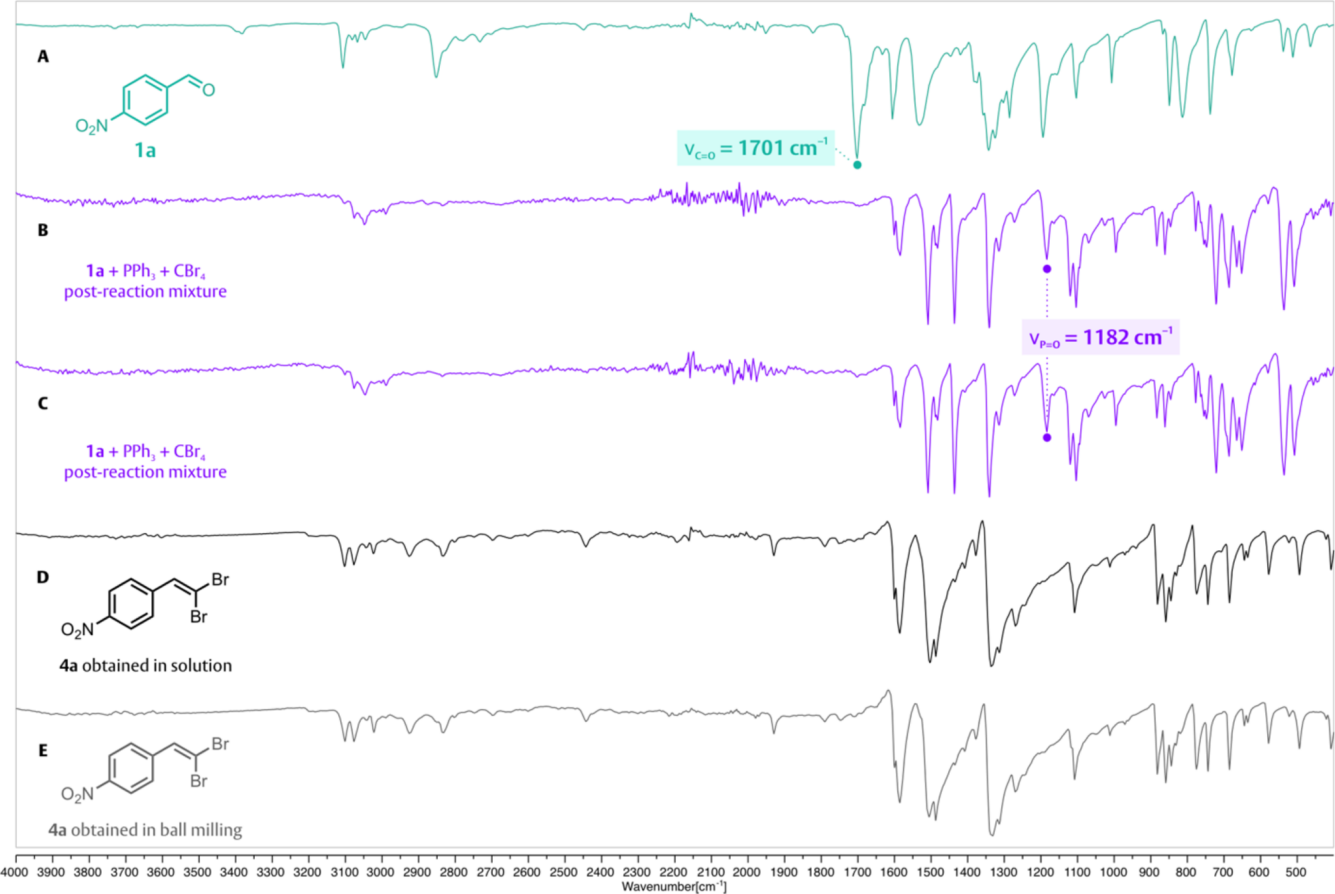
Comparison of ATR FT-IR spectra of: (A) **1a**; (B–C) postreaction mixtures of **1a** with CBr_4_ and PPh_3_;^36^ (D) isolated **4a** obtained in solution; (E) isolated **4a** obtained in ball milling. Bands originating from the carbonyl group of **1a** were identified—ν_(C=O)_ = 1701 cm^–1^—as well as vibrational bands of the *P* = O group of TPPO: ν_(*P*=O)_ = 1182 cm^–1^. The band observed in the aldehyde spectrum appears to be identical to the TPPO band; however, it is located at 1195 cm^–1^.

**Figure 3:**
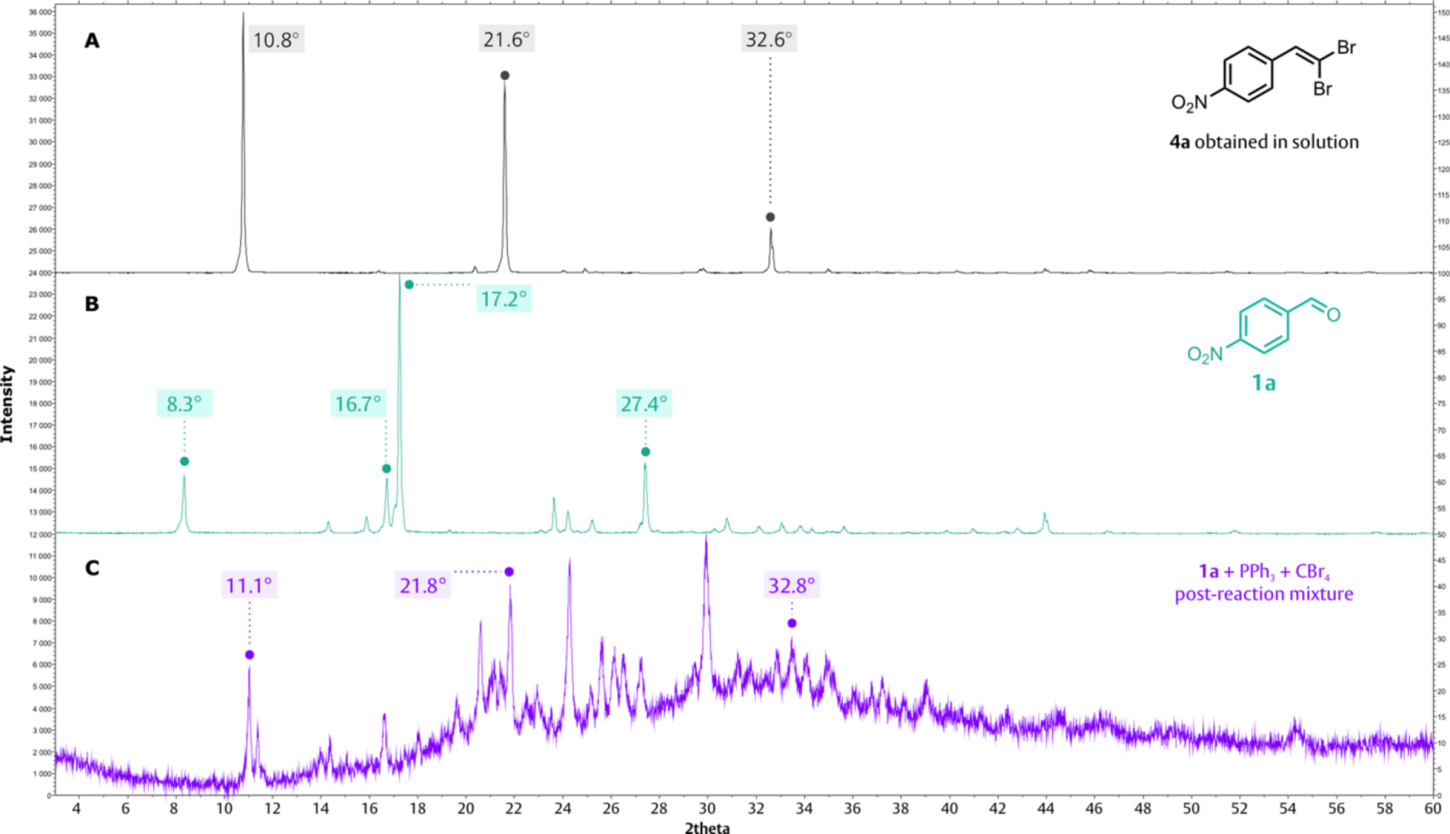
Comparison of PXRD diffractograms of: (A) pure **4a** obtained in solution; (B) 4-nitrobenzaldehyde **1a**; (C) postreaction mixture of **1a** with CBr_4_ and PPh_3_.

A more detailed PXRD analysis of the organophosphorus components of the system is presented in Figure S2 in the ESI. A variety of organophosphorus compounds are observed that may serve as intermediates or side products of the reaction. In general, reflections attributable to Ph_3_PBr_2_, Ph_3_PCBr_5_, and—most likely—the salt shown in the reaction mechanism, triphenyl­(tribromomethyl)­phosphonium bromide (Scheme 2, **c**), are observed. However, due to the complex reflection patterns, there is no certainty in assigning their identities. Ball milling equimolar amounts of PPh_3_ and CBr_4_ produced either amorphous (room temperature) or mostly amorphous phases (milling at low temperature—ca. 0 °C); apparently, only the presence of PPh_3_ is evident from the resulting PXRD analyses (see Figure S4 in the ESI).

After optimizing the reaction parameters, experiments were carried out with various aldehydes on a 0.2 g scale in 10 mL stainless-steel milling jars, each containing a 10 mm-diameter grinding ball made of the same material (Scheme 5). The reaction was followed by column chromatography. All isolated compounds (and those not isolated or obtained) are collected in Table 2. Solid-state ball milling of benzaldehyde **1n** led to a mixture of (2,2-dibromovinyl)­benzene **4n** (70%, approximate value determined by GC–MS analysis) and benzylidene dibromide (30%). The formation of 1,1-dibromoalkane is not unexpected, as such reactivity can occur in systems involving R_2_PBr_2_ species.^30^ A similar situation took place in the case of 1,4-bis­(2,2-dibromoethenyl)­benzene, **4g**, but the amount of side product, 1-(dibromomethyl)-4-(2,2-dibromovinyl)­benzene was only approximately 3% (GC–MS). Furthermore, even under optimized conditions, the conversion to **4g** was incomplete. Consequently, the diameter of the milling ball was adjusted from 10 mm to 12 mm to enhance the energy delivered to the reaction mixture, and ultimately, the product could be isolated and purified.

**Scheme 5:**
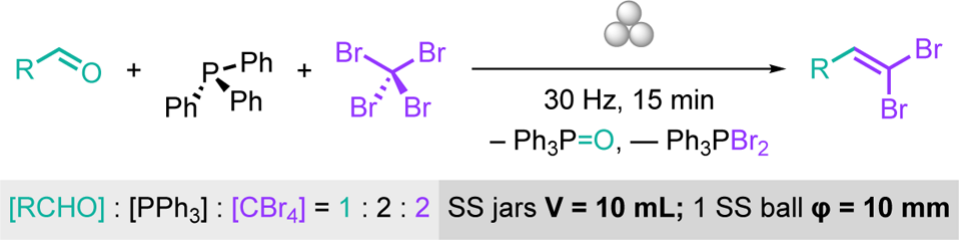
General Protocol for the Mechanochemical Synthesis of 1,1-Dibromoalkenes

**Table 2:**
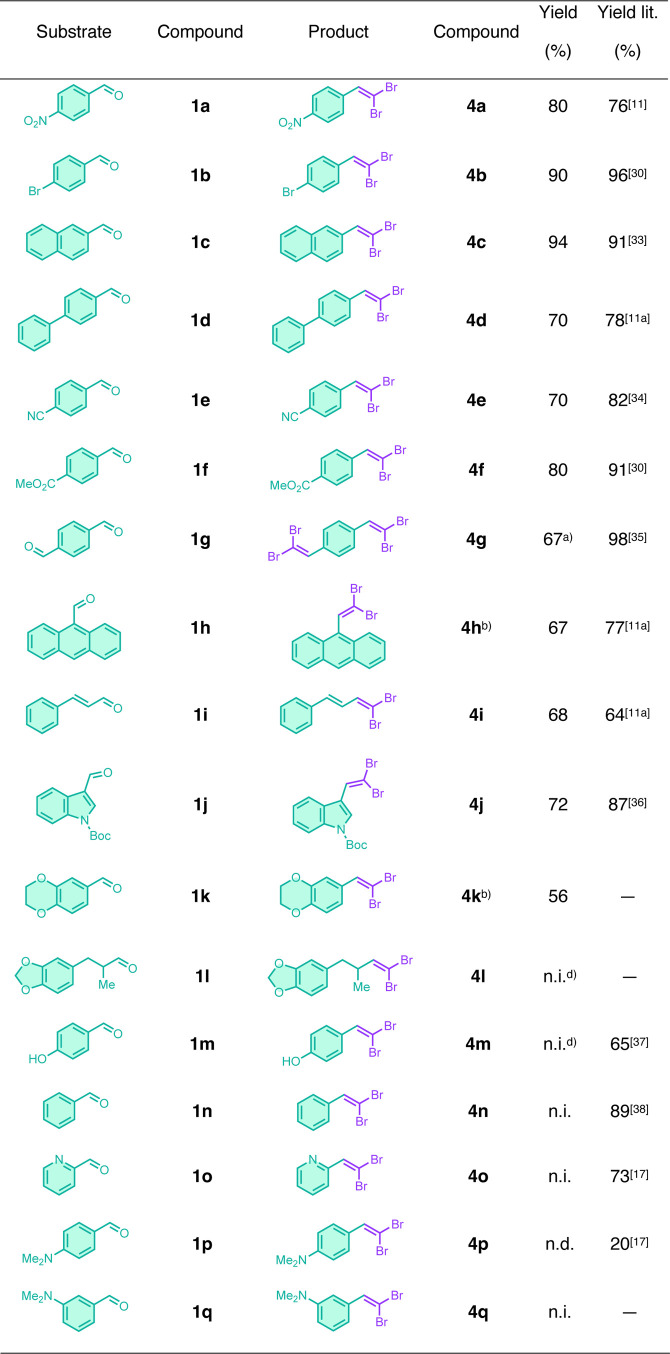
Conversion of Aldehydes Into 1,1-Dibromoalkenes Using a Mechanochemical Protocol^a^

a n.i.—not isolated. n.d.—not detected. (a) Default parameters of mixer mill used: *f* = 30 Hz, *t* = 1 h; (b) traces of aldehyde (<5%); (c) contains ca. 10% of helional; (d) the diameter of the milling ball was changed from 10 to 12 mm.

Problems have also been encountered in the dibromolefination of 4-hydroxybenzaldehyde **1h**. Unfortunately, under optimized conditions, the process did not proceed to completion, and GC–MS analysis still showed the presence of unreacted aldehyde. In that case, synthesis was performed using the molar ratio of the substrates as described in the Ramirez procedure, with ball milling at 30 Hz for 60 min. We did not isolate 4-(2,2-dibromovinyl)­phenol **4m** because it is unstable and decomposes within a few days.^37^ On the other hand, dibromoolefination of 4-dimethylaminobenzaldehyde (DABA) **1p** was unsuccessful. In principle, such an outcome for this reaction was expected due to the substantial resonance contribution of the dimethylamino group in the formation of the zwitterionic quinoid structure (Scheme 6).

**Scheme 6:**
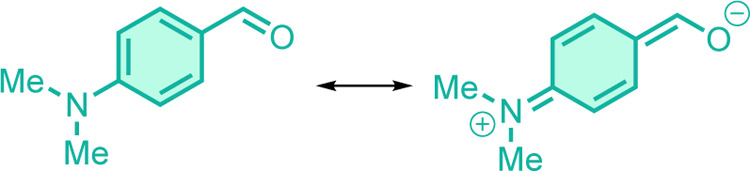
Zwitterionic Quinoid Structure of **1p** (DABA)

However, 4-(2,2-dibromovinyl)-*N*,*N*-dimethylaniline **4p** has been previously prepared in solution, albeit with low yield (e.g., in microwave-assisted synthesis, isolated yield: 20%).^17^ It has been reported that the behavior of DABA in polar solvents may change somewhat (e.g., by forming charge–transfer complexes or protonation).^32^ This may be why dibromoolefination occurs in DCM solution rather than during solid-state ball milling. On the other hand, the reaction with 3-(dimethylamino)­benzaldehyde **1q** proceeded smoothly, affording product **4q** in complete conversion (see Figures S6 and S7 in the ESI).

Scaling tests were also conducted using 1 g of **1a** based on the model reaction. Due to the large volume of the reaction mixture, 50 mL SS vessels with ten grinding balls made of the same material (φ = 10 mm) were used for this purpose. Initially, studies were conducted in a mixer mill using an optimized procedure; however, GC–MS analysis revealed traces of aldehydes. Therefore, the milling time was extended to 30 min, resulting in complete conversion to the desired product. The planetary mill offers a significantly larger grinding vessel volume (up to 500 mL), thereby enabling further scaling up. For this reason, a similar reaction was conducted in a Retsch PM100 planetary mill using a suitable 50 mL SS vessel with ten grinding balls made of the same material (φ = 10 mm). Unfortunately, only 90% conversion of **1a** to **4a** was achieved despite changing milling parameters. The aldehyde remained in the postreaction mixture. The green metrics, PMI and E-factor, were also calculated based on the model reaction using 1 g of **1a**. The difference in values between the solvent-based synthesis method and the mechanochemical variant is significant. In the solvent-based synthesis, the PMI is 468.4, and the E-factor is 467.4; in the mechanochemical variant, the values are 241.8 and 240.8, respectively. These values, which are about half as small for the mechanochemical method, clearly indicate that this is a greener and more sustainable approach. The mechanochemical method consumes fewer raw materials and generates less waste, making it more environmentally friendly.

Subsequently, the CF reaction was examined in a one-pot variant. All tests were conducted on a 0.05 g scale of **1a**, under identical conditions to those used for the first step of the reaction: PPh_3_, CBr_4,_ and one of the bases (K_2_CO_3_, KOH, Cs_2_CO_3_, or ^t^BuOK). After milling the aldehyde with PPh_3_ and CBr_4_ for 1 h at 30 Hz, the appropriate base was added to the jar, and milling was repeated under the same conditions. These reactions were also conducted using a stoichiometric amount of H_2_O as a proton donor (Scheme 7), obtaining the same outcome.

**Scheme 7:**
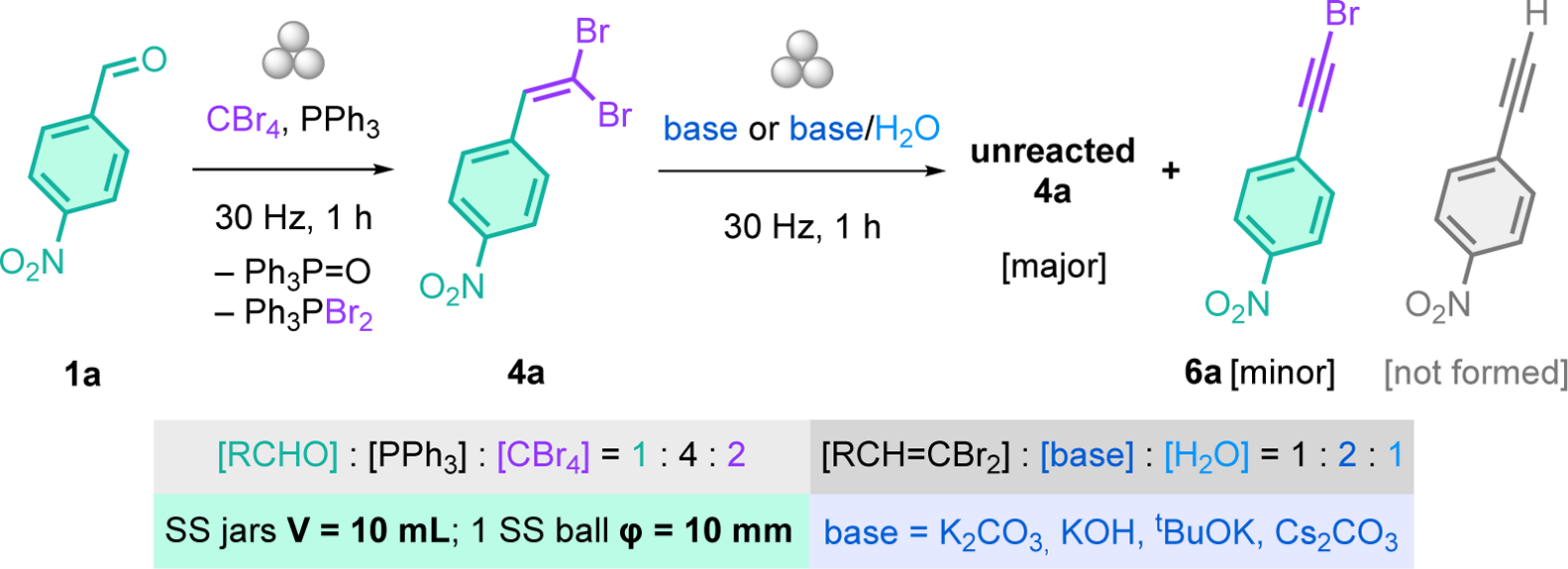
Attempted One-Pot Mechanochemical Corey–Fuchs Reaction

In none of the cases was the terminal alkyne formed; instead, the major component of the postreaction mixtures was unreacted **4a**, along with minor quantities of 1-bromoalkyne **6a** (see Figure S5 in the ESI). This behavior indicates that only the first step can be performed under mechanochemical conditions in this configuration. The reaction byproducts, including Ph_3_PBr_2_, react with the basic reagents used, as confirmed by milling pure **4a** with Ph_3_PBr_2_, yielding the same result shown in Scheme 7. All reaction details are collected in Figure S8 in the ESI.

When the one-pot reaction could not be performed, we decided to carry out the second step of the Corey–Fuchs reaction separately, using **4a** obtained in the first step. All tests were conducted on a 0.1 g scale of **4a** in stainless steel grinding jars (*V* = 5 mL) and two grinding balls made of the same material (φ = 7 mm) using a Retsch MM400 mixer mill. We quickly discovered that during initial tests with selected inorganic bases, such as Cs_2_CO_3_, K_3_PO_4_, and CsOH · H_2_O, the **4a** disappeared completely from the reaction mixture, accompanied by the appearance of a new signal corresponding to a new product. However, the product obtained was not the expected terminal alkyne, but rather the 1-bromoalkyne **6a** (Scheme 8). However, this result is consistent with the literature, which reports that 1-bromoalkynes are often obtained via base-promoted elimination of *gem*-dibromoalkenes (e.g., with DBU).^39–42^ This single E2-type dehydrohalogenation is fast and accessible: the base abstracts a vicinal proton from the *gem*-dibromoalkene, and the concomitant elimination of Br^–^ affords **6a**.

**Scheme 8:**
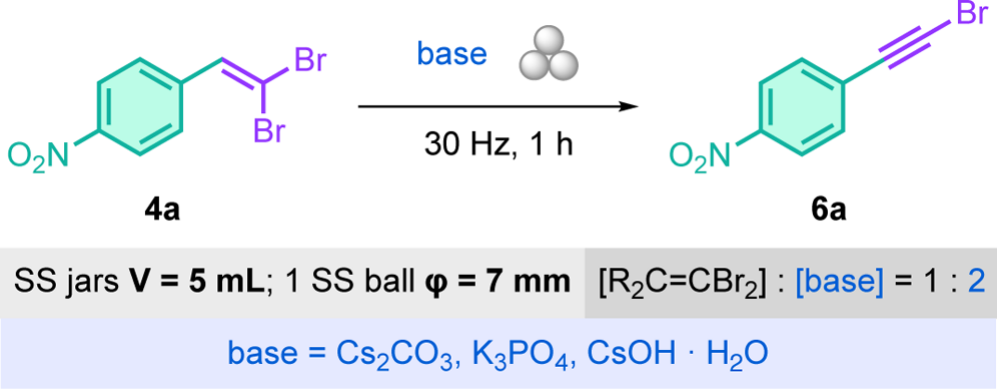
Solid-State Elimination of *gem*-Dibromoalkene **4a** to 1-Bromoalkyne **6a**—Model Reaction

To investigate whether alternative conditions could enable the formation of the terminal alkyne under mechanochemical conditions, we examined a range of inorganic and organic bases in combination with **4a** (Table 3). We also studied the effect of a small amount of water or DMSO on the reaction mixture, as well as the efficiency of other systems reported in the literature. Increasing base strength [using DMAP (4-dimethylaminopyridine) or DBU] likewise failed to promote the desired transformation.

**Table 3:** Optimization of the Model Second-step Reaction Protocol

no	base/system	molar ratio4a: X: Y	f [Hz]	t [min]	conv to 6a [%]
**1**	K_2_CO_3_	1:2	30	60	0
**2**	KOH	1:2	30	60	0
**3**	NaOH	1:2	30	60	0
**4**	Cs_2_CO_3_	1:2	30	15	100
**5**	K_3_PO_4_	1:2	30	60	100
**6**	CsOH · H_2_O	1:2	15	60	100
**7**	[Me_4_N]OH · H_2_O	1:2	15	60	100
**8**	Cs_2_CO_3_/H_2_O (LAG)	1:2	30	15	100
**9**	Al_2_O_3_ (basic)	1:2	30	30	0
**10**	DMAP	1:2	30	60	0
**11**	^t^BuOK	1:2	30	30	100
**12**	K_2_CO_3_/succinimide/DMSO^42^ (LAG)	1:6: 2	30	60	100
**13**	DBU/H_2_O (LAG)	1:2	30	60	0

In all cases, the only product obtained was **6a**. Tests were also performed for **4a** (using Cs_2_CO_3_) and **4c** (using ^t^BuOK) at different time intervals (5, 15, and 30 min) at f = 30 Hz, using various numbers of stainless steel balls (10 × 5 mm and 2 × 10 mm). In general, the elimination proceeded with full conversion of the corresponding 1,1-dibromoalkene to 1-bromoalkyne after 5 or 15 min; however, these results were not always reproducible.

Therefore, we adopted the milling conditions shown in Scheme 8 as the standard protocol. The reaction was then applied to other dibromoalkenes under identical milling conditions, with a molar ratio of [dibromoalkene]: [base] = 1:2 (Scheme 9, Table 4). For several examples listed in the table, Cs_2_CO_3_ did not afford complete conversion to the corresponding bromoalkyne. Consequently, ^t^BuOK was employed to synthesize **6c**, **6d**, **6g**, and **6h**. For **6c**, **6d**, and **6h**, complete conversion was achieved by increasing the dibromoalkene-to-base molar ratio to 1:3. For the planetary ball mill, the reaction was performed on a 1 g scale of **4a** using Cs_2_CO_3_ as the base in 50 mL vessels with seven milling balls at 30 Hz. After 1 h, GC–MS analysis showed modest conversion to **6a** (∼50%), which increased to 98% after 2 h and reached completion after 2.5 h.

**Scheme 9:**
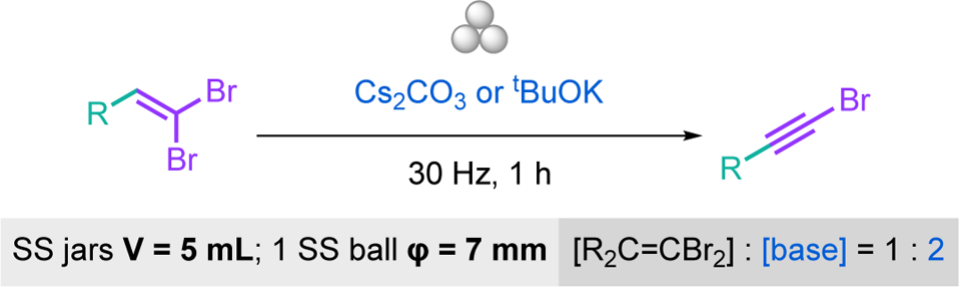
General Protocol for the Mechanochemical Synthesis of 1-Bromoalkynes

**Table 4:**
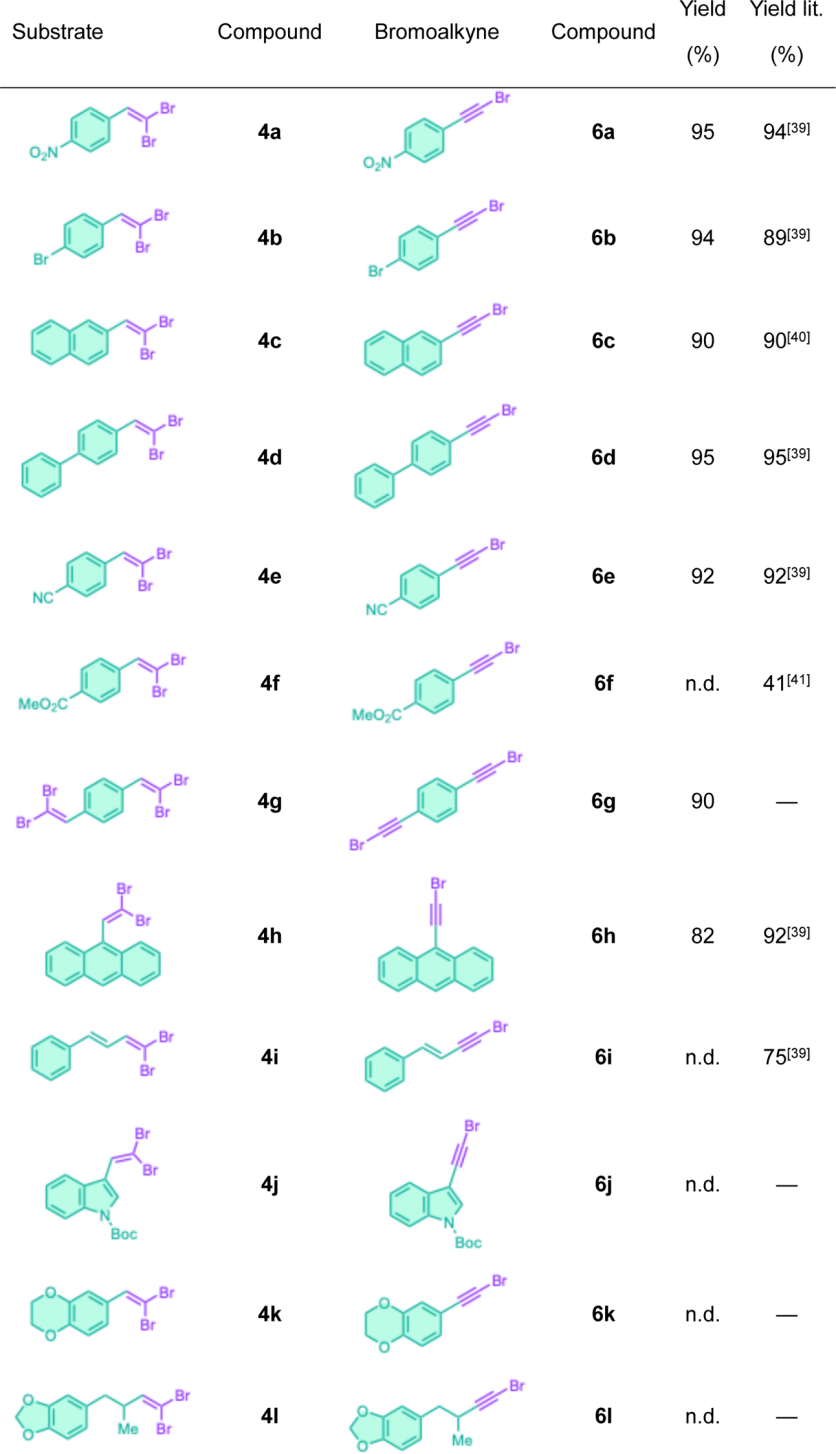
Conversion of *gem*-Dibromoalkenes Into Bromoalkynes Using a Mechanochemical Protocol^a^

a n.i.—not isolated. n.d.—not detected. [RCH = CBr_2_]: [base] = 1:1. For **6a–b** and **6e–f**, Cs_2_CO_3_ was used as a base. For **6c**–**d** and **6g**–**k**, ^t^BuOK was employed as a base. For **6c**–**d** and **6h**, complete conversion was achieved by increasing the dibromoalkene-to-base molar ratio to 1:3. (a) Default parameters of mixer mill were used: *f* = 30 Hz, *t* = 60 min.

Based on these results, ^t^BuOK was selected for scale-up in the planetary mill (Retsch PM100). However, after 1 h of milling at 400 rpm, the conversion was minimal (∼15%). Repeating the experiment with twice the amount of base afforded pure **6a** after 2 h. These findings indicate that while Cs_2_CO_3_ can provide high conversions with sufficient milling time, ^t^BuOK requires higher loadings and longer reaction times to achieve comparable efficiency under planetary milling conditions.

As in the first step, the PMI and E-factor were also calculated for this reaction and found to be 17.3 and 16.3, respectively. Compared with the solvent-based synthesis of **4a** reported in the literature, for which these metrics were estimated at 26.4 and 25.4, a substantial reduction was achieved. This improvement is mainly due to reduced solvent use.

## 3 Conclusion

In summary, a fast, solvent-free, and milder mechanochemical approach for the synthesis of *gem*-dibromoalkenes and bromoalkynes has been developed. The classical Corey–Fuchs reaction was successfully adapted to solid-state conditions, delivering a two-step, sequential ball-milling protocol for the efficient conversion of aldehydes into reactive alkyne derivatives.^38^ Although a one-pot mechanochemical variant proved unfeasible, the sequential approach provided high conversions and excellent substrate tolerance in most cases, with significantly reduced reaction time and minimal solvent use.

The developed reaction protocol halved the amount of PPh_3_ required in the first step while maintaining high selectivity, thereby improving both process efficiency and environmental metrics.

Although purification still requires chromatography and removal of triphenylphosphine oxide, the overall procedure has been significantly improved. On the other hand, the elimination step proceeded smoothly for a broad range of substrates, though it remained inefficient for compounds bearing acidic or nucleophilic functional groups (e.g., phenols, amines, or esters). Notably, the transformation unexpectedly furnished bromoalkynes, valuable intermediates for downstream synthetic applications, rather than terminal alkynes.

Overall, this work demonstrates the feasibility of performing a classical named reaction entirely under solvent-free, mechanochemical conditions, extending the potential of green mechanosynthesis in organic transformations and highlighting a promising direction for sustainable multistep reaction design.

## 4 Experimental Section

### 4.1 Analytical Methods

^1^H and ^13^C­{^1^H} NMR spectra were recorded on a Bruker Ultrashield 300 MHz operating at 300 and 101 MHz, respectively. ^31^P­{^1^H} NMR spectra were recorded on a Bruker Ascend 400 MHz Nanobay operating at 162 MHz. Mass spectra were recorded using a Gas Chromatograph with Mass Detection (TripleQuad MS-320, equipped with direct analysis methods—DIP and DEP modes). FT-IR analyses were performed on a Nicolet iS 50 FT-IR spectrometer. Powder X-ray Diffraction analyses (PXRD) were recorded on SmartLab diffractometer (Rigaku) with Cu anode, vertical θ/θ goniometer in T–T geometry, minimum step size 0.0001, 2θ angular range 0–130°, and D/tex Ultra 250 detector. Measurement parameters: 2θ angle range 3–60°, step: 0.01°.

### 4.2 Ball Milling Methods

The mechanochemical syntheses and tests used.

- Retsch MM400 mixer mill with stainless-steel jars (V = 5 and 10 mL) and balls (φ = 7 and 10 mm). Reactions were performed in two jars for reproducibility. After milling, the mixtures were extracted, and the jars and balls were rinsed with dichloromethane, washed with detergent and water, rinsed with acetone, wiped dry, and oven-dried at 50 °C for 1 h.
- Retsch PM100 planetary mill with stainless-steel jar (V = 50 mL) and balls (φ = 10 mm).

## Supplementary Material



## Notes

Initial tests of the Corey–Fuchs reaction on a scale of 0.05 g of 4-nitrobenzaldehyde (Scheme S1, Figure S1, Picture S1); PXRD analyses (Figures S2–S4); GC–MS analyses (Figures S5–S8); detailed procedures on the mechanochemical synthesis and isolation of 1,1-dibromoalkenes, 1-bromoalkynes, and [ZnCl_2_(Ph_3_P = O)_2_] (Schemes S2–S4); calculation of green metrics (Tables S1–S2); 1H, ^13^C­{^1^H}, ^31^P­{^1^H} NMR, MS (EI) and FT-IR spectra (Figures S9–S81) (PDF)

## References

[1] Svara, J. ; Weferling, N. ; Hofmann, T. Ullmann’s Encyclopedia of Industrial Chemistry; Wiley VCH: Weinheim, 2006.

[2] Xie C., Smaligo A. J., Song X.-R., Kwon O. (2021). Phosphorus-Based Catalysis. ACS Cent. Sci..

[3] Schmidbaur H. (1983). Phosphorus
Ylides in the Coordination Sphere of Transition Metals: An Inventory. Angew. Chem., Int. Ed. Engl..

[4] Heravi M., Zadsirjan V., Daraie M., Ghanbarian M. (2020). Applications
of Wittig Reaction in the Total Synthesis of Natural Macrolides. ChemSelect.

[5] Roman D., Sauer M., Beemelmanns C. (2021). Applications
of the Horner–Wadsworth–Emmons
Olefination in Modern Natural Product Synthesis. Synthesis.

[6] b Biggs, R. A. ; Ogilvie, W. W. Comprehensive Organic Synthesis II, 2nd ed.; Elsevier, 2014; Vol. 2, p 802.

[7] Desai N. B., McKelvie N., Ramirez F. (1962). A New Synthesis
of 1,1-Dibromoölefins
via Phosphine-Dibromomethylenes. the Reaction of Triphenylphosphine
with Carbon Tetrabromide. J. Am. Chem. Soc..

[8] Tsuboya N., Hamasaki R., Ito M., Mitsuishi M., Miyashita T., Yamamoto Y. (2003). Nonlinear optical properties of novel
fullerene–ferrocene hybrid molecules. J. Mater. Chem..

[9] Pradhan T. K., Lin C. C., Mong K. K. T. (2013). Formal
Synthesis of 3-Deoxy-d-manno-Octulosonic
Acid (KDO) and 3-Deoxy-d-arabino-2-heptulosonic Acid (DAH). Synlett.

[10] Martynow J., Hanselmann R., Duffy E., Bhattacharjee A. (2019). Preparation
of Ethynylbenzene Derivatives from Substituted Benzaldehydes. Org. Process Res. Dev..

[11] Thummala Y., Karunakar G. V., Doddi V. R. (2019). DBU-Mediated Synthesis
of Aryl Acetylenes or 1-Bromoethynylarenes
from Aldehydes. Adv. Synth. Catal..

[12] Chelucci G. (2012). Synthesis
and Metal-Catalyzed Reactions of gem-Dihalovinyl Systems. Chem. Rev..

[13] Bitar A. Y., Frontier A. J. (2009). Formal Synthesis
of (±)-Roseophilin. Org. Lett..

[14] Wong L. S. M., Sharp L. A., Xavier N. M. C., Turner P., Sherburn M. S. (2002). The Bromopentadienyl
Acrylate Approach to Himbacine. Org. Lett..

[15] Oppolzer W., Robyr C. (1994). Synthesis of (±)-hirsutene by a catalytic allylpalladium-alkyne
cyclization/carbonylation cascade. Tetrahedron.

[16] Williams M.
B., Martin M. L., Wiedmann S., Boyer A. S. (2023). Exploiting 1,1-Dibromoalkenes as
Direct Precursors
to 5-Substituted 1,2,3-Triazoles. Synthesis.

[17] Nauroozi D., Bruhn C., Fürmeier S., Holzhauer J.-U., Faust R. (2016). Microwave-Assisted Dibromoolefination of Aromatic and Heteroaromatic
Aldehydes and Ketones. J. Heterocycl. Chem..

[18] Michel P., Gennet D., Rassat A. (1999). A one-pot procedure for the synthesis
of alkynes and bromoalkynes from aldehydes. Tetrahedron Lett..

[19] Anastas P., Eghbali N. (2010). Green Chemistry: Principles
and Practice. Chem. Soc. Rev..

[20] Huh D. H., Jeong J. S., Lee H. B., Ryu H., Kim Y. G. (2002). An Efficient
Method for One-Carbon Elongation of Aryl Aldehydes via Their Dibromoalkene
Derivatives. Tetrahedron.

[21] Ma X., Herzon S. B. (2016). Synthesis of Ketones
and Esters from Heteroatom-Functionalized
Alkenes by Cobalt-Mediated Hydrogen Atom Transfer. J. Org. Chem..

[22] Horie K., Barón M., Fox R. B., He J., Hess M., Kahovec J., Kitayama T., Kubisa P., Maréchal E., Mormann W., Stepto R. F. T., Tabak D., Vohlídal J., Wilks E. S., Work W. J. (2004). Definitions of terms relating to
reactions of polymers and to functional polymeric materials (IUPAC
Recommendations 2003). Pure Appl. Chem..

[23] Rightmire N. R., Hanusa T. P. (2016). Advances in organometallic
synthesis with mechanochemical
methods. Dalton Trans..

[24] Balema V. P., Wiench J. W., Pruski M., Pecharsky V. K. (2002). Mechanically
Induced Solid-State Generation of Phosphorus
Ylides and the Solvent-Free Wittig Reaction. J. Am. Chem. Soc..

[25] Marrett J., Titi H. M., Teoh Y., Friščić T. (2023). Supramolecular
“baking powder”: a hexameric halogen-bonded phosphonium
salt cage encapsulates and functionalizes small-molecule carbonyl
compounds. Chem. Sci..

[26] Batesky D. C., Goldfogel M. J., Weix D. J. (2017). Removal of Triphenylphosphine Oxide
by Precipitation with Zinc Chloride in Polar Solvents. J. Org. Chem..

[27] Lincoln S., Pisaniello D., Spotswood T., Tkaczuk M. (1981). A Phosphorus-31 Nuclear
Magnetic Resonance Study of Triphenylphosphine Oxide Exchange on Magnesium­(II)
and Zinc­(II). Aust. J. Chem..

[28] Ananthnag G. S., Mague J. T., Balakrishna M. S. (2015). A Cyclodiphosphazane Based Pincer
Ligand, [2,6-{μ-(t BuN) 2 P­(t BuHN)­PO} 2 C 6 H 3 I]: Ni II,
Pd II, Pt II and Cu I Complexes and Catalytic Studies. Dalton Trans..

[29] Guino-o M. A., Bustrom B., Tigaa R. A., de Bettencourt-Dias A. (2017). Microwave-Assisted
Synthesis of Ternary Lanthanide­(2-Thenoyltrifluoroacetone)­3­(Triphenylphosphine
Oxide)­2 Complexes. Inorg. Chim. Acta.

[30] Hoffmann R. W., Bovicelli P. (1990). Conversion
of Aldehydes into 1,1-Dibromoalkanes. Synthesis.

[31] Zhang W., Xu W., Zhang F., Li J., Liu M., Li W. (2012). Synthesis
of 4-(2,2-dibromovinyl)­phenol and its one-pot esterification. Rev. Chem. Intermed..

[32] Kushto G. P., Jagodziński P. W. (2000). Formation
of a ground state twisted-internal-charge-transfer conformer of 4-(dimethylamino)­benzaldehyde. J. Mol. Struct..

[33] Hack D., Chauhan P., Deckers K., Mizutani Y., Raabe G., Enders D. (2015). Combining silver- and organocatalysis: an enantioselective
sequential catalytic approach towards pyrano-annulated pyrazoles. Chem. Commun..

[34] Yang Y., Kuang C., Jin H., Yang Q., Zhang Z. (2011). Efficient
synthesis of 1,3-diaryl-4-halo-1H-pyrazoles from 3-arylsydnones and
2-aryl-1,1-dihalo-1-alkenes. Beilstein J. Org.
Chem..

[35] Hwang G. T., Son H. S., Ku J. K., Kim B. H. (2003). Synthesis and Photophysical
Studies of Bis-enediynes as Tunable Fluorophores. J. Am. Chem. Soc..

[36] Michaelides I. N., Darses B., Dixon D. J. (2011). Acid-Catalyzed
Synthesis of Bicyclo­[3.n.1]­alkenediones. Org.
Lett..

[37] Gimeno N., Martín-Rapún R., Rodríguez-Conde S., Serrano J. L., Folcia C. L., Pericás M. A., Ros M. B. (2012). Click chemistr as a versatile route
to synthesize and
modulate bent-core liquid crystalline materials. J. Mater. Chem..

[38] Domino K., Veryser C., Wahlqvist B. A., Gaardbo C., Neumann K. T., Daasbjerg K., De Borggraeve W. M., Skrydstrup T. (2018). Direct Access
to Aryl Bis­(trifluoromethyl)­carbinols from Aryl Bromides or Fluorosulfates:
Palladium-Catalyzed Carbonylation. Angew. Chem.,
Int. Ed..

[39] Moodapelly S. K., Sharma G. V. M., Doddi V. R. (2017). Controlled
Reactivity of 1,8-Diazabicyclo[5.4.0]­undec-7-ene
(DBU) in the Selective Synthesis of 1-(Bromoethynyl)­arenes. Adv. Synth. Catal..

[40] Okutani M., Mori Y. (2009). Conversion of Bromoalkenes into Alkynes by Wet Tetra-n-butylammonium
Fluoride. J. Org. Chem..

[41] Shen W., Kohn T., Fu Z., Jiao X., Lai S., Schmitt M. (2008). Synthesis of benzimidazoles from 1,1-dibromoethenes. Tetrahedron Lett..

[42] Rao M. L. N., Islam S. (2021). Synthesis
of alkynes under dry reaction conditions. Tetrahedron
Lett..

