# Supplementary material for: Reinventing the Corey–Fuchs Reaction under Ball Milling Conditions: A Mechanochemical Pathway to Bromoalkynes
Source: ACS Omega. 2026 Jul 24;11(31):47060–70. doi: 10.1021/acsomega.6c06178 (PMC13470746; doi:10.1021/acsomega.6c06178)
Supplement: Supplementary file 1 [file ao6c06178_si_001.pdf]

Supporting Information for

Reinventing the Corey–Fuchs Reaction under Ball Milling Conditions: A Mechanochemical Pathway to Bromoalkynes

Barbara Kaczmarek, Ireneusz Kownacki*, Dawid Frąckowiak*

Barbara Kaczmarek, Ireneusz Kownacki

Faculty of Chemistry, Adam Mickiewicz University, Poznań, Poland
Department of Chemistry and Technology of Silicon Compounds

E-mail: *ireneusz.kownacki@amu.edu*

Dawid Frąckowiak, Ireneusz Kownacki

Center for Advanced Technologies, Adam Mickiewicz University, Poznań, Poland.

E-mail: *dawidoff@amu.edu.pl*

1. General considerations **3**
1.1. Reagents 3
2. Mechanochemical synthesis of dibromoalkenes **4**
2.1. Initial tests of the Corey-Fuchs reaction on a scale of 0.05 g of 4-nitrobenzaldehyde **4**
2.2. PXRD analyses **6**
2.3. GC-MS analyses **9**
2.4. General procedure for the synthesis of 1,1-dibromoalkenes on a scale of 0.2 g of aldehyde **13**
2.5. General procedure for the synthesis of 1-(2,2-dibromovinyl)-4-nitrobenzene on a scale of 1 g of aldehyde **13**
2.6. Solid-state synthesis of [$ZnCl_2(Ph_3P=O)_2$] complex **13**
2.7. 1,1-Dibromoalkene products and [$ZnCl_2(Ph_3P=O)_2$] – details and spectral data **14**
1-(2,2-Dibromovinyl)-4-nitrobenzene [4a] 14
1-(2,2-Dibromovinyl)-4-bromobenzene [4b] 14
2-(2,2-Dibromovinyl)-naphthalene [4c] 14
1,4-Bis(2,2-dibromovinyl)-benzene [4d] 14
1-(2,2-Dibromovinyl)-4-benzonitrile [4e] 15
Methyl 4-(2,2-Dibromovinyl)-benzoate [4f] 15
1,4-Bis(2,2-dibromoethenyl)benzene [4g] 15
9-(2,2-Dibromovinyl)anthracene [4h] 15
(E)-(4,4-dibromobuta-1,3-dien-1-yl)benzene [4i] 16
tert-Butyl 3-(2,2-dibromovinyl)-1H-indole-1-carboxylate [4j] 16
6-(2,2-Dibromovinyl)-2,3-dihydrobenzo[b][1,4]dioxine [4k] 16
Dichlorobis(triphenylphosphine oxide)zinc(II) [5] 17
2.8. General procedure for the synthesis of 1-bromoalkynes on a scale of 0.1 g of 1,1-dibromoalkene . **17**
2.9. General procedure for the synthesis of 1-(bromoethynyl)-4-nitrobenzene on a scale of 1 g of 1,1-dibromoalkene in a mixer mill **17**
2.10. General procedure for the synthesis of 1-(bromoethynyl)-4-nitrobenzene on a scale of 1 g of 1,1-dibromoalkene in a planetary mill **17**
2.11. 1-Bromoalkyne products – details and spectral data **18**
1-(Bromoethynyl)-4-nitrobenzene [6a] 18
1-(Bromoethynyl)-4-bromobenzene [6b] 18
2-(Bromoethynyl)naphthalene [6c] 18
4-(Bromoethynyl)-1,1'-biphenyl [6d] 18
4-(Bromoethynyl)benzonitrile [6e] 19
1,4-Bis(2,2-dibromoethenyl)benzene [6g] 19
9-(Bromoethynyl)anthracene [6h] 19
2.12. Green metrics **20**
3. Literature **20**
4. ^{1}H, $^{13}C\{^{1}H\}$, $^{31}P\{^{1}H\}$ NMR, MS (EI) and FT-IR spectra **20**

1. General considerations

1.1. Reagents

The chemicals were purchased from the following sources:

- **AmBeed:** 2-naphthaldehyde, 9-anthraldehyde, 4-formylbenzonitrile, terephthaldehyde, succinimide;
- **Across Organics:** 4-nitrobenzaldehyde, 4-hydroxybenzaldehyde, biphenyl-4-carboxaldehyde, carbon tetrabromide, triphenylphosphine, Cs_2CO_3;
- **Angene:** $CsOH \cdot H_2O$, $[Me_4N]OH \cdot H_2O$, DBU;
- **Apollo Scientific:** methyl 4-formylbenzoate;
- **Fluorochem:** triphenylphosphine dibromide, helional, 4-dimethylaminopyridine;
- **Maybridge:** 4-bromobenzaldehyde, 1,4-benzodioxan-6-carboxaldehyde;
- **Sigma-Aldrich:** cinnamaldehyde, *tert*-butyl 3-formyl-1*H*-indole-1-carboxylate, ^{t}BuOK;
- **Chemat:** 3-(dimethylamino)benzaldehyde
- **Warchem:** benzaldehyde;
- **Fischer Chemicals:** dichloromethane, ethyl acetate, 4-dimethylaminobenzaldehyde, 2-pyridinecarboxaldehyde, K_2CO_3, K_3PO_4, Al_2O_3 (basic), DMSO;
- **Chempur:** ethanol, petroleum ether, zinc chloride (anhydrous), KOH, NaOH;
- **Deutero:** chloroform-d$_7$.

Most reagents were used as received; CBr_4 and 1,4-benzodioxan-6-carboxaldehyde were sublimated; PPh_3 was recrystallized from ethanol; benzaldehyde and cinnamaldehyde were distilled under reduced pressure.

2. Mechanochemical synthesis of dibromoalkenes

2.1. Initial tests of the Corey-Fuchs reaction on a scale of 0.05 g of 4-nitrobenzaldehyde

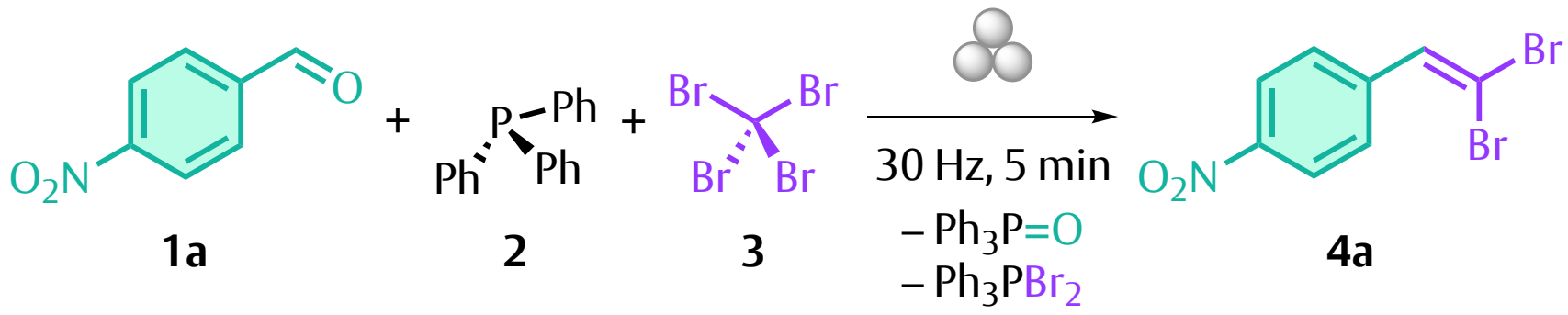

[RCHO] : [PPh$_3$] : [CBr$_4$] = 1 : 2 : 2 | SS jars V = 10 mL; 1 SS ball ϕ = 10 mm

Scheme S1. Mechanochemical CF reaction in a model system.

A 10 mL stainless steel milling jar was charged with 4-nitrobenzaldehyde (0.050 g, 0.33 mmol), CBr_4 (0.66 mmol), PPh_3 (0.66 mmol), and one stainless steel ball (Φ = 10 mm).

The powders were ball milled for 5 min at 30 Hz; the crude was then scraped from the milling jar using a smartSpatula® and analyzed by GC-MS (Figure S1). The differences between reaction mixtures before and after reaction are depicted in Picture S1.

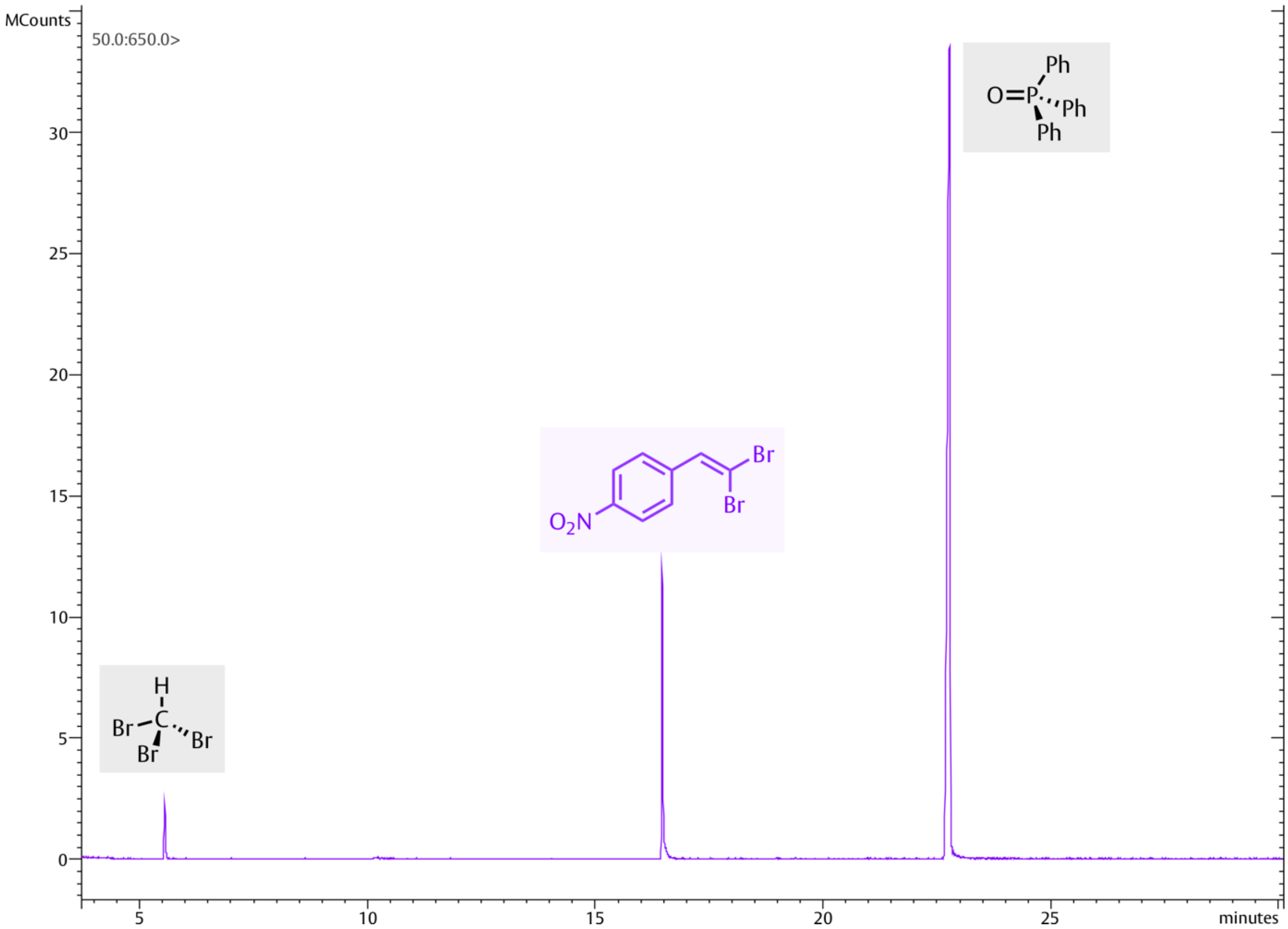

Figure S1. Chromatogram of the post-reaction mixture after ball milling of 4-nitrobenzaldehyde with CBr_4 and PPh_3.

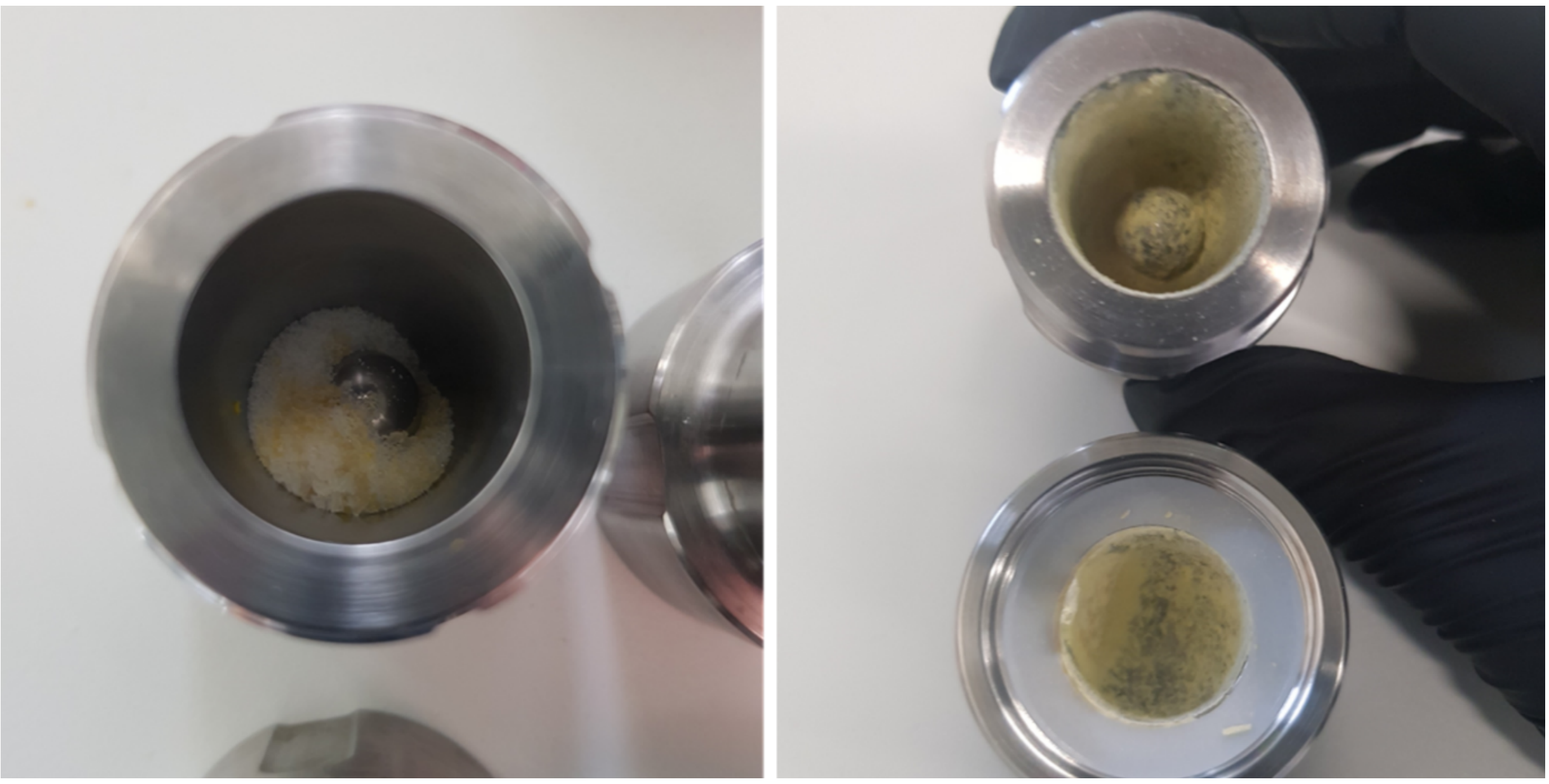

Picture S1. The appearance of the reaction mixture before and after milling.

2.2. PXRD analyses

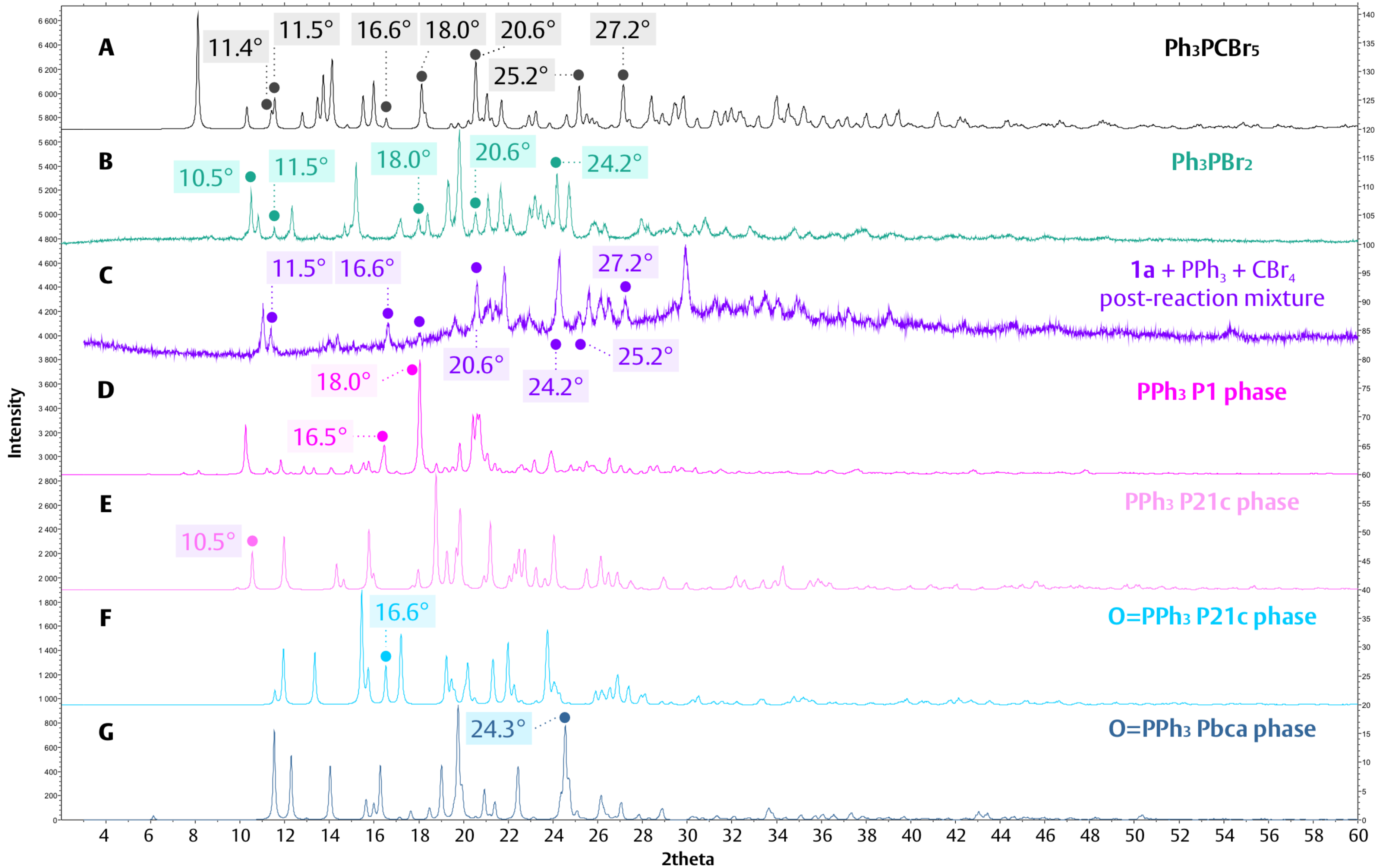

Figure S2. PXRD analysis of the post-reaction mixture containing **1a**, **PPh_3**, **CBr_4** [C] and potential phosphorus compounds that might be present: **Ph_3PCBr_5** [A] is formally a dimeric structure, containing **$[Ph_3PCBr_3]^+$**, **$[Br_3]^-$** and **Br^-** ions; it is formed by oxidation of **$[Ph_3PCBr_3]^+Br^-$**,[1] **Ph_3PBr_2** [B], **PPh_3** (P1 phase [D], P21c phase [E]) and **$Ph_3P{=}O$** (P21c phase [F], Pbca phase [G]). Diffractograms of **Ph_3PCBr_5**, **PPh_3** and **$O{=}PPh_3$** are simulated.

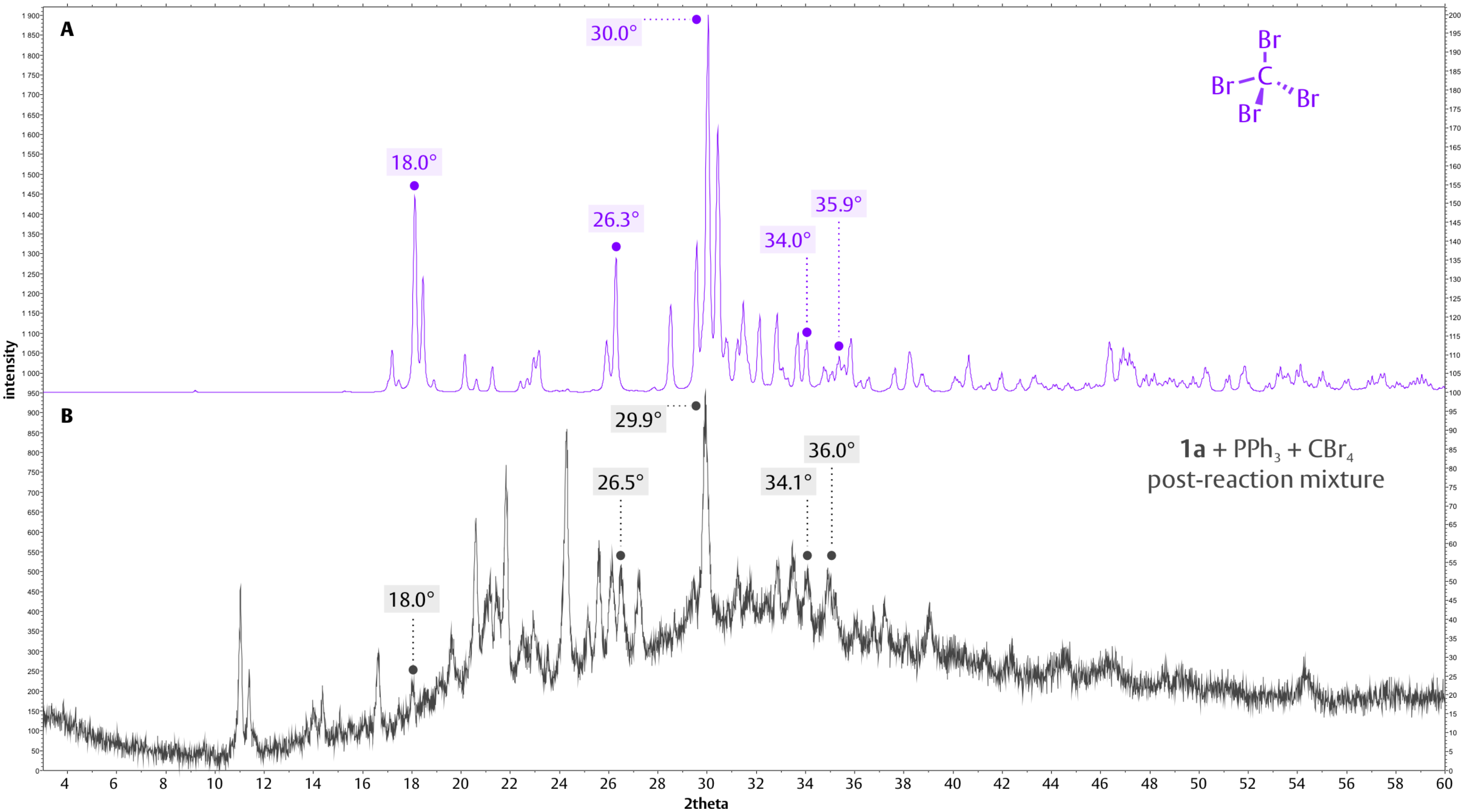

Figure S3. PXRD analysis of the post-reaction mixture containing **1a**, **PPh₃**, **CBr₄** [B] – identification of **CBr₄** [A].

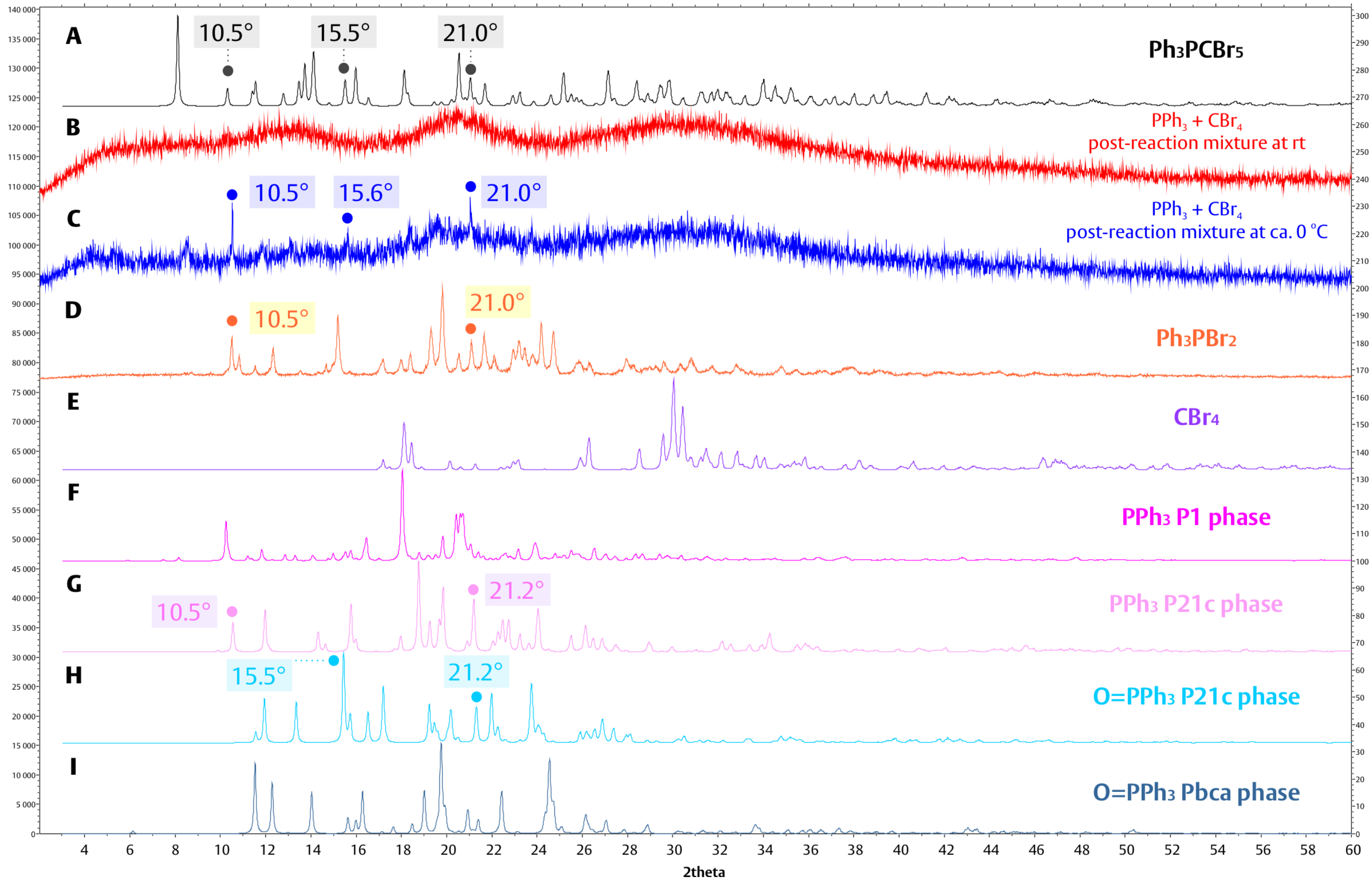

Figure S4. PXRD analysis of the post-reaction mixture after ball milling of equimolar amounts of **PPh_3**, **CBr_4** (at room temp. [B], at 0 °C [C]), **CBr_4** [E] and potential phosphorus compounds that might be present: **Ph_3PCBr_5** [A], **Ph_3PBr_2** [D], **PPh_3** (P1 phase [F], P21c phase [G]) and **$Ph_3P{=}O$** (P21c phase [H], Pbca phase [I]). Diffractograms of **Ph_3PCBr_5**, **PPh_3**, and **$O{=}PPh_3$** are simulated.

2.3. GC-MS analyses

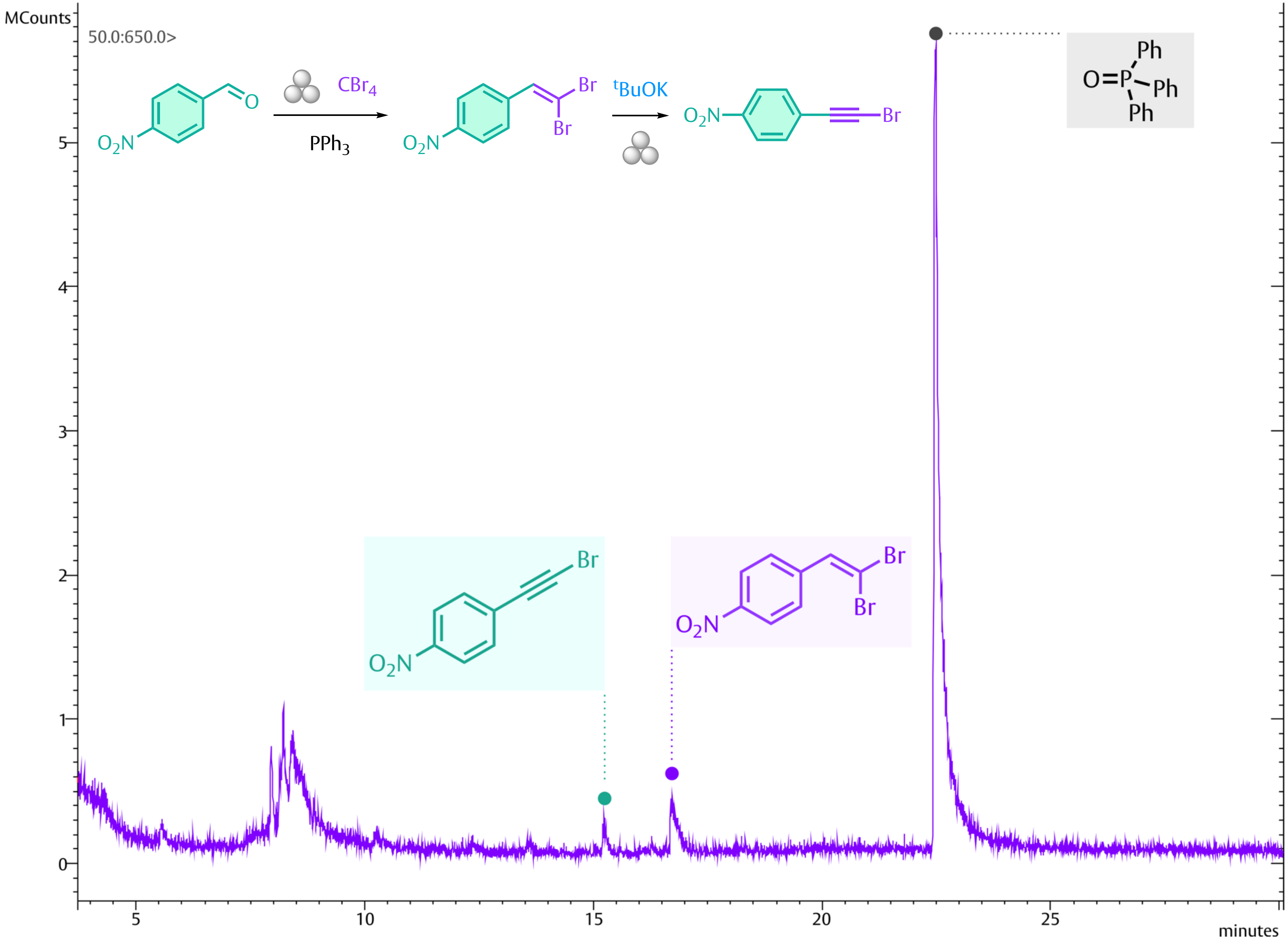

Figure S5. GC-MS analysis of the attempted mechanochemical one-pot Corey-Fuchs reaction. [RCHO] : [PPh_3] : [CBr_4] = 1 : 4 : 2; [$RCH{=}CBr_2$] : [$^{t}BuOK$] = 1 : 2; 30 Hz, 1 h, SS jars **V = 10 mL**; 1 SS ball **ϕ = 10 mm**.

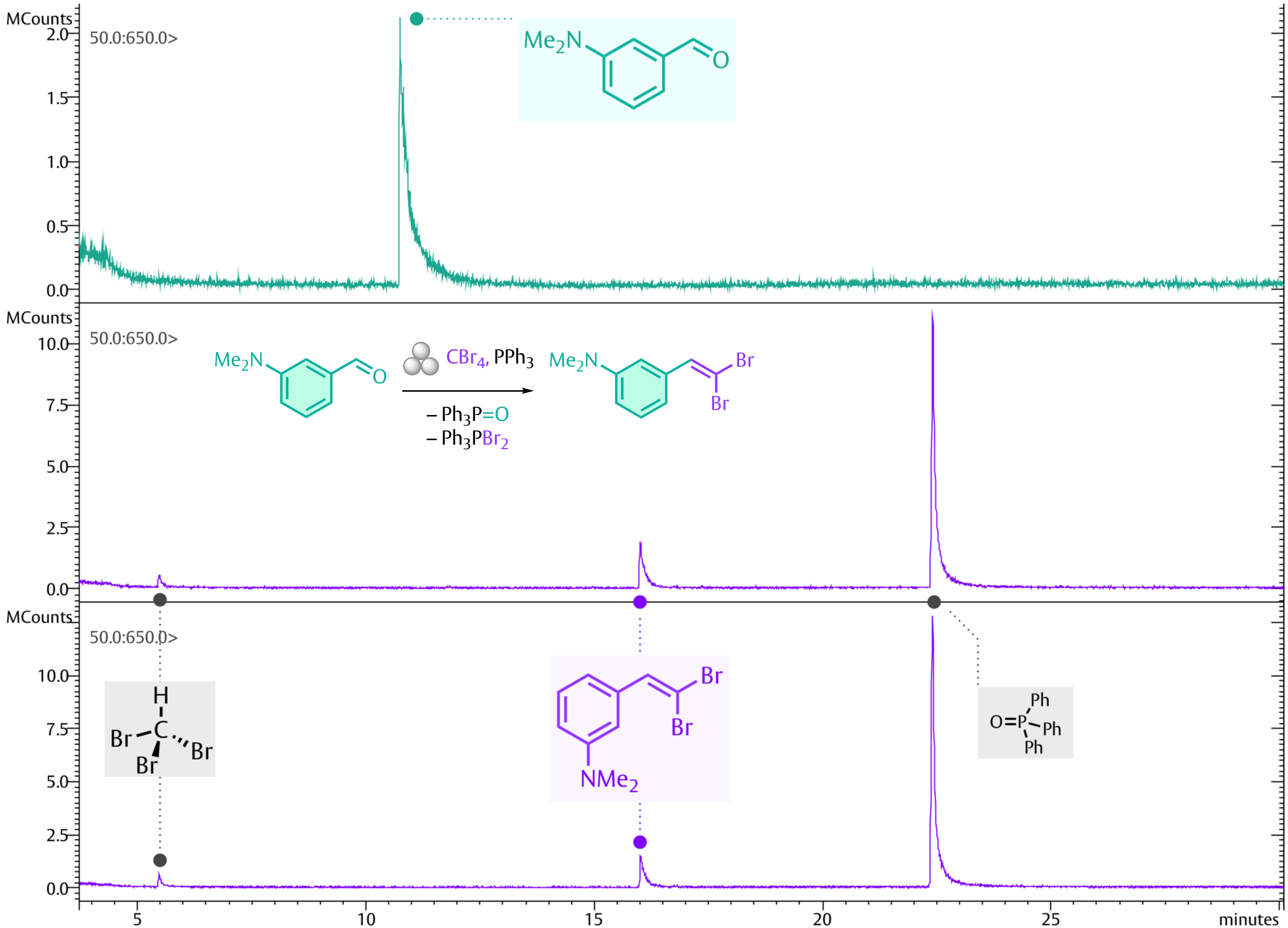

Figure S6. GC-MS analysis after ball milling of 3-(dimethylamino)benzaldehyde **1q** with **PPh₃** and **CBr₄**. [RCHO] : [PPh_3] : [CBr_4] = 1 : 2 : 2; 30 Hz, 1 h, SS jars **V = 10 mL**; 1 SS ball **ϕ = 10 mm**.

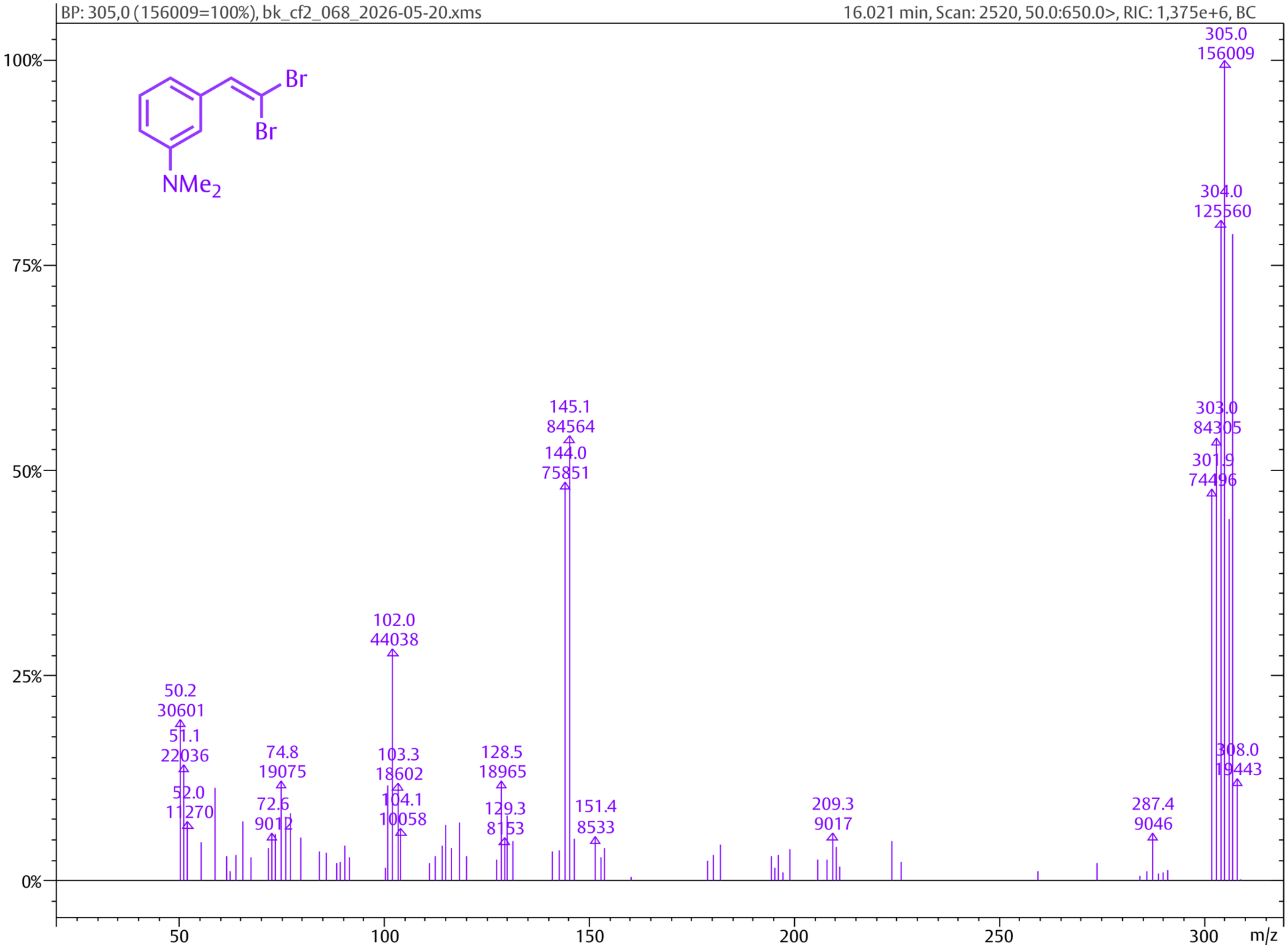

Figure S7. Mass spectrum of 3-(2,2-dibromovinyl)-*N*,*N*-dimethylaniline **4q**.

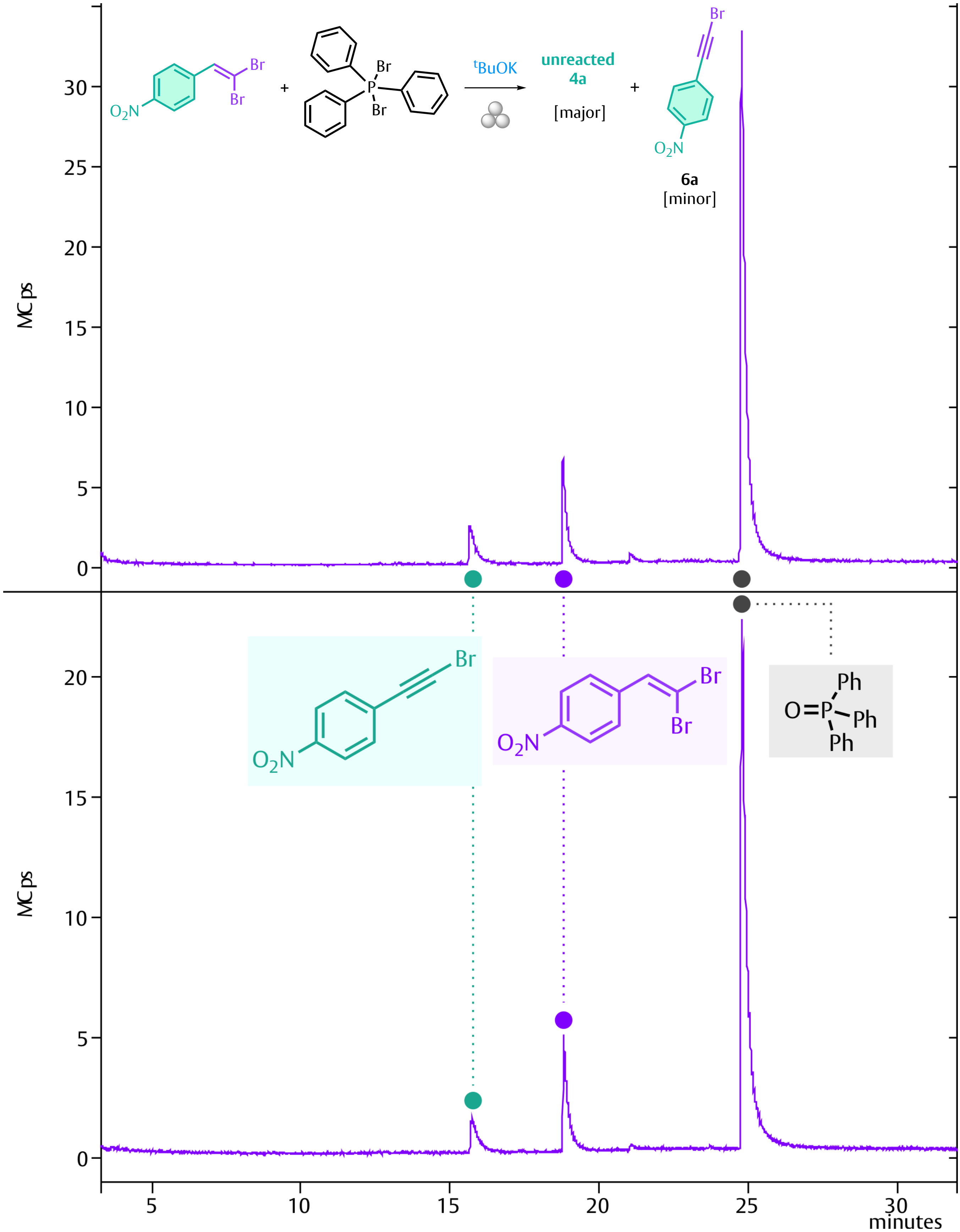

Figure S8. GC-MS analysis after ball milling of pure **4a** with **Ph_3PBr_2** in the presence of **^{t}BuOK**. [**4a**] : [Ph_3PBr_2] : [^{t}BuOK] = 1 : 1 : 2; 30 Hz, 1 h, SS jars **V = 10 mL**; 1 SS ball **ϕ = 10 mm**.

2.4. General procedure for the synthesis of 1,1-dibromoalkenes on a scale of 0.2 g of aldehyde

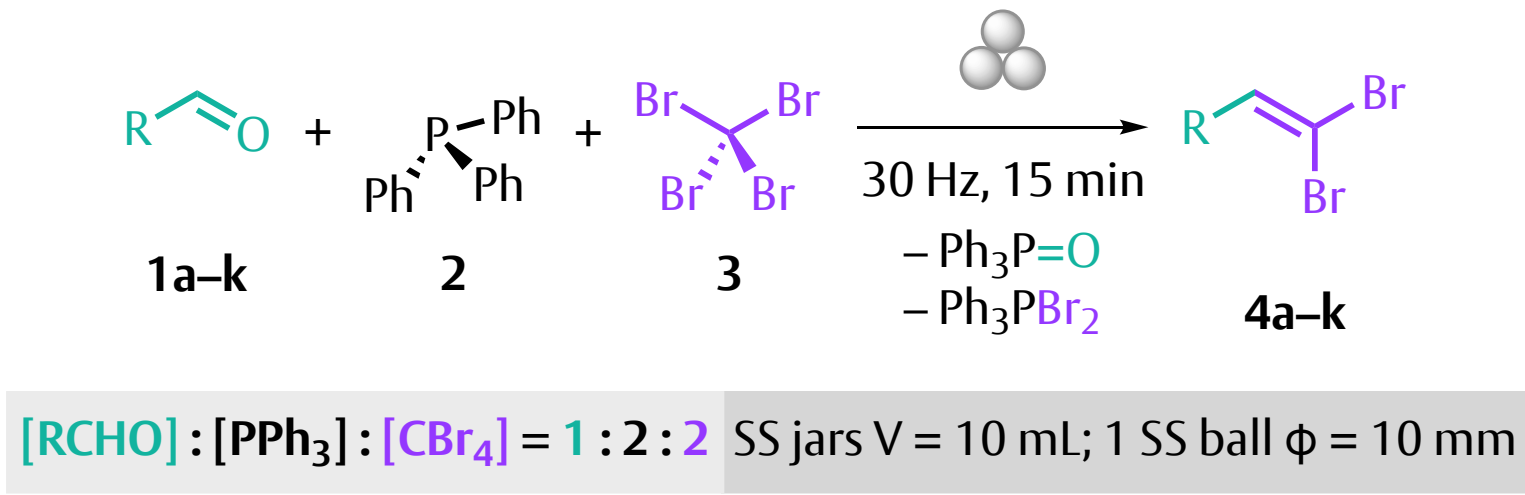

Scheme S2. Mechanochemical synthesis of 1,1-dibromoalkenes.

A 10 mL stainless steel milling jar was charged with an aldehyde (0.200 g), CBr_4, and PPh_3 in a molar ratio RCHO : CBr_4, PPh_3 [1 : 2 : 2] and one stainless steel ball (Φ = 10 mm). The powders were ball milled for 15 min at 30 Hz. Then, the product was scraped from the milling jar using a smartSpatula® and dissolved in a minimal amount of DCM (8 mL) to prepare a dry load from the post-reaction mixture and Celite. The dry load prepared in this way was applied to a silica gel column and eluted with petroleum ether, followed by petroleum ether : EtOAc [90 : 10]. The fractions containing the desired product were then combined and concentrated in vacuo. The pure product was dried using a Schlenk line and characterized by MS, FT-IR, and ^{1}H and $^{13}C\{^{1}H\}$ NMR spectroscopy.

2.5. General procedure for the synthesis of 1-(2,2-dibromovinyl)-4-nitrobenzene on a scale of 1 g of aldehyde

A 50 mL stainless steel milling jar was charged with 1 g (6.62 mmol) of 4-nitrobenzaldehyde, 4.389 g (13.23 mmol) CBr_4, 3.471 g (13.23 mmol) PPh_3, and ten stainless steel balls (Φ = 10 mm). The powders were ball milled for 30 min at 30 Hz; the product was then scraped from the milling jar using smartSpatula®. Subsequently, the powdery post-reaction mixture was applied as a dry load to a silica gel column and eluted with petroleum ether, followed by a petroleum ether-EtOAc mixture (90 : 10). The fractions containing the desired product were then combined and concentrated in vacuo. The pure product was dried using a Schlenk line and characterized by MS, FT-IR, ^{1}H, and $^{13}C\{^{1}H\}$ NMR spectroscopy.

2.6. Solid-state synthesis of [$ZnCl_2(Ph_3P{=}O)_2$] complex

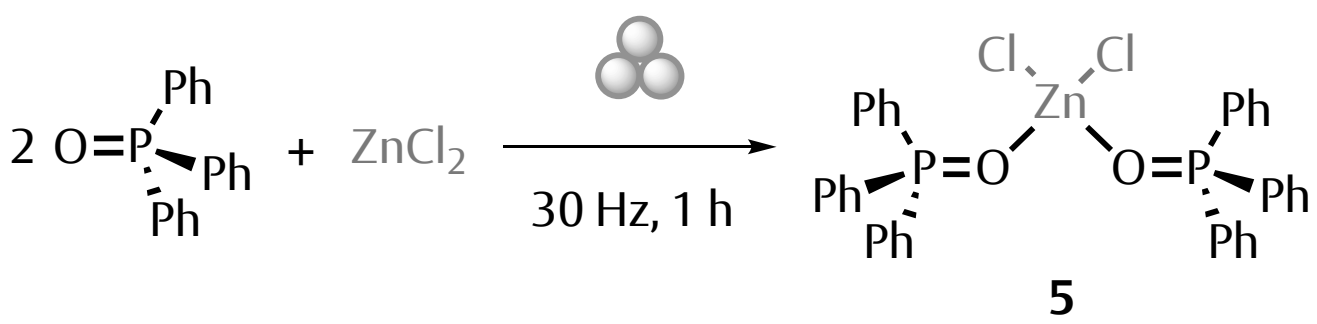

Scheme S3. Mechanochemical synthesis of [$ZnCl_2(Ph_3P{=}O)_2$].

Tests were performed in stainless steel vessels (V = 10 mL) with one stainless steel grinding ball (ϕ = 10 mm) at a scale of 0.4 g TPPO. The use of f = 30 Hz and a grinding time of 1 h in a mixer mill enabled the complete conversion of TPPO to [$ZnCl_2(Ph_3P{=}O)_2$], as confirmed by ^{1}H and ^{31}P NMR analysis (Figures S54–S55). The complex formation occurred completely at the molar ratio of TPPO : $ZnCl_2$ [2 : 1]. When the reaction time was reduced to 30 min, TPPO was not fully converted to the complex. The pure product was characterized by ^{1}H and $^{31}P\{^{1}H\}$ NMR spectroscopy.

2.7. 1,1-Dibromoalkene products and [$ZnCl_2(Ph_3P{=}O)_2$] – details and spectral data

1-(2,2-Dibromovinyl)-4-nitrobenzene [4a]

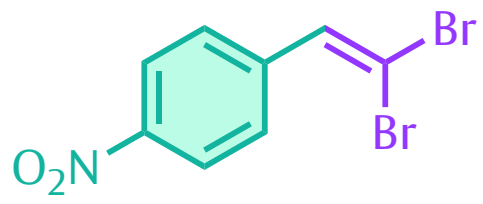

The product was prepared according to general procedures using 0.200 g (1.33 mmol) of 4-nitrobenzaldehyde, 0.878 g (2.64 mmol) of CBr_4, 0.694 g (2.64 mmol) of PPh_3 and obtained as a pale yellow solid (0.309 g, 76%; on a scale of 1 g: 1.638 g, 81%). **^{1}H NMR** (300 MHz, $CDCl_3$, ppm): δ 8.23 (d, *J* = 8.9 Hz, 2H), 7.70 (d, *J* = 8.9 Hz, 2H), 7.56 (s, 1H, C<u>H</u>=CBr_2); **^{13}C{^{1}H} NMR** (75 MHz, $CDCl_3$, ppm): δ 147.21(<u>C</u>H=), 141.45, 134.89, 129.19, 123.73, 94.09 (=<u>C</u>Br_2); **MS (EI):** m/z (%rel. int.) = 306.96 (100%) [M^+]. **FT-IR** (cm^{-1}): 3022 (=C–H), 1585.20 (C=C arom.), 1487.81 (N–O), 1331.61 (C–N), 685.09 (C–Br).

1-(2,2-Dibromovinyl)-4-bromobenzene [4b]

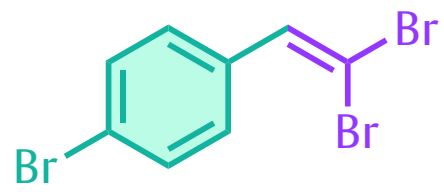

The product was prepared according to general procedures using 0.200 g (1.08 mmol) of 4-bromobenzaldehyde, 0.716 g (2.16 mmol) of CBr_4, 0.566 g (2.16 mmol) of PPh_3, and obtained as a very pale yellow solid (0.332 g, 90%). **^{1}H NMR** (300 MHz, $CDCl_3$): δ 7.55 (d, *J* = 8.9 Hz, 2H), 7.47 (s, 1H, C<u>H</u>=CBr_2), 7.45 (d, *J* = 8.9 Hz, 2H).; **^{13}C{^{1}H} NMR** (75 MHz, $CDCl_3$) δ 135.78 (<u>C</u>H=), 134.22, 131.69, 129.96, 122.68, 90.61 (=<u>C</u>Br_2). **MS (EI):** m/z (%rel. int.) = 341.98 (100%) [M^+]. **FT-IR** (cm^{-1}): 3008.89 (=C–H),1483.96 (C=C arom.), 566.49 (C–Br).

2-(2,2-Dibromovinyl)-naphthalene [4c]

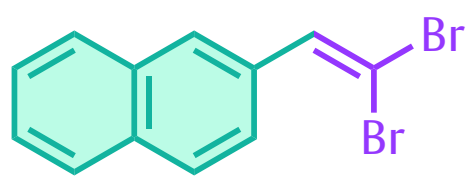

The product was prepared according to the general procedures using 0.2 g (1.28 mmol) of 2-naphthaldehyde, 0.8490 g (2.56 mmol) of CBr_4, 0.6714 g (2.56 mmol) of PPh_3 and obtained as a white solid (376 mg, 94%). **^{1}H NMR** (300 MHz, $CDCl_3$): δ 8.04 (s, 1H, C<u>H</u>=CBr_2), 7.93–7.81 (m, 3H), 7.71–7.64 (m, 2H), 7.60–7.48 (m, 2H); **^{13}C{^{1}H} NMR** (75 MHz, $CDCl_3$, ppm): δ 136.9 (<u>C</u>H=), 133.0, 133.0, 132.8, 128.3, 128.1, 128.0, 127.7, 126.8, 126.5, 125.6, 89.9 (=<u>C</u>Br_2); **MS (EI):** m/z (%rel. int.) = 311.99 (33%) [M^+], 152 (100%). **FT-IR** (cm^{-1}): 3010.82 (=C–H), 1505.17 (C=C arom.), 709.19 (C=C), 587.22 (C–Br).

1,4-Bis(2,2-dibromovinyl)-benzene [4d]

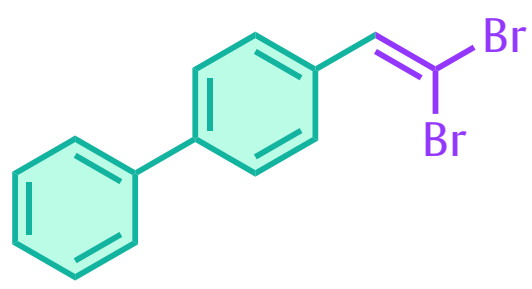

The product was prepared according to general procedures using 0.200 g (1.10 mmol), 0.730 g (2.20 mmol) of CBr_4, 0.577 g (2.20 mmol) of PPh_3, and obtained as a white solid (0.260 g, 70%). **^{1}H NMR** (300 MHz, $CDCl_3$): δ 7.69–7.56 (m, 6H), 7.52 (s, 1H, C<u>H</u>=CBr_2), 7.46 (t, *J* = 7.2 Hz, 2H), 7.37 (t, *J* = 7.2 Hz, 1H); **^{13}C{^{1}H} NMR** (75 MHz, $CDCl_3$, ppm): δ 141.4 (<u>C</u>H=), 140.5, 136.6, 134.4, 129.0, 127.8, 127.2, 89.6 (=<u>C</u>Br_2). **MS (EI):** m/z (%rel. int.) = 338.12 (92%) [M^+], 178.17 (100%). **FT-IR** (cm^{-1}): 3008.89 (=C–H), 1505.17 (C=C arom.), 759.82 (C=C), 650.38 (C–Br).

1-(2,2-Dibromovinyl)-4-benzonitrile [4e]

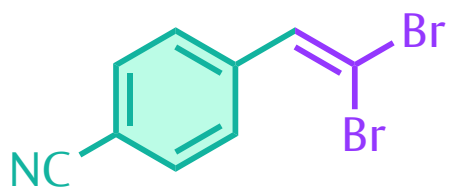

The product was prepared according to general procedures using 0.200 g (1.53 mmol) of 4-formylbenzonitrile, 1.012 g (3.06 mmol) of CBr_4, 0.803 g (3.06 mmol) of PPh_3, and obtained as a white solid (0.310 g, 70%). **^{1}H NMR** (300 MHz, $CDCl_3$): δ 7.69–7.65 (m, 2H), 7.65–7.61 (m, 2H), 7.50 (s, 1H, $C\underline{H}=CBr_2$); **^{13}C{^{1}H} NMR** (75 MHz, $CDCl_3$) δ 139.8 ($\underline{C}$H=), 135.3, 132.4, 129.1, 118.7, 112.15, 93.6 (=$\underline{C}Br_2$). **MS (EI):** m/z (%rel. int.) = 386.94 (38%) [M^+], 127.06 (100%). **FT-IR** (cm^{-1}): 3092.30 (=C–H), 2227.86 (C≡N) 1599.66 (C=C arom.), 1261.22 (C–N),718.84 (C=C), 547.68 (C–Br).

Methyl 4-(2,2-Dibromovinyl)-benzoate [4f]

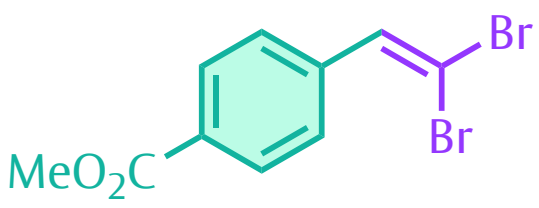

The product was prepared according to general procedures using 0.200 g (1.22 mmol) of methyl 4-formylbenzoate, 0.808 g (2.44 mmol) of CBr_4, 0.639 g (2.44 mmol) of PPh_3 and obtained as an off-white solid (0.312 g, 80%). **^{1}H NMR** (300 MHz, $CDCl_3$): δ 8.03 (m, 2H), 7.60 (m, 2H), 7.52 (s, 1H, $C\underline{H}=CBr_2$), 3.93 (s, 3H); **^{13}C{^{1}H} NMR** (75 MHz, $CDCl_3$): δ 166.71, 139.74, 136.19, 129.82, 128.48, 92.12 (=$\underline{C}Br_2$), 52.38. **MS (EI):** m/z (%rel. int.) = 320.02 (86%) [M^+], 289.07 (100%). **FT-IR** (cm^{-1}): 3007.44 (=C–H), 1712.00 (C=O), 1604.97 (C=C arom.), 762.23 (C=C), 642.66 (C–Br).

1,4-Bis(2,2-dibromoethenyl)benzene [4g]

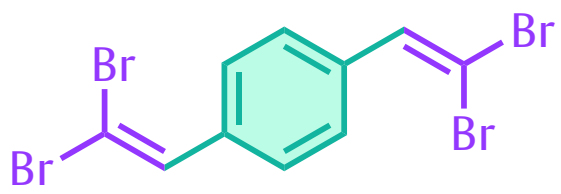

The product was prepared according to general procedures using 0.200 g (1.49 mmol) of terephthalaldehyde, 0.989 g (2.98 mmol) of CBr_4, 0.782 g (2.98 mmol) of PPh_3, and obtained as a white solid (0.445 g, 67%). **^{1}H NMR** (300 MHz, $CDCl_3$): δ 7.56 (s, 4H), 7.46 (s, 2H, $C\underline{H}=CBr_2$); **^{13}C{^{1}H} NMR** (75 MHz, $CDCl_3$) δ 136.36 ($\underline{C}$H=), 135.46, 128.57, 90.52 (=$\underline{C}Br_2$). **MS (EI):** m/z (%rel. int.) = 445.98 (63%) [M^+], 126.07 (100%). **FT-IR** (cm^{-1}): 3005.03 (=C–H), 1712.00 (C=O),1591.95 (C=C arom.), 736.67 (C=C), 574.68 (C–Br).

9-(2,2-Dibromovinyl)anthracene [4h]

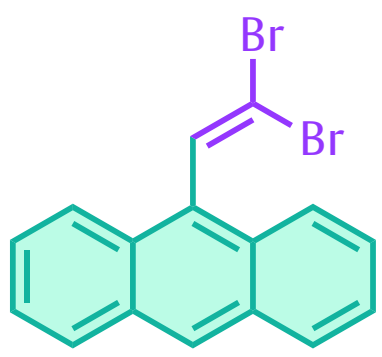

The product was prepared according to general procedures using 0.200 g (0.969 mmol) of anthracene-9-carbaldehyde, 0.509 g (1.94 mmol) of CBr_4, 0.643 g (1.94 mmol) of PPh_3, and obtained as a yellow solid (0.235 g, 67%). **^{1}H NMR** (300 MHz, $CDCl_3$): δ 8.50 (s, 1H, $C\underline{H}=CBr_2$), 8.15–8.01 (m, 5H), 7.60–7.46 (m, 4H). **^{13}C{^{1}H} NMR** (75 MHz, $CDCl_3$): δ 135.50, 131.35, 130.12, 128.87, 128.65, 127.89, 126.33, 125.49, 125.41, 125.01, 95.39, 77.46, 77.24, 77.03, 76.61. **MS (EI):** m/z (%rel. int.) = 361.97 (16.43%), 202.20 (100%). **FT-IR** (cm^{-1}): 3016.60 (=C–H), 1664.75 (C=C arom.), 825.87 (C=C), 686.05 (C–Br).

(*E*)-(4,4-dibromobuta-1,3-dien-1-yl)benzene [4i]

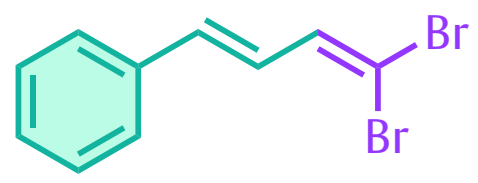

The product was prepared according to general procedures using 0.200 g (1.51 mmol) of cinnamaldehyde, 0.872 g (3.02 mmol) of CBr_4, 0.690 g (3.02 mmol) of PPh_3, and obtained as a colorless oil (0.296 g, 68%). The product contains trace impurity of aldehyde substrate (below 5%). **^{1}H NMR (300 MHz, $CDCl_3$):** δ 7.49–7.43 (m, 2H), 7.38–7.29 (m, 3H), 7.11 (d, *J* = 9.3 Hz, 1H, C<u>H</u>=CBr_2), 6.81 (dd, *J* = 15.6, 9.4 Hz, 1H), 6.71 (d, *J* = 15.6 Hz, 1H); **^{13}C{^{1}H} NMR** (75 MHz, $CDCl_3$): δ 137.26, 136.45, 135.84, 128.93, 128.74, 126.99, 125.38, 91.44 (=<u>C</u>Br_2); **MS (EI):** m/z (%rel. int.) = 288.00 (9.94%) [M^+], 128.16 (100%). **FT-IR** (cm^{-1}): 3015.16 (=C–H), 1558.20 (C=C arom.), 958.45 (C=C alkene), 743.42 (C=C), 687.02 (C–Br).

***tert*-Butyl 3-(2,2-dibromovinyl)-1*H*-indole-1-carboxylate [4j]**

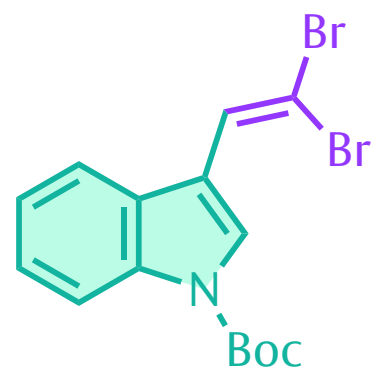

The product was prepared according to general procedures using 0.200 g (8.15 mmol) of *tert*-butyl 3-formyl-1*H*-indole-1-carboxylate, 0.428 g (16.3 mmol) of CBr_4, 0.541 g (16.3 mmol) of PPh_3, and obtained as an off-white solid (0.236 g, 72%). **^{1}H NMR** (300 MHz, $CDCl_3$): δ 8.33 (d, *J* = 0.9 Hz, 1H), 8.14 (d, *J* = 8.2 Hz, 1H), 7.60 (d, *J* = 0.9 Hz, 1H), 7.57 (d, *J* = 0.7 Hz, 1H), 7.40–7.26 (m, 2H), 1.69 (s, 9H). **^{13}C{^{1}H} NMR** (75 MHz, $CDCl_3$): δ 149.63, 134.61, 129.34, 127.59, 125.25, 125.03, 123.17, 118.48, 115.82, 115.48, 89.18, 84.59, 77.58, 76.74, 28.31. **MS (EI):** m/z (%rel. int.) = 300.99 (49.89%), 141.16 (100%). **FT-IR** (cm^{-1}): 3169.92 (=C–H), 2980.93 (–C–H), 1727.42 (C=O),1548.08 (C=C arom.), 1308.95 (C–N), 1147.44 (C–O), 574.68 (C–Br).

6-(2,2-Dibromovinyl)-2,3-dihydrobenzo[b][1,4]dioxine [4k]

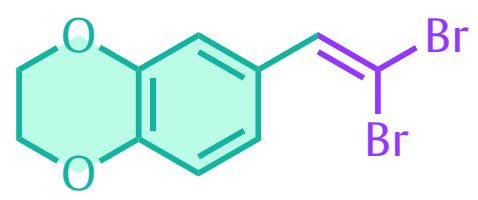

The product was prepared according to general procedures using 0.200 g (1.22 mmol) of 2,3-dihydrobenzo[b][1,4]dioxine-6-carbaldehyde, 0.808 g (2.44 mmol) of CBr_4, 0.639 g (2.44 mmol) of PPh_3 and obtained as a colorless oil (0.218 g, 56%). The product contains a trace impurity of the aldehyde substrate (below 5%). **^{1}H NMR** (300 MHz, $CDCl_3$): δ 7.34 (s, 1H, C<u>H</u>=CBr_2), 7.17 (dd, *J* = 2.1, 0.6 Hz, 1H), 7.01 (ddd, *J* = 8.5, 2.2, 0.6 Hz, 1H), 6.84 (d, *J* = 8.4 Hz, 1H), 4.27 (s, 4H). **^{13}C{^{1}H} NMR** (75 MHz, $CDCl_3$) δ 144.08, 143.34, 136.24, 128.75, 122.39, 117.30, 117.27, 87.89, 64.62, 64.41. **MS (EI):** m/z (%rel. int.) = 319.98 (100%) [M^+]. **FT-IR** (cm^{-1}): 2979.48 (=C–H), 2929.34 (–C–H), 1578.45 (C=C arom.), 1282.43 (C–O) 732.82 (C=C), 615.18 (C–Br).

Dichlorobis(triphenylphosphine oxide)zinc(II) [5]

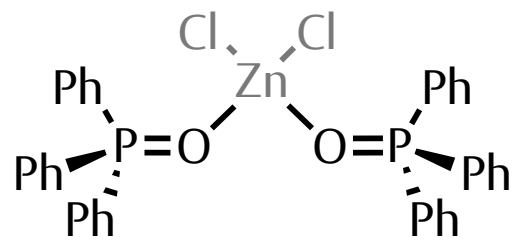

The product was prepared according to the described procedure using 0.400 g (1.44 mmol) of triphenylphosphine oxide, 0.0980 g (0.72 mmol) of anhydrous $ZnCl_2$, and obtained as a white solid (0.493 g, 99%). ^{1}**H NMR** (300 MHz, $CDCl_3$) δ 7.69–7.59 (m, 12H), 7.58–7.50 (m, 6H), 7.43–7.34 (m, 12H). ^{31}**P{^{1}H} NMR** (162 MHz, $CDCl_3$) δ 38.15.

2.8. General procedure for the synthesis of 1-bromoalkynes on a scale of 0.1 g of 1,1-dibromoalkene

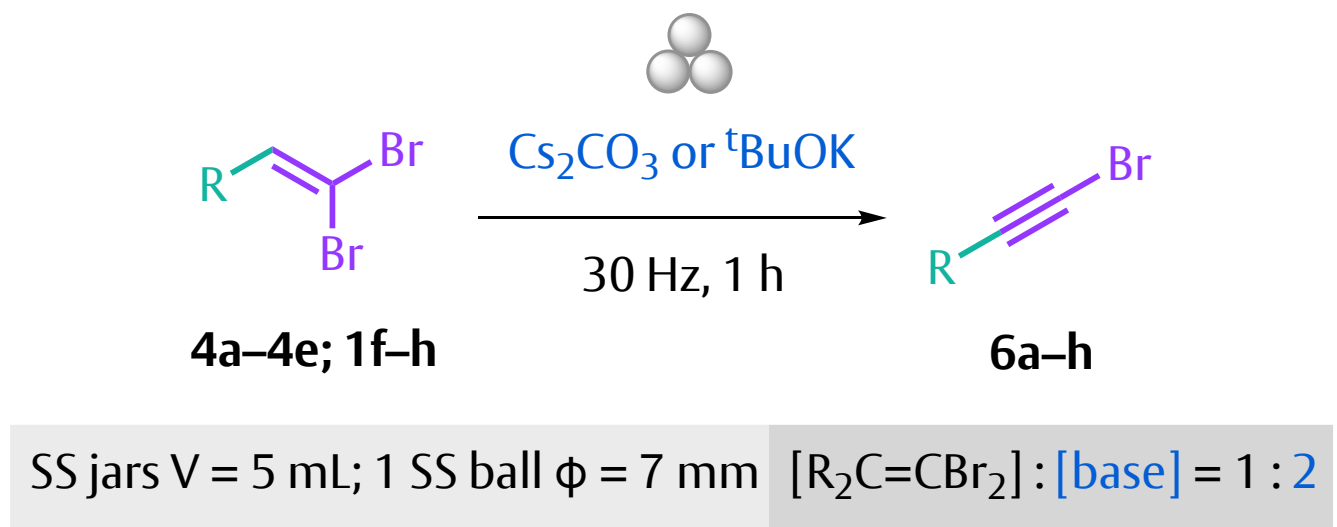

Scheme S4. General protocol for the mechanochemical synthesis of 1-bromoalkynes.

A 5 mL stainless steel milling jar was charged with 0.100 g of aldehyde, base (Cs_2CO_3 or ^{t}BuOK), and two stainless steel balls (Φ = 7 mm). The powders were ball milled for 1 h at 30 Hz, and the product was scraped from the milling jar using smartSpatula® and dissolved in a minimal amount of EtOAc (1 mL). The inorganic salt was filtered off using a funnel under reduced pressure. The pure product was dried using a Schlenk line and characterized by MS, FT-IR, and ^{1}H and ^{13}C{^{1}H} NMR spectroscopy.

2.9. General procedure for the synthesis of 1-(bromoethynyl)-4-nitrobenzene on a scale of 1 g of 1,1-dibromoalkene in a mixer mill

A 50 mL stainless steel milling jar was charged with 1 g of 4-nitrobenzaldehyde (3.26 mmol), 2.123 g Cs_2CO_3 (6.52 mmol), and seven stainless steel balls (Φ = 10 mm). The powders were ball-milled for 2.5 h at 30 Hz. Then, the product was scraped off the milling jar using a smartSpatula® and dissolved in a minimal amount of EtOAc (8 mL). The inorganic salt was filtered off using a funnel under reduced pressure. The pure product was dried using a Schlenk line and characterized by MS, FT-IR, and ^{1}H and ^{13}C{^{1}H} NMR spectroscopy.

2.10. General procedure for the synthesis of 1-(bromoethynyl)-4-nitrobenzene on a scale of 1 g of 1,1-dibromoalkene in a planetary mill

A 50 mL stainless steel milling jar was charged with 1 g of 4-nitrobenzaldehyde (3.26 mmol), 1.462 g ^{t}BuOK (13.04 mmol), and ten stainless steel balls (Φ = 10 mm). The powders were ball-milled for 2 h at 400 rpm, then the product was scraped from the milling jar using smartSpatula® and dissolved in a minimal amount of EtOAc (8 mL). The inorganic salt was filtered off using a funnel under reduced pressure. The pure product was dried using a Schlenk line and characterized by MS, FT-IR, and ^{1}H and ^{13}C{^{1}H} NMR spectroscopy.

2.11. 1-Bromoalkyne products — details and spectral data

1-(Bromoethynyl)-4-nitrobenzene [6a]

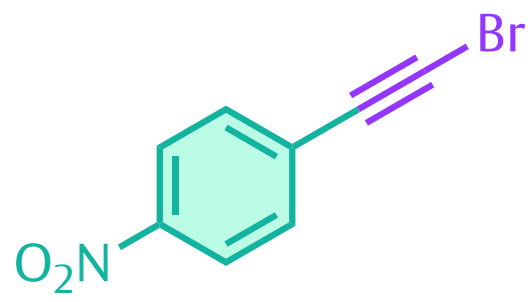

The product was prepared according to general procedures using 0.100 g (0.326 mmol) of **4a** and 0.212 g (0.652 mmol) of Cs_2CO_3, and obtained as a yellow solid (0.070 g, 95%); on a scale of 1 g: 0.662 g, 90% (mixer mill), 0.640 g, 87% (planetary ball mill). **^{1}H NMR** (300 MHz, $CDCl_3$): δ 8.22–8.16 (m, 2H), 7.63–7.56 (m, 2H); **^{13}C{^{1}H} NMR** (75 MHz, $CDCl_3$) δ 147.35, 132.85, 129.49, 123.64, 78.43, 56.39. **MS (EI):** *m/z* (%rel. int.) = 225.06 (100%) [M$^+$]. **FT-IR** (cm^{-1}): 3250.91 (≡C–H), 2194.11 (C≡C) 1927.02 (C–H arom.), 1590.02 (N–O), 1341.73 (C–N), 685.09 (C–Br).

1-(Bromoethynyl)-4-bromobenzene [6b]

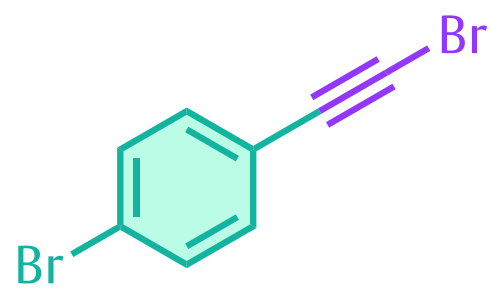

The product was prepared according to general procedures using 0.100 g (0.293 mmol) of **4b** and 0.191 g (0.586 mmol) of Cs_2CO_3, yielding a pale-yellow wax (72 mg, 94%). **^{1}H NMR** (300 MHz, $CDCl_3$): δ 7.48–7.27 (m, 4H); **^{13}C{^{1}H} NMR** (75 MHz, $CDCl_3$): δ 133.55, 131.78, 123.16, 121.78, 79.18, 77.58, 76.74, 51.36. **FT-IR** (cm^{-1}): 3068.19 (=C–H), 2196.52 (C≡C), 1579.90 (C=C arom.), 512.49 (C–Br).

2-(Bromoethynyl)naphthalene [6c]

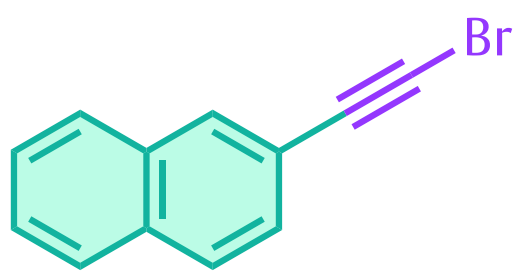

The product was prepared according to general procedures using 0.100 g (0.321 mmol) of **4c** and 0.108 g (0.963 mmol) of ^{t}BuOK, yielding a colorless oil (0.067 g, 90%). **^{1}H NMR** (300 MHz, $CDCl_3$): δ 7.99 (s, 1H), 7.85–7.75 (m, 3H), 7.54–7.45 (m, 3H); **^{13}C{^{1}H} NMR** (75 MHz, $CDCl_3$): δ 133.07, 132.96, 132.31, 128.53, 128.20, 127.91, 127.88, 127.05, 126.80, 120.10, 80.56, 77.58, 76.74, 50.16. **MS (EI):** m/z (%rel. int.) = 229.86 (100%). **FT-IR** (cm^{-1}): 3054.21 (=C–H), 2195.08 (C≡C), 1595.32 (C=C arom.), 471.51 (C–Br).

4-(Bromoethynyl)-1,1'-biphenyl [6d]

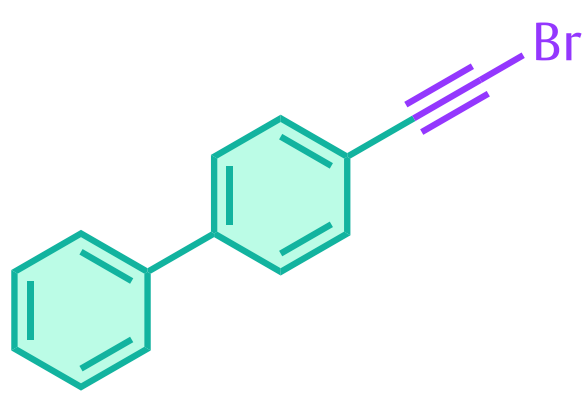

The product was prepared according to general procedures using 0.100 g (0.296 mmol) of **4d** and 0.100 g (0.888 mmol) of ^{t}BuOK, yielding a colorless oil (0.072 g, 95%). **^{1}H NMR** (300 MHz, $CDCl_3$): δ 7.63–7.49 (m, 6H), 7.48–7.41 (m, 2H), 7.40–7.32 (m, 1H); **^{13}C{^{1}H} NMR** (75 MHz, $CDCl_3$) δ 141.60, 140.36, 132.57, 129.03, 127.88, 127.17, 121.72, 80.09, 50.45. **MS (EI):** m/z (%rel. int.) = 256.20 (100%) [M$^+$]. **FT-IR** (cm^{-1}): 3028.66 (=C–H), 2159.88 (C≡C), 1596.29 (C=C arom.), 555.88 (C–Br).

4-(Bromoethynyl)benzonitrile [6e]

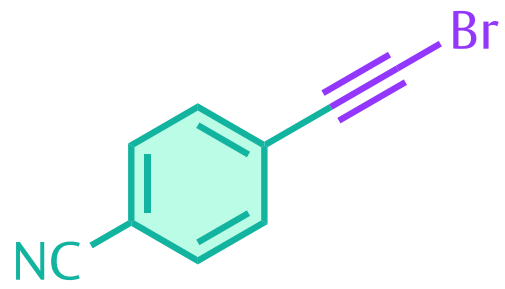

The product was prepared according to general procedures using 0.100 g (0.348 mmol) of **4e** and 0.227 g (0.696 mmol) of Cs_2CO_3 and obtained as a pale-yellow solid (0.066 g, 92%). **^{1}H NMR** (300 MHz, $CDCl_3$): δ 7.65–7.48 (m, 4H); **^{13}C{^{1}H} NMR** (75 MHz, $CDCl_3$): δ 132.70, 132.19, 127.68, 118.42, 112.25, 78.73, 55.53. **MS (EI):** m/z (%rel. int.) = 205.19 (100%) [M^+]. **FT-IR** (cm^{-1}): 3088.92 (=C–H), 2227.38 (C≡N), 2195.56 (C≡C), 1600.63 (C=C arom.), 548.65 (C–Br).

1,4-Bis(2,2-dibromoethenyl)benzene [6g]

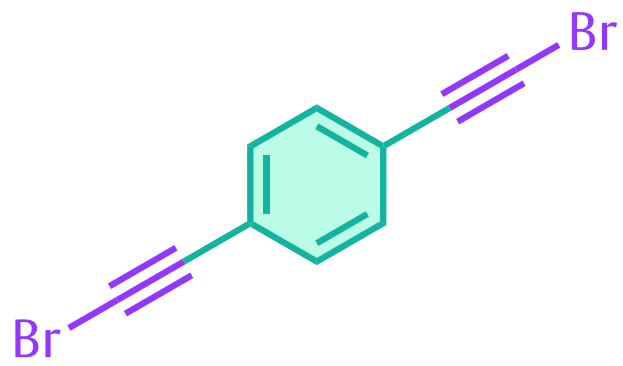

The product was prepared according to general procedures using 0.100 g (0.224 mmol) of **4g** and 0.101 g (0.896 mmol) of ^{t}BuOK and obtained as an off-white solid (0.057 g, 90%). **^{1}H NMR** (300 MHz, $CDCl_3$): δ 7.38 (s, 4H); **^{13}C{^{1}H} NMR** (75 MHz, $CDCl_3$): δ 132.05, 123.13, 79.69, 52.34. **MS (EI):** m/z (%rel. int.) = 284.02 (100%) [M^+]. **FT-IR** (cm^{-1}): 2922.11 (=C–H), 2195.08 (C≡C), 1655.11 (C=C arom.), 532.26 (C–Br).

9-(Bromoethynyl)anthracene [6h]

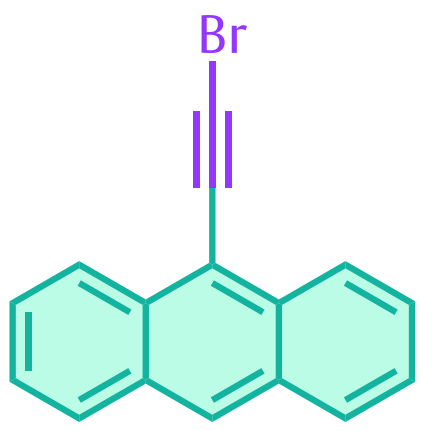

The product was prepared according to general procedures using 0.100 g (0.276 mmol) of **4h**, 0.093 g (0.828 mmol) of ^{t}BuOK and obtained as a brown solid (0.064 g, 82%). **^{1}H NMR** (300 MHz, $CDCl_3$): δ 8.51 (d, *J* = 1.0 Hz, 2H), 8.43 (s, 1H), 8.04–7.96 (m, 2H), 7.62–7.47 (m, 4H). **^{13}C{^{1}H} NMR** (75 MHz, $CDCl_3$): δ 133.29, 130.98, 128.66, 128.05, 126.85, 126.35, 125.67, 125.15, 59.58. **FT-IR** (cm^{-1}): 2922.11 (=C–H), 2175.79 (C≡C), 1450.21 (C=C arom.), 544.79 (C–Br).

2.12. Green metrics

The process mass intensity (PMI) and the environmental factor (E-factor) were calculated based on the model reaction with 4-nitrobenzaldehyde (1 g scale, Table S1) and 1-(2,2-dibromovinyl)-4-nitrobenzene (1 g scale, Table S2). For bromoalkyne synthesis in solution, the values were estimated according to the literature protocol.[2]

Table S1. Green metrics for the first step of the CF reaction of 4-nitrobenzaldehyde with CBr_4/PPh_3.

Synthesis method	Substrates [g]	Solvents, additives [g]	Product [g]	Other waste [g]	PMI	E-factor
Solvent	12.33	685.52	1.49	10.84	**468.4**	**467.4**
Mechanochemistry	8.86	381.22	1.62	7.24	**241.8**	**240.8**

Table S2. Green metrics for the second step of the CF reaction of **4a** with Cs_2CO_3.

Synthesis method	Substrates [g]	Solvents, additives [g]	Product [g]	Other waste [g]	PMI	E-factor
Solvent	0.96	4.40	0.20	0.76	**26.4**	**25.4**
Mechanochemistry	0.31	0.90	0.07	0.242	**17.3**	**16.3**

3. Literature

[1] Vogt, H.; Frauendorf, C.; Fischer, A.; Jones, P. G. Synthese Und Kristallstruktur von Tribrommethyltriphenylphosphonium-Bromid Und Bis(Tribrommethyltriphenylphosphonium)-Tribroniid-Bromid / Synthesis and Crystal Structure of Tribromomethyltriphenylphosphonium Bromide and Bis(Tribromomethyltriphenylphosphonium) Tribromide Bromide. *Z. für Naturforsch. B* **1995**, *50* (2), 223–228.

[2] Salehi, P.; Tanbakouchian, Z.; Farajinia-Lehia, N.; Shiri, M. Cascade synthesis of 2,4-disulfonylpyrroles by the sulfonylation/[2 + 3]-cycloaddition reactions of gem-dibromoalkenes with arylsulfonyl methyl isocyanides. *RSC Adv.* **2021**, *11*, 13292–13296.

4. ^{1}H, $^{13}C\{^{1}H\}$, $^{31}P\{^{1}H\}$ NMR, MS (EI) and FT-IR spectra

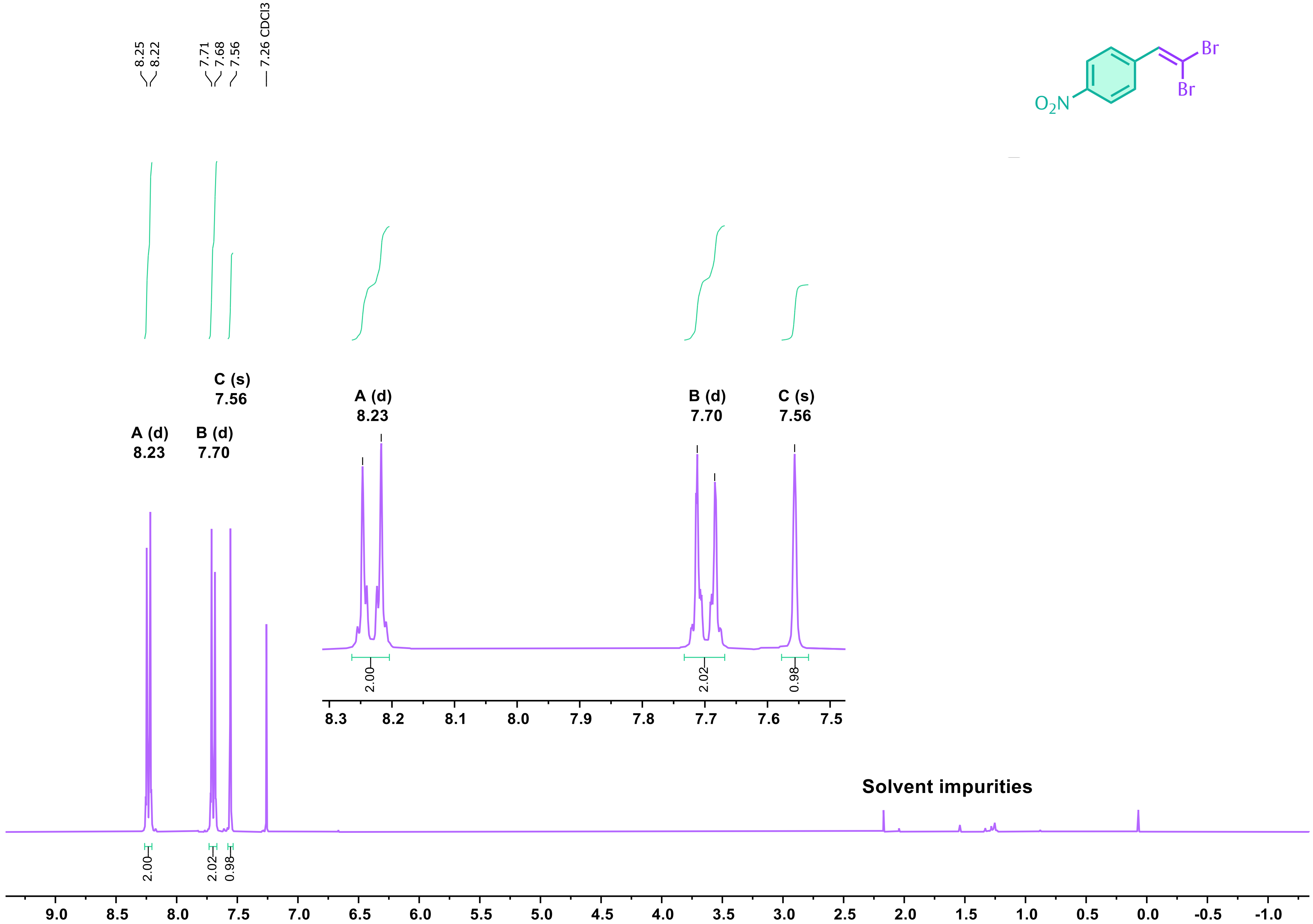

Figure S9. ^{1}H NMR spectrum (300 MHz, $CDCl_3$) of compound **4a**.

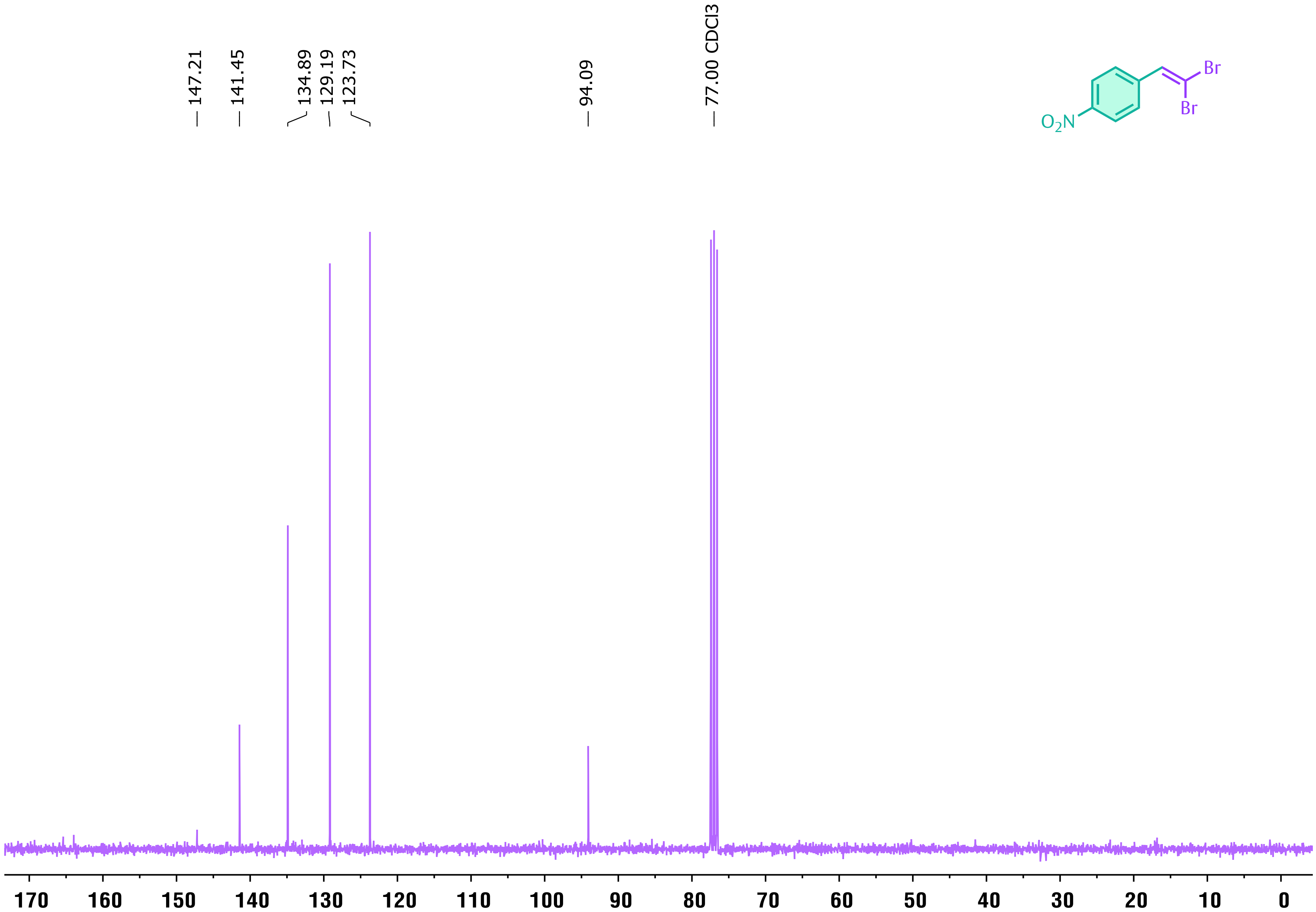

Figure S10. $^{13}C\{^1H\}$ NMR spectrum (101 MHz, $CDCl_3$) of compound **4a**.

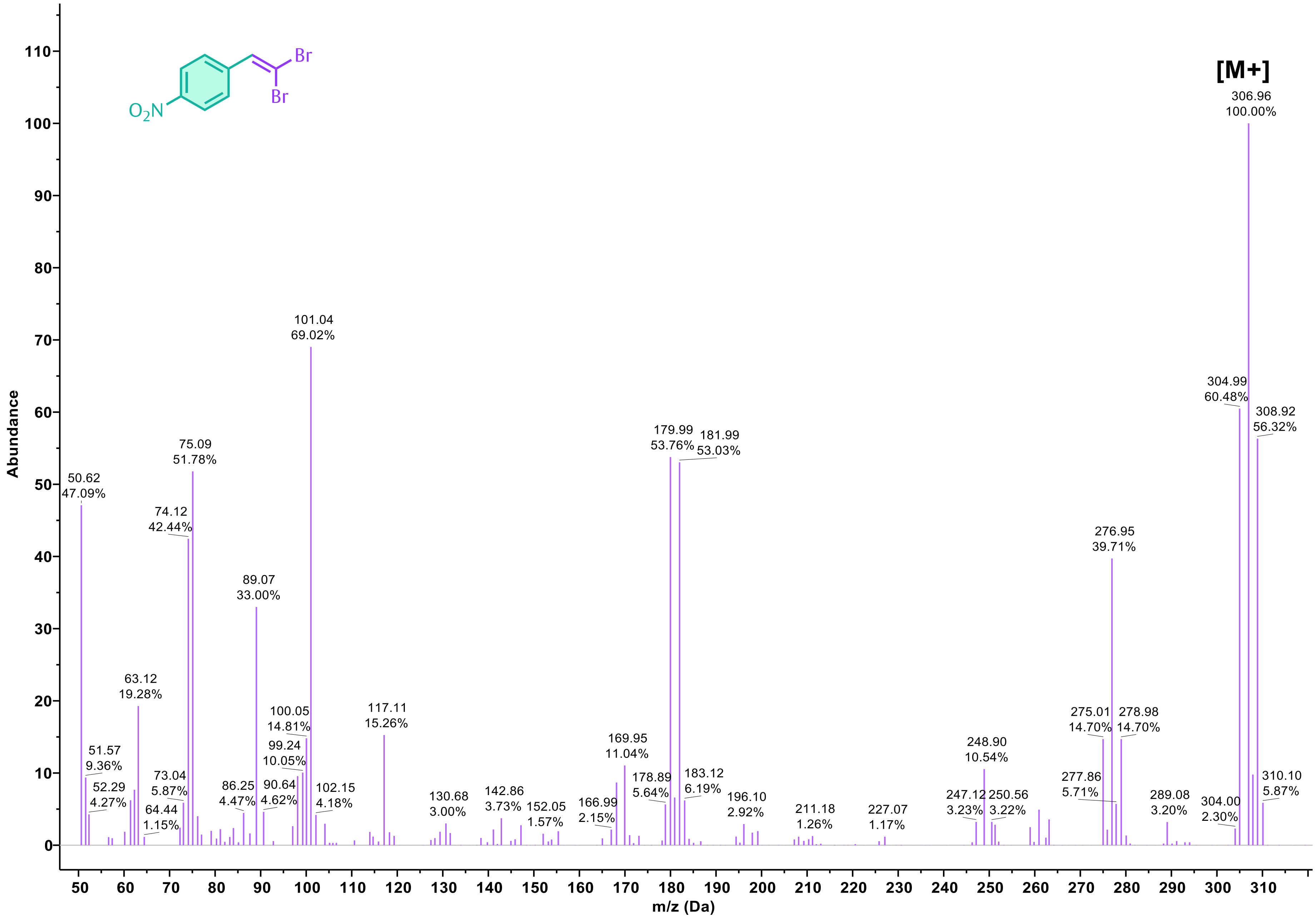

Figure S11. Mass spectrum of compound **4a**.

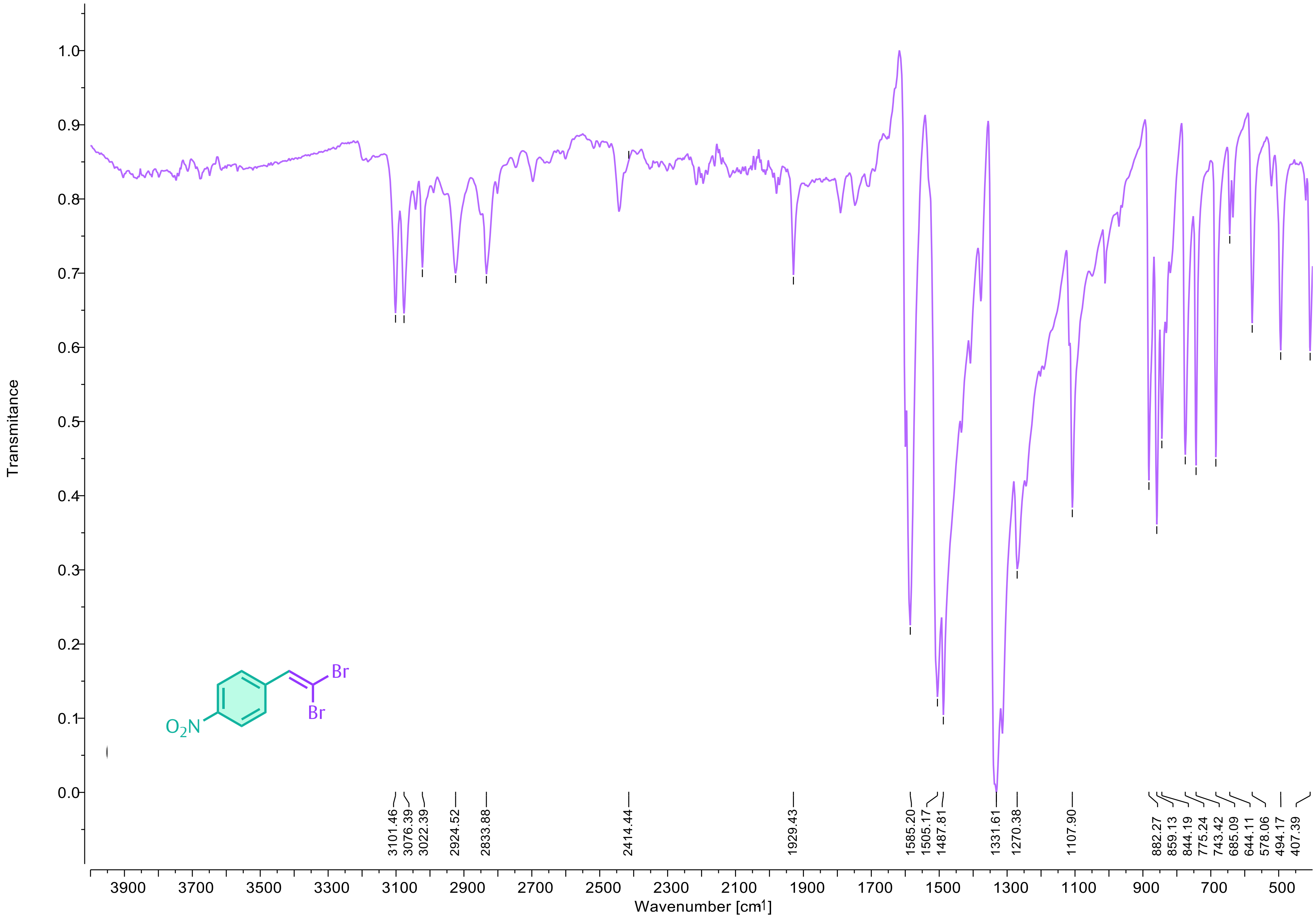

Figure S12. FT-IR spectrum of compound **4a**.

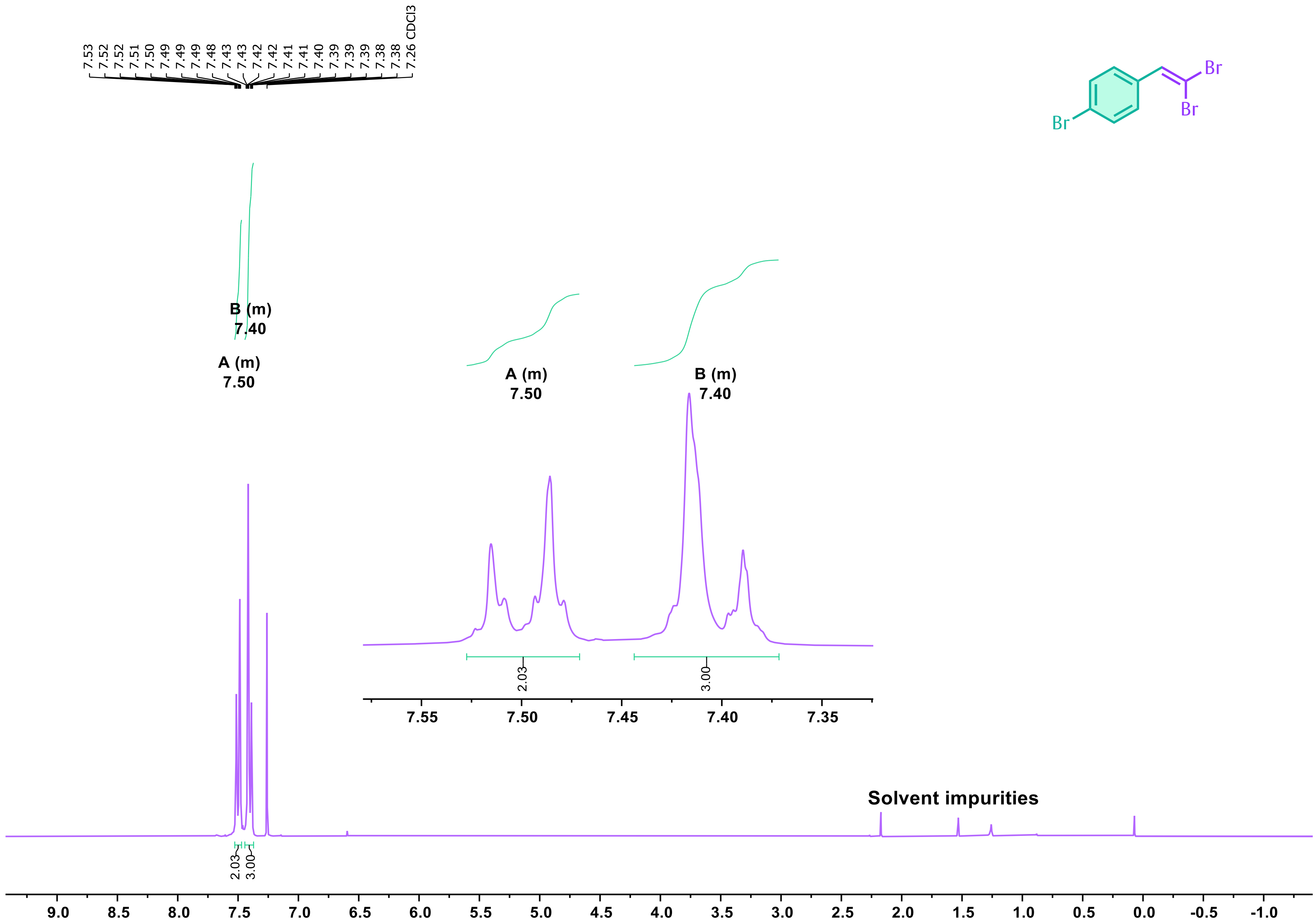

Figure S13. ^{1}H NMR spectrum (300 MHz, $CDCl_3$) of compound **4b**.

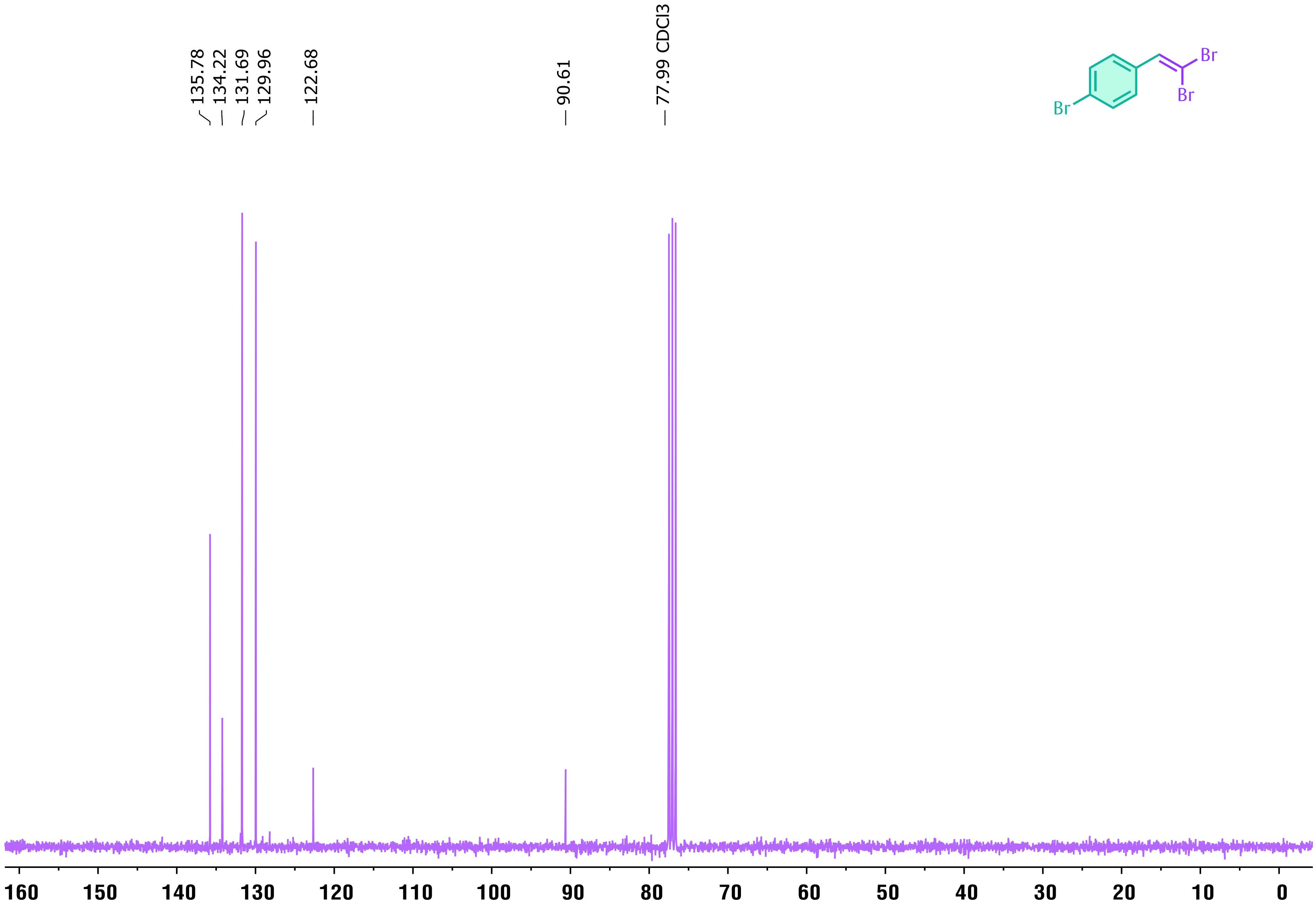

Figure S14. $^{13}C\{^{1}H\}$ NMR spectrum (101 MHz, $CDCl_3$) of compound **4b**.

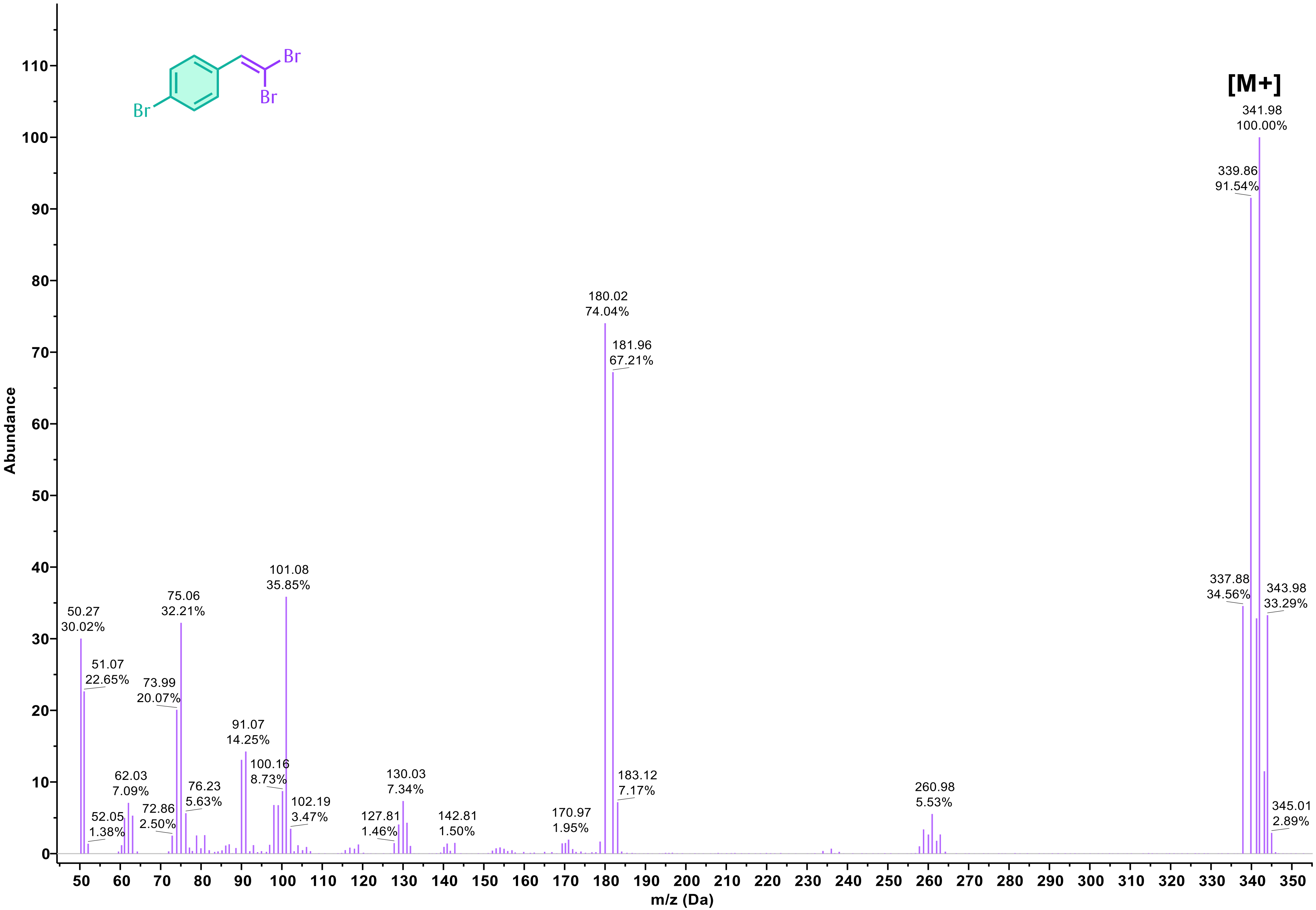

Figure S15. Mass spectrum of compound **4b**.

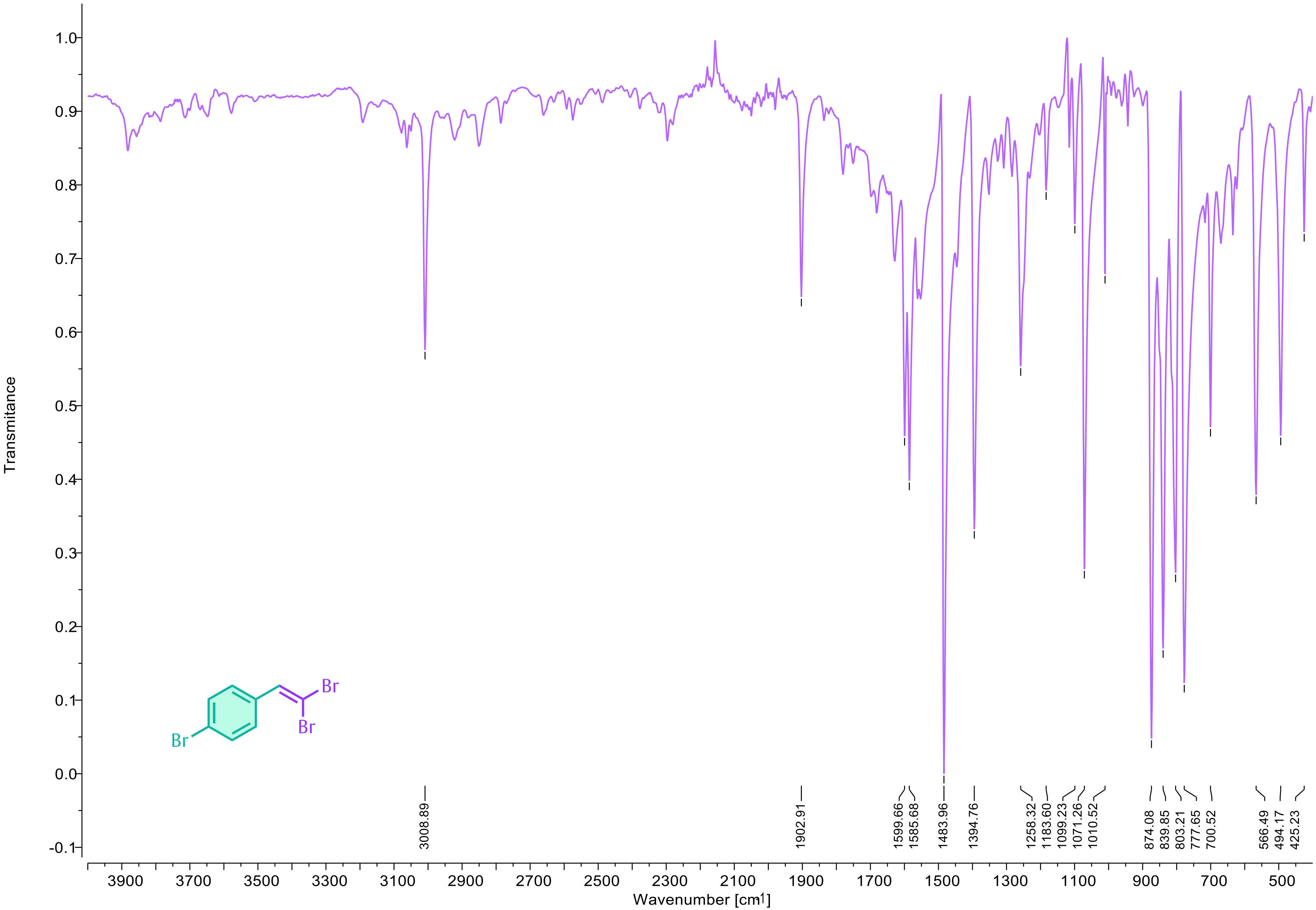

Figure S16. FT-IR spectrum of compound **4b**.

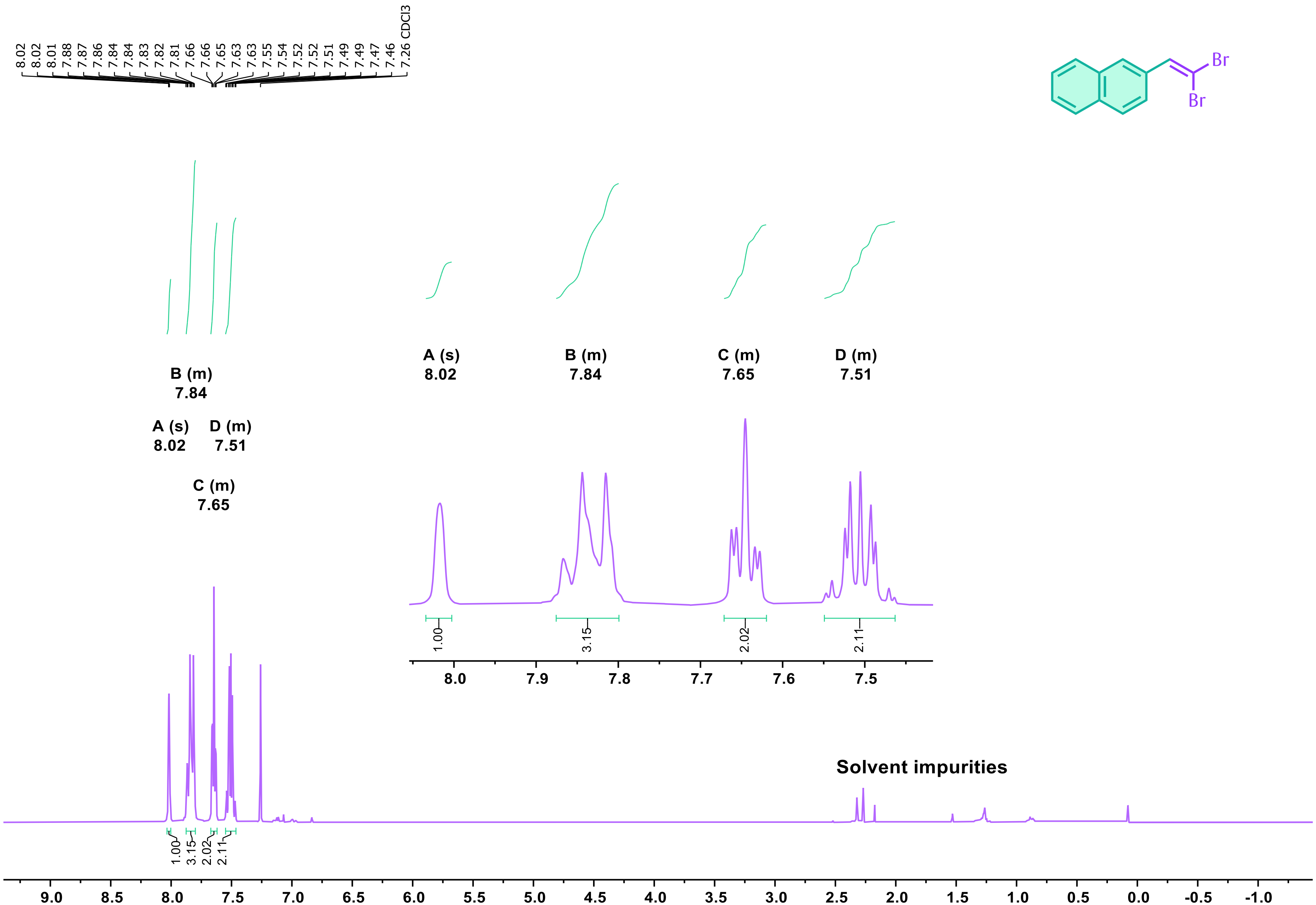

Figure S17. ^{1}H NMR spectrum (300 MHz, $CDCl_3$) of compound **4c**.

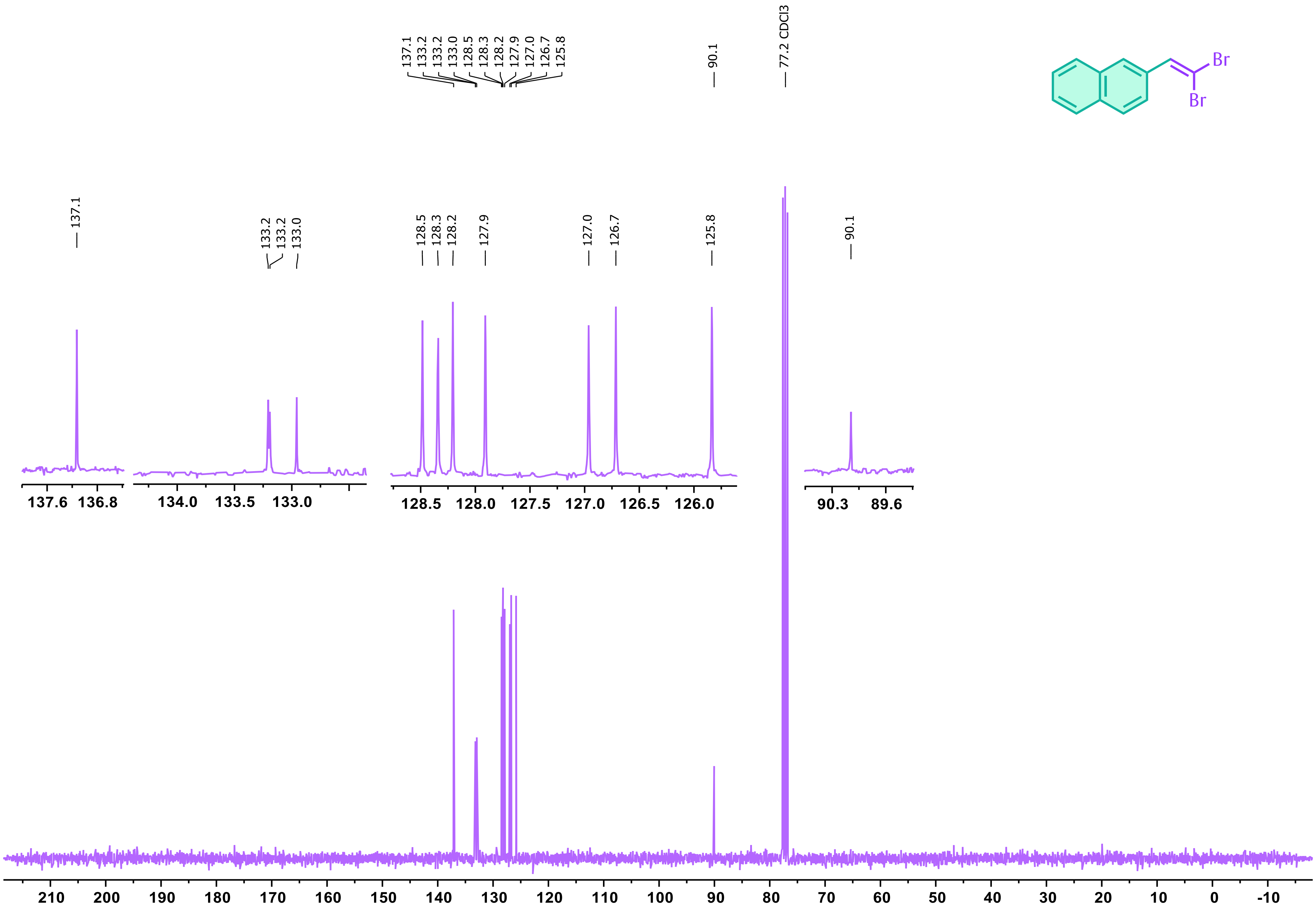

Figure S18. $^{13}C\{^{1}H\}$ NMR spectrum (101 MHz, $CDCl_3$) of compound **4c**.

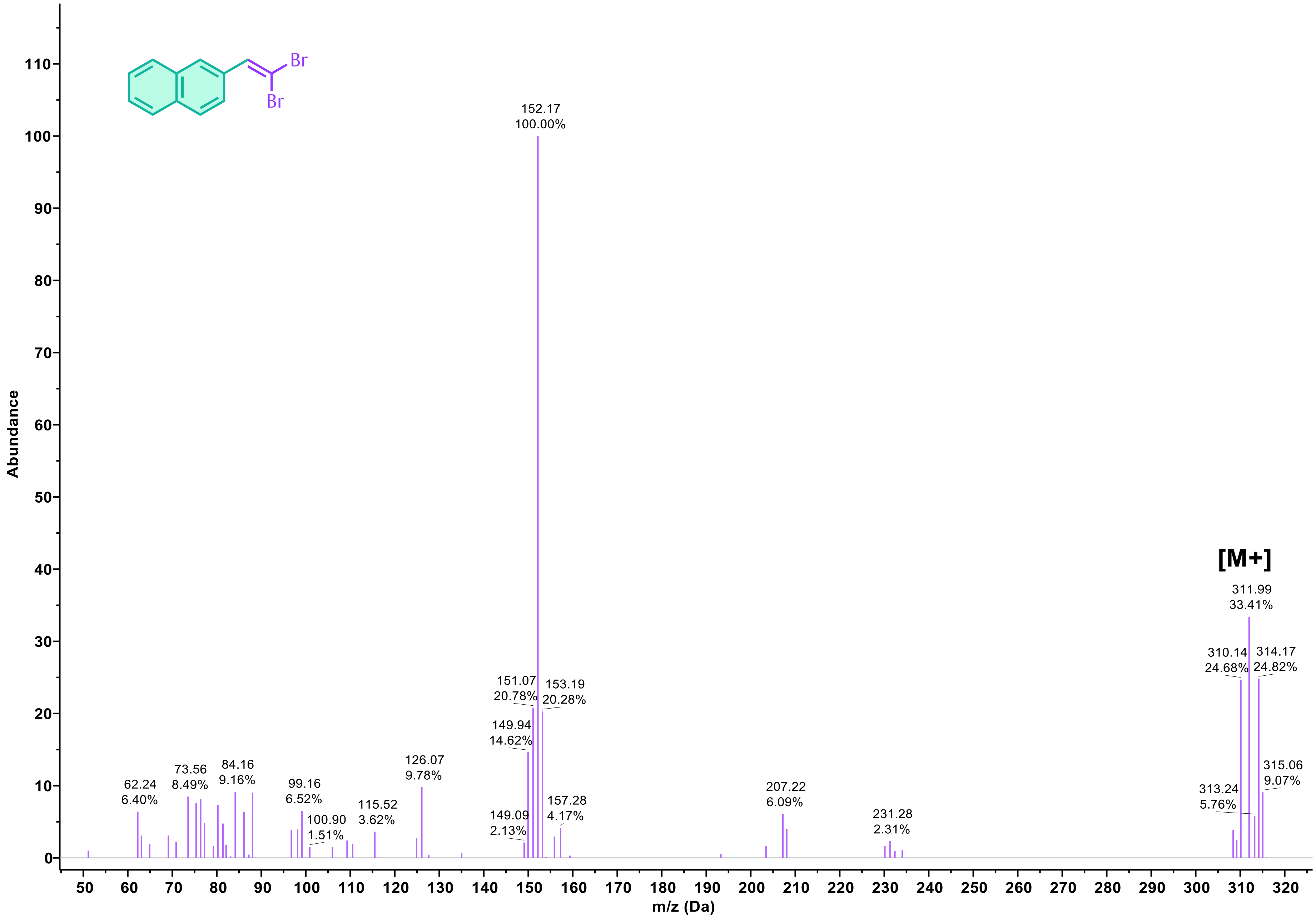

Figure S19. Mass spectrum of compound **4c**.

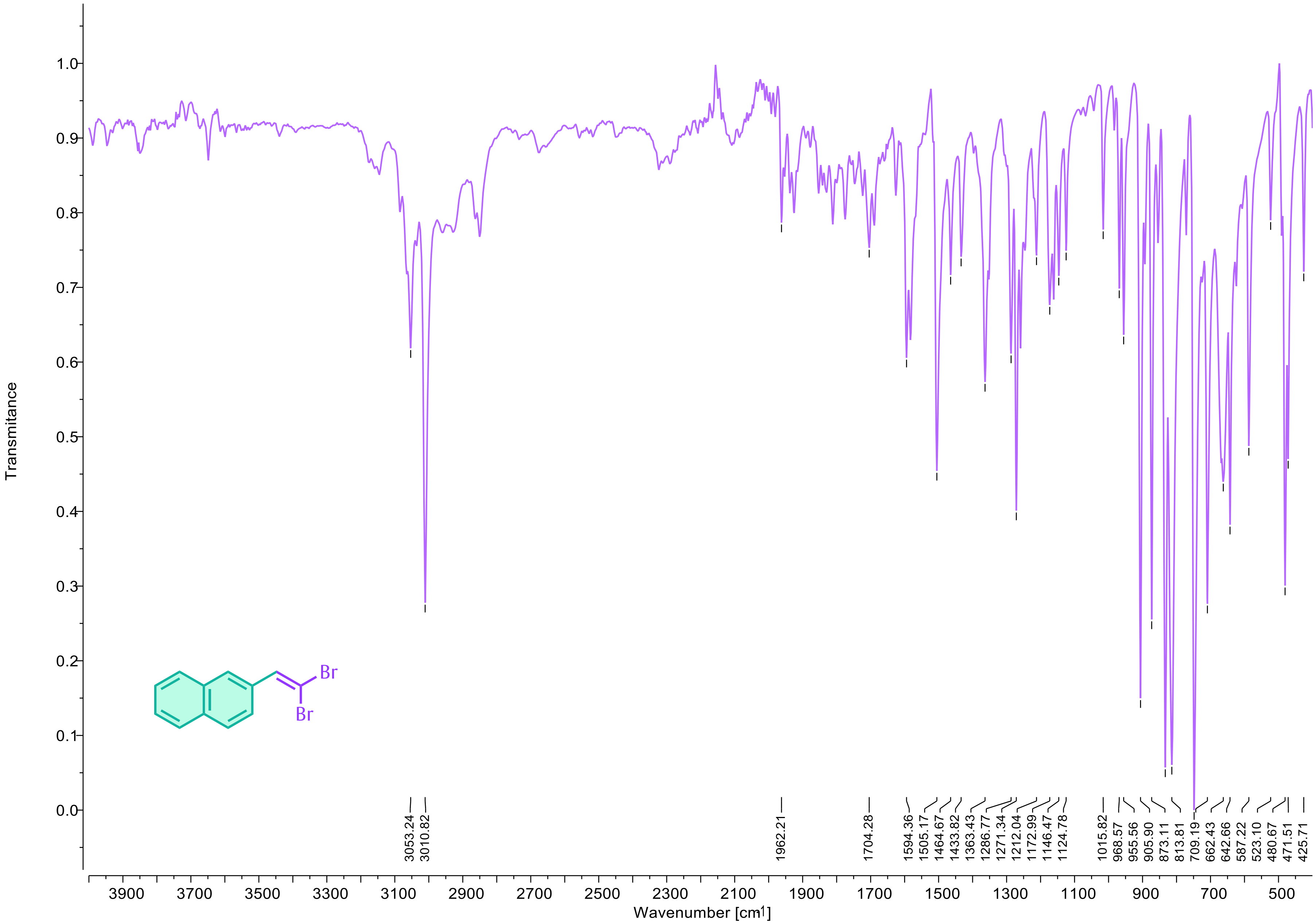

Figure S20. FT-IR spectrum of compound **4c**.

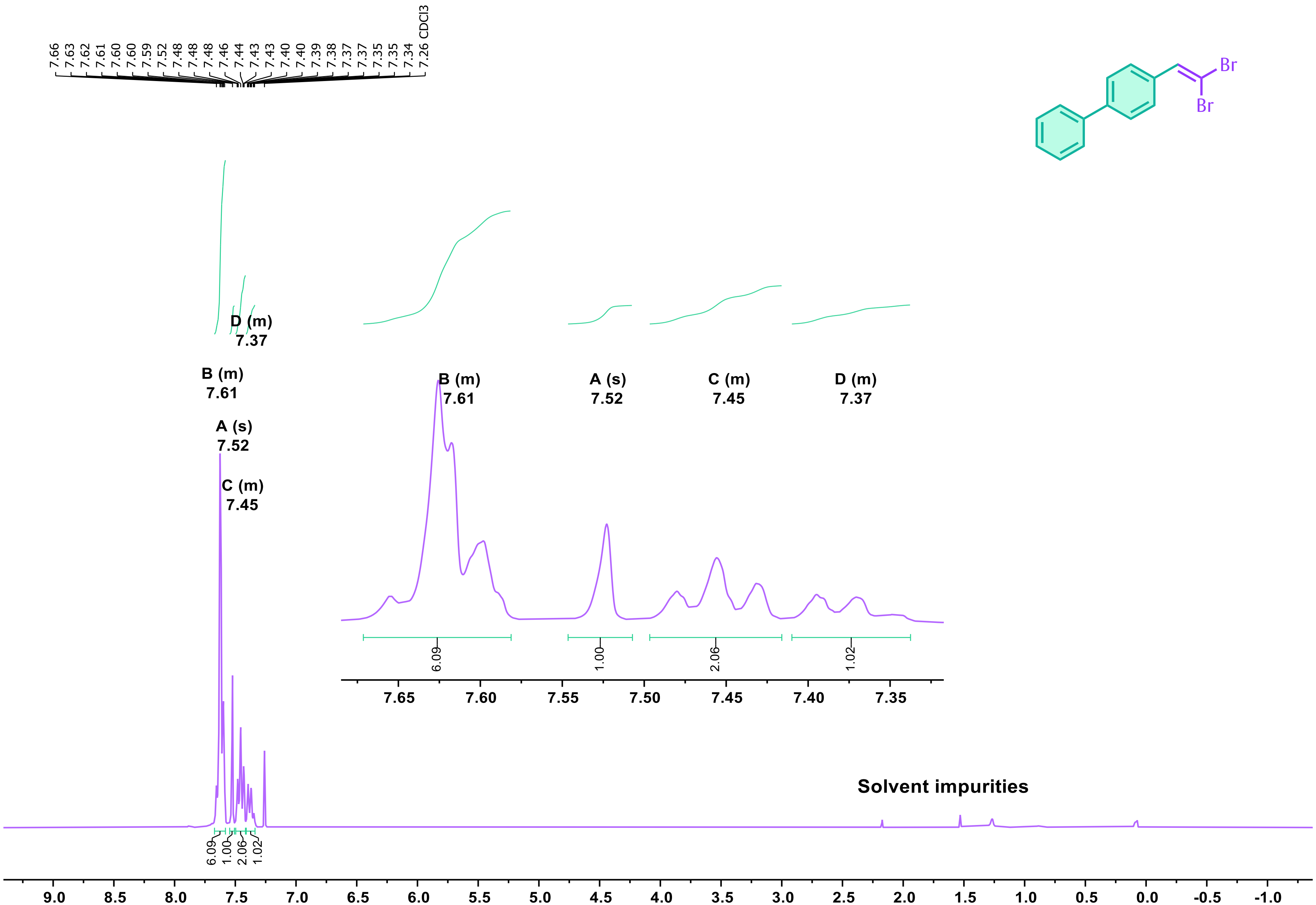

Figure S21. ^{1}H NMR spectrum (300 MHz, $CDCl_3$) of compound **4d**.

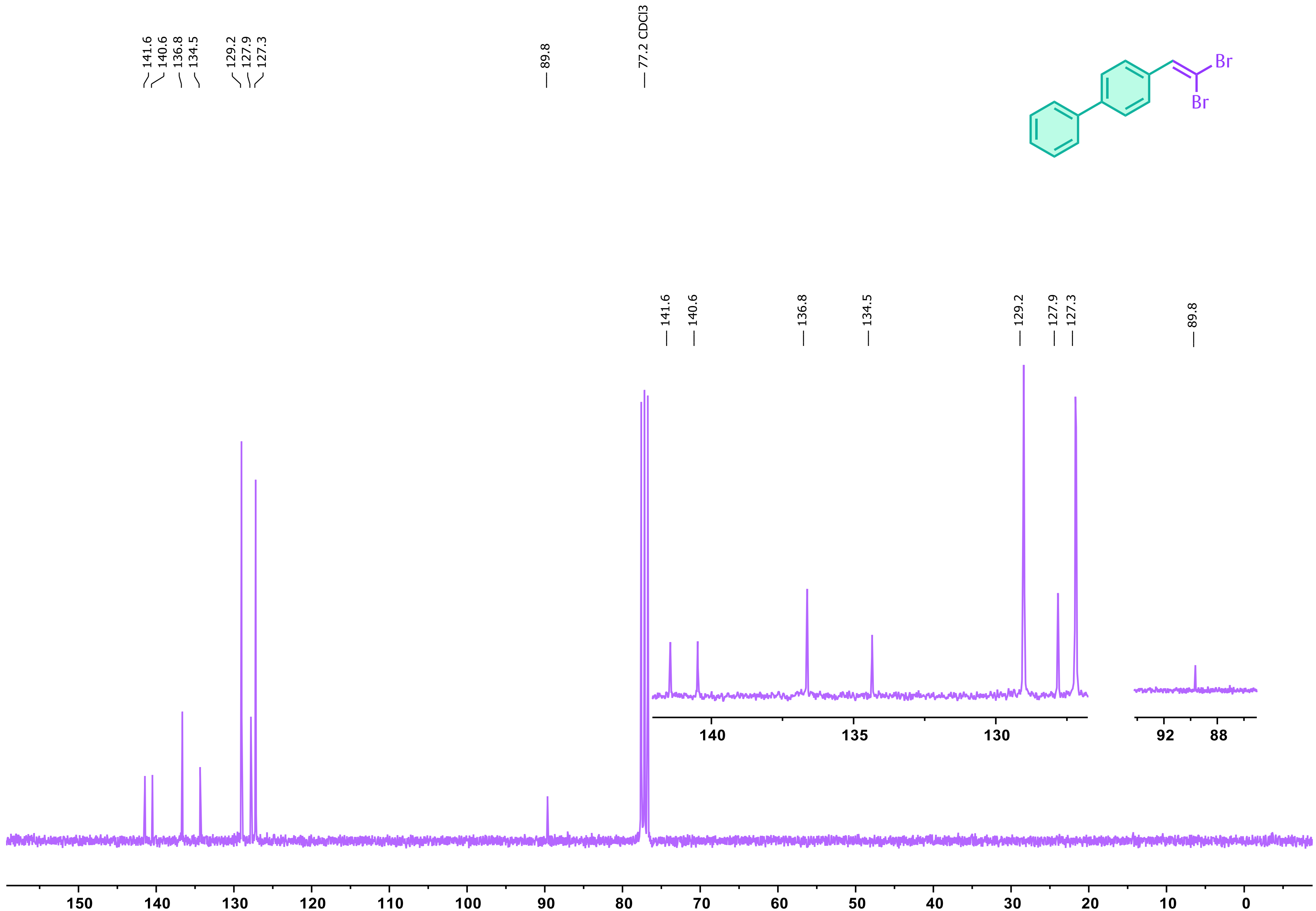

Figure S22. $^{13}C\{^{1}H\}$ NMR spectrum (101 MHz, $CDCl_3$) of compound **4d**.

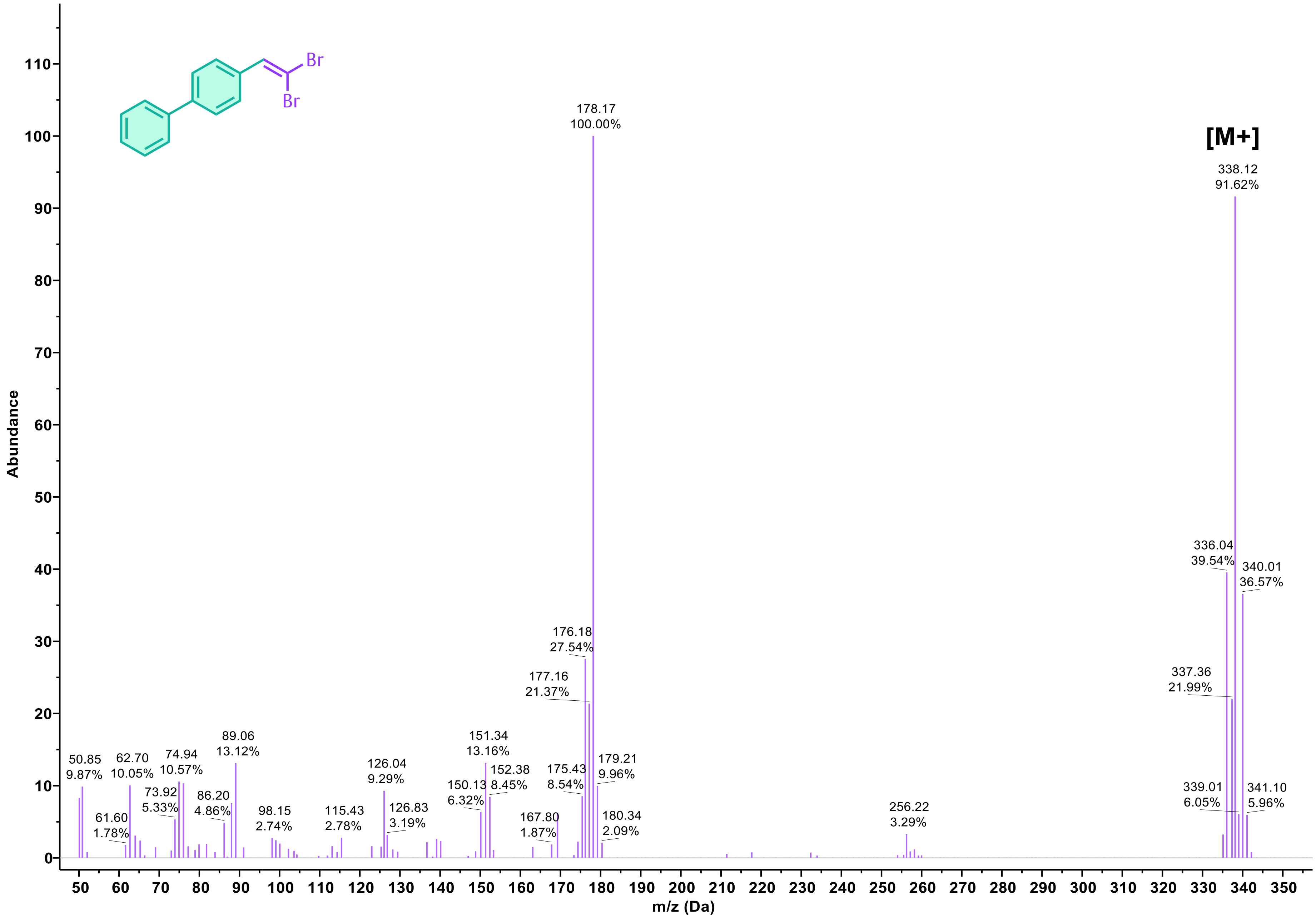

Figure S23. Mass spectrum of compound **4d**.

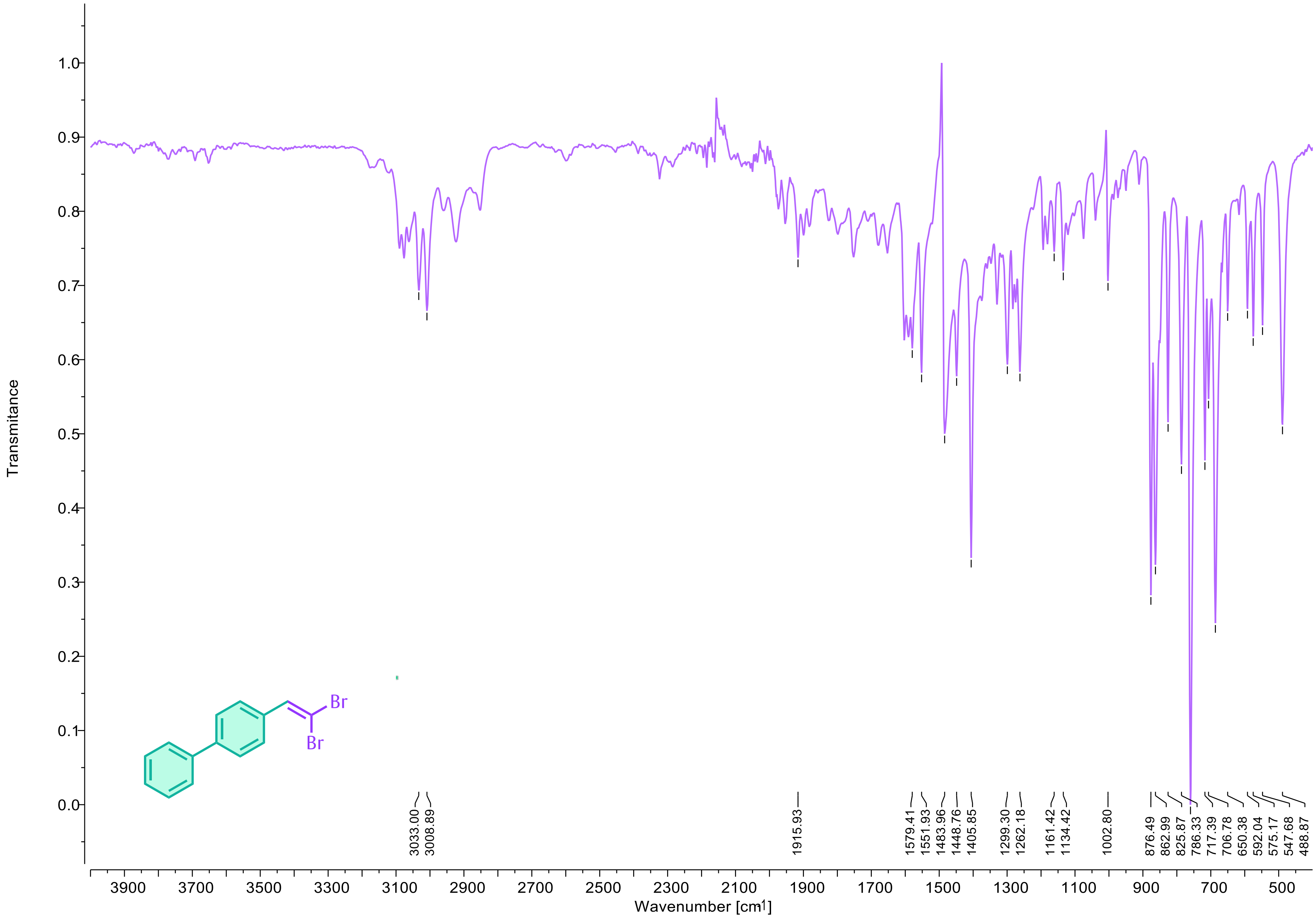

Figure S24. FT-IR spectrum of compound **4d**.

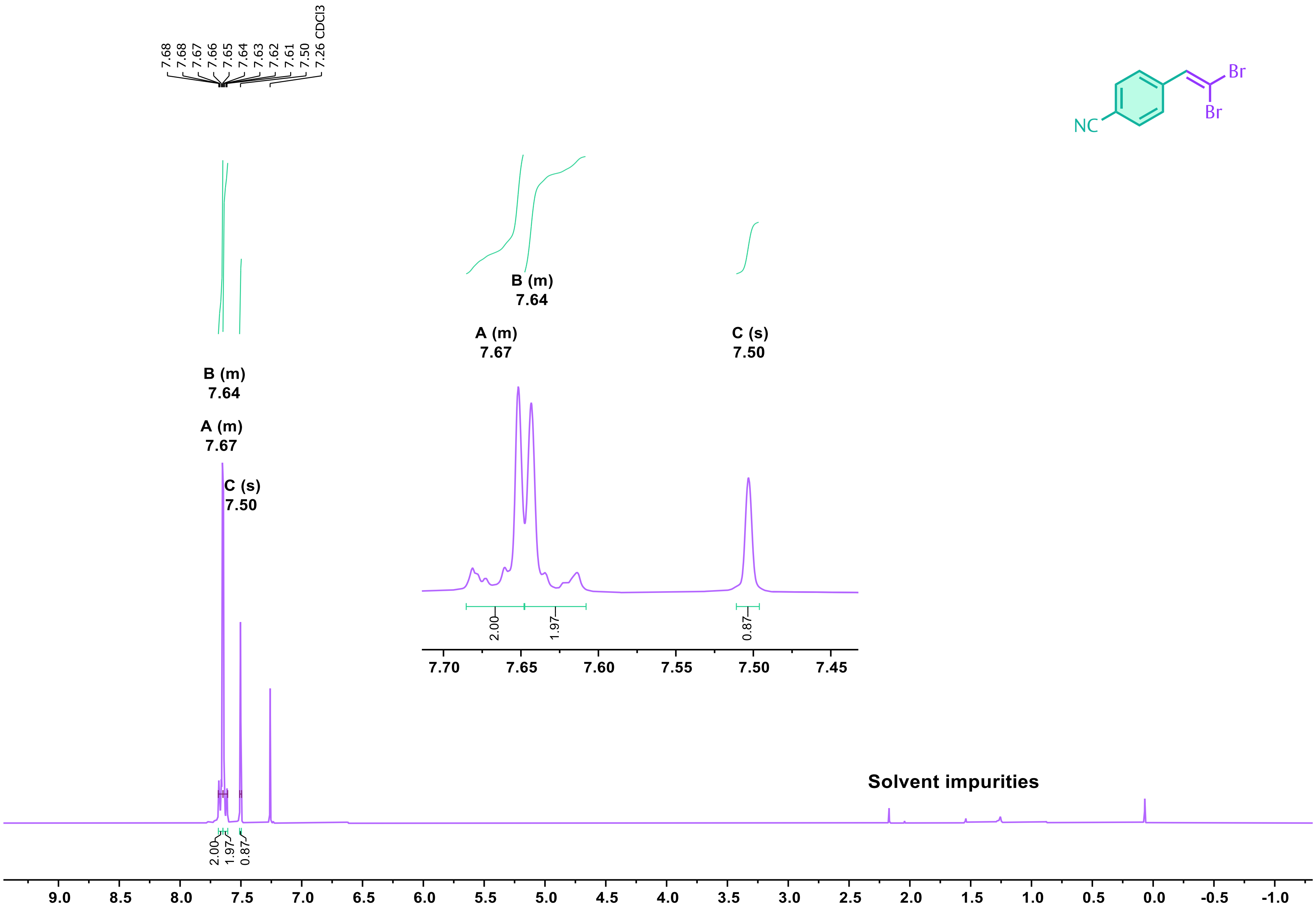

Figure S25. ^{1}H NMR spectrum (300 MHz, $CDCl_3$) of compound **4e**.

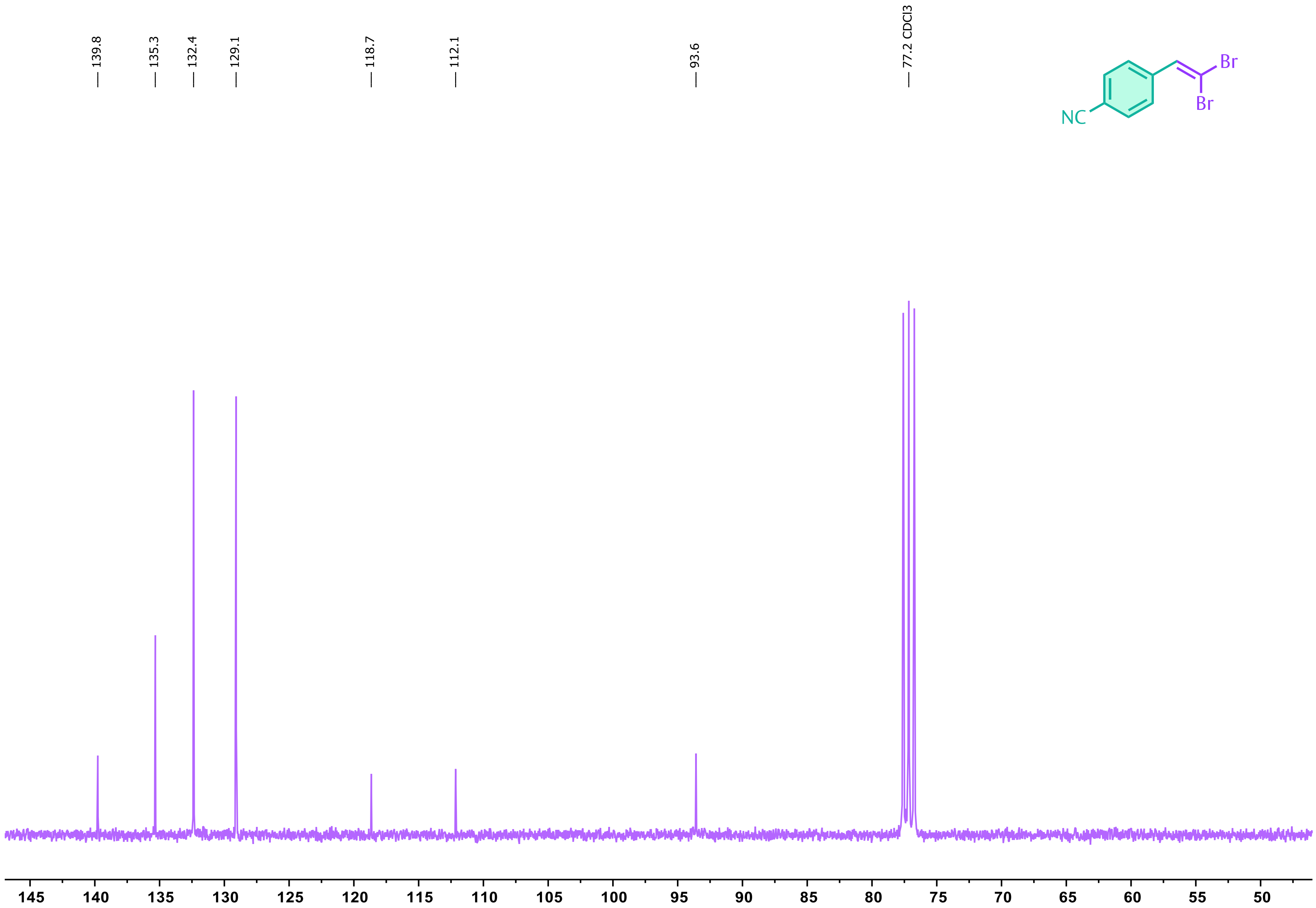

Figure S26. $^{13}C\{^1H\}$ NMR spectrum (101 MHz, $CDCl_3$) of compound **4e**.

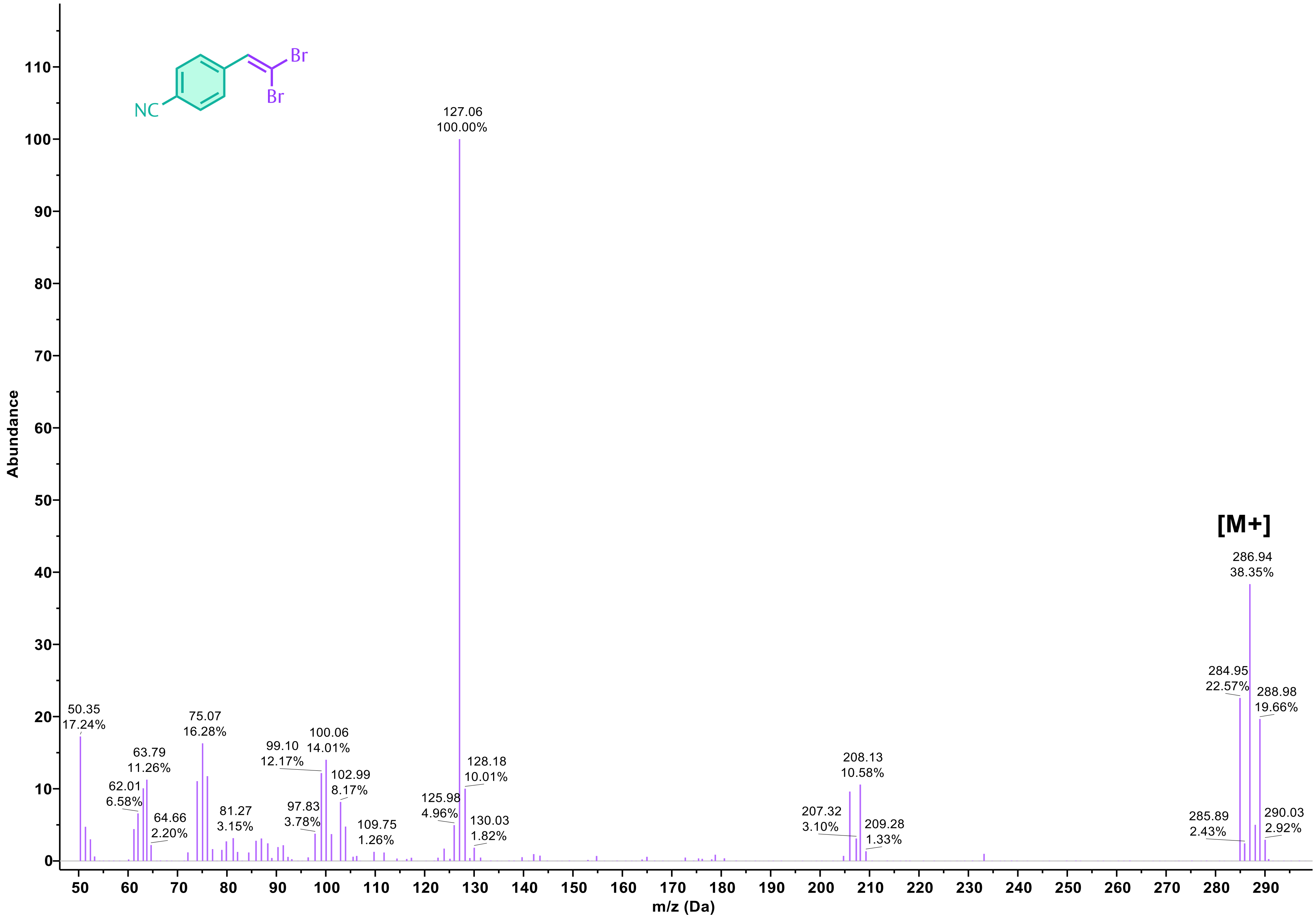

Figure S27. Mass spectrum of compound **4e**.

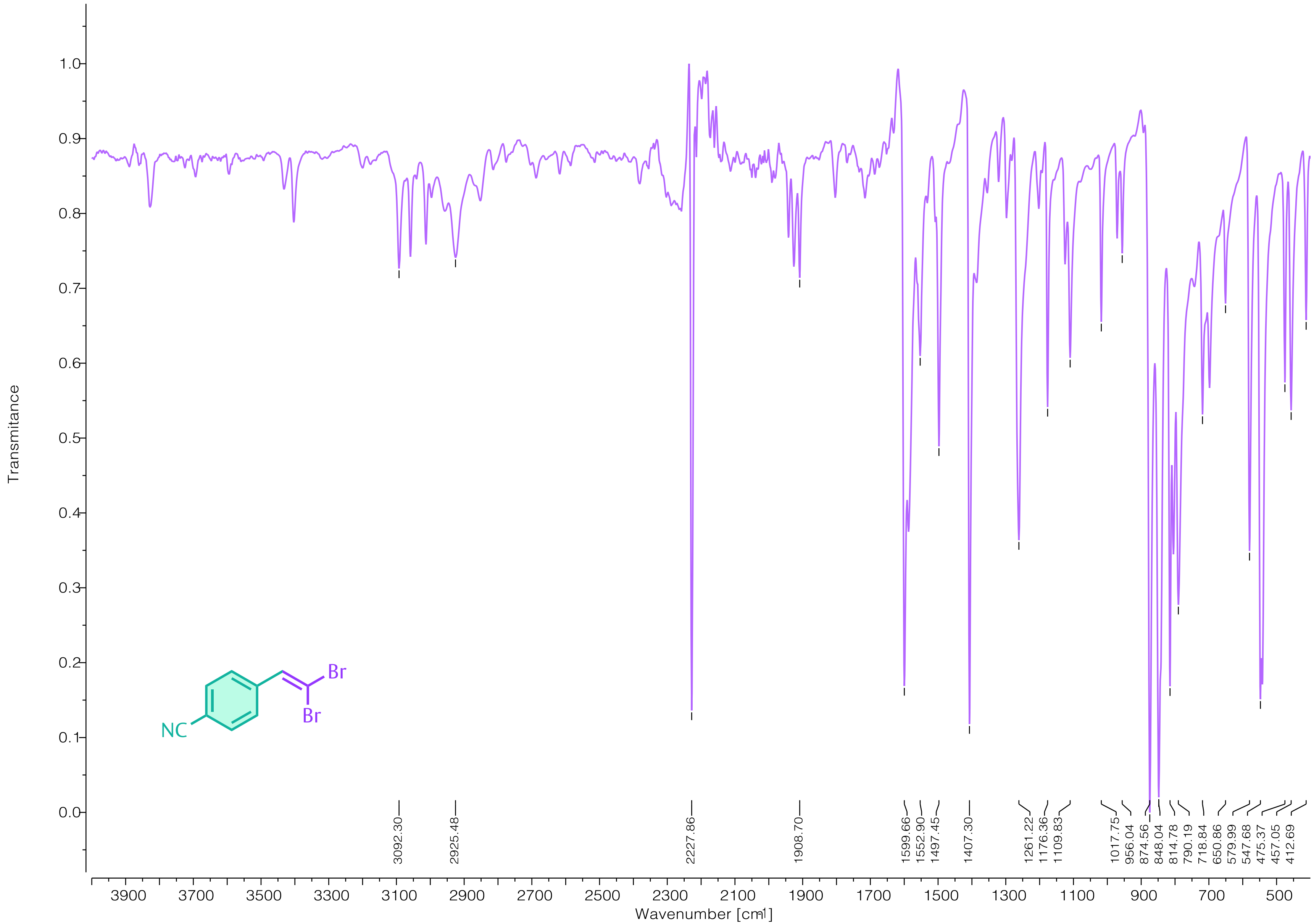

Figure S28. FT-IR spectrum of compound **4e**.

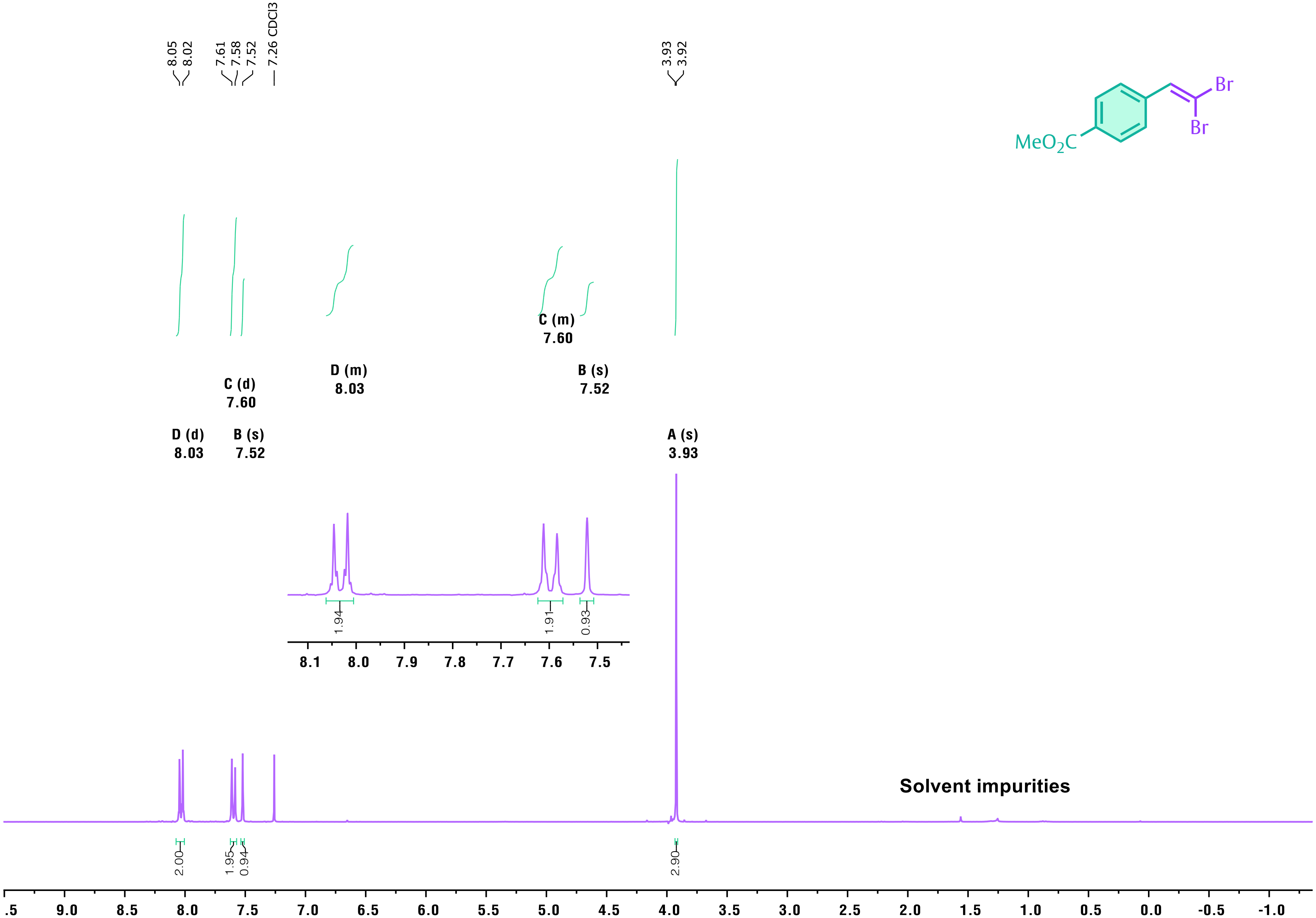

Figure S29. ^{1}H NMR spectrum (300 MHz, $CDCl_3$) of compound **4f**.

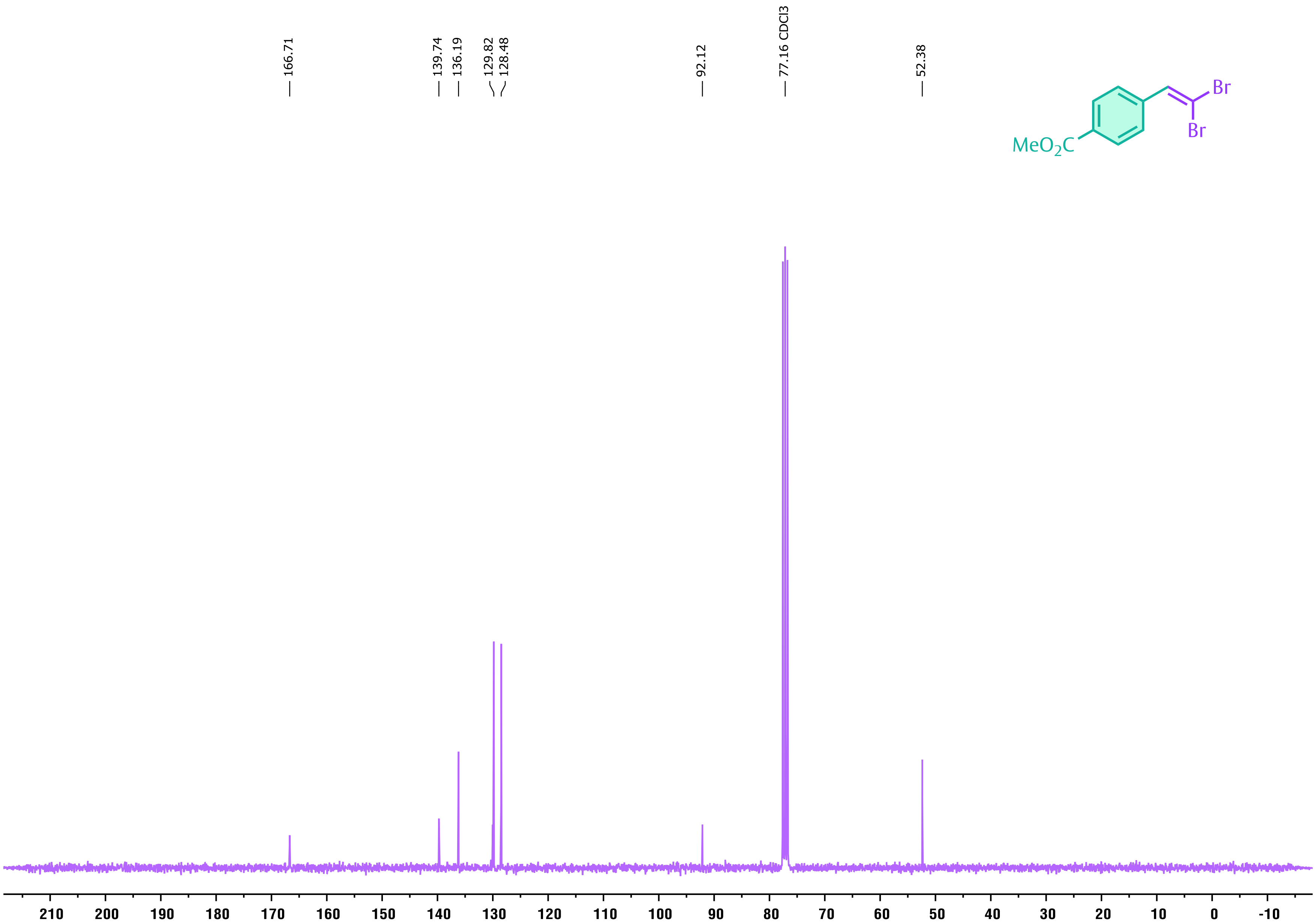

Figure S30. $^{13}C\{^{1}H\}$ NMR spectrum (101 MHz, $CDCl_3$) of compound **4f**.

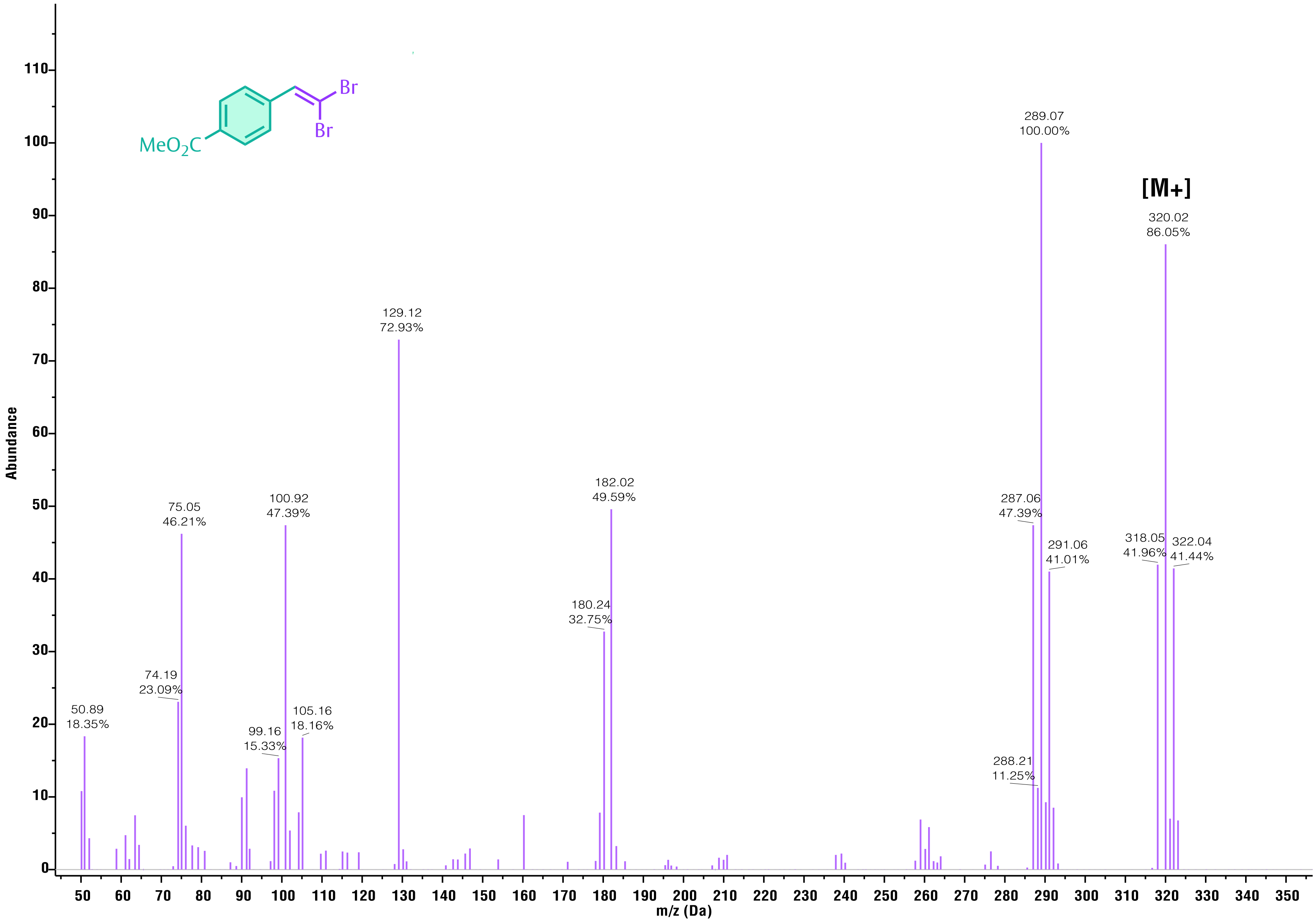

Figure S31. Mass spectrum of compound **4f**.

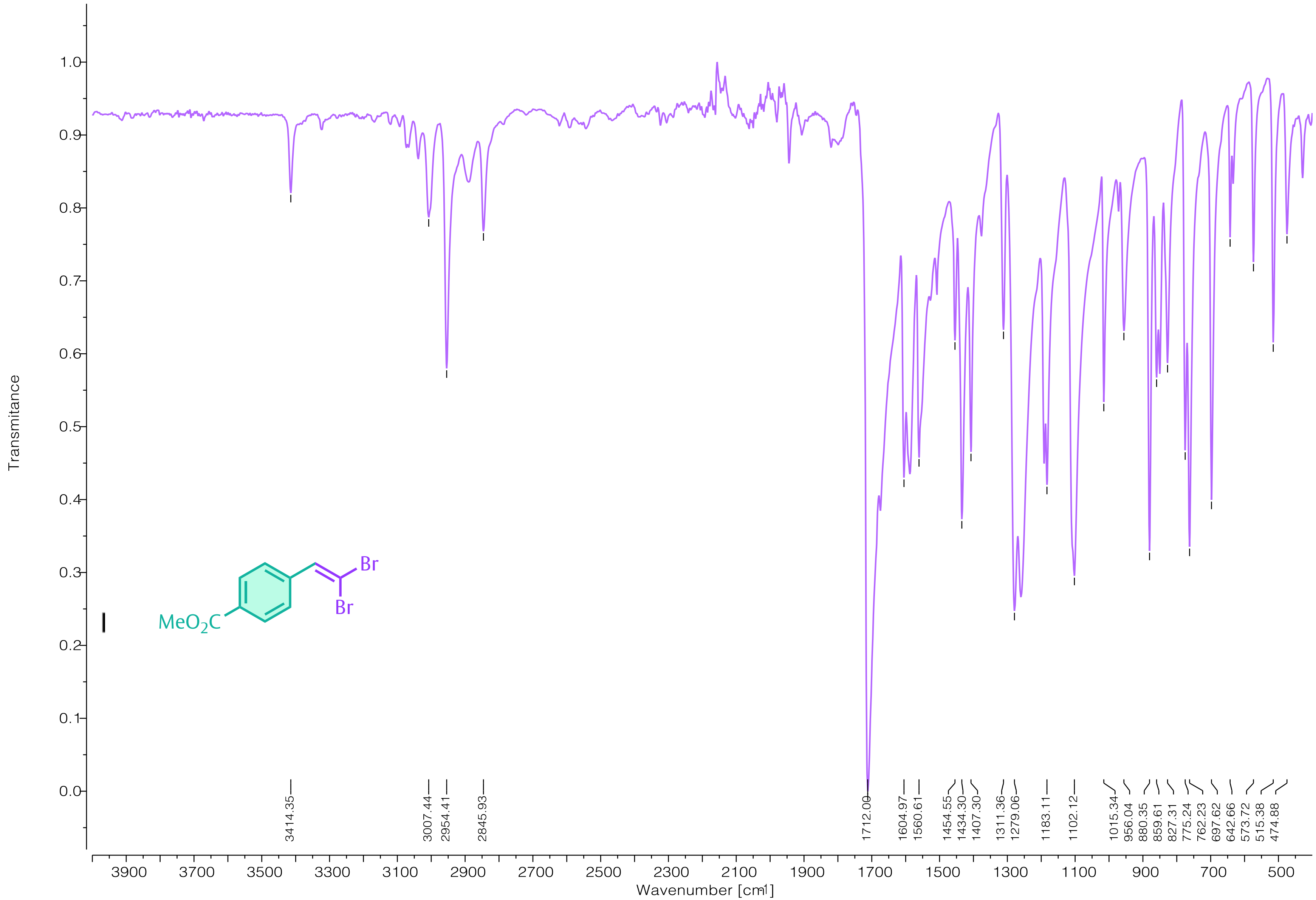

Figure S32. FT-IR spectrum of compound **4f**.

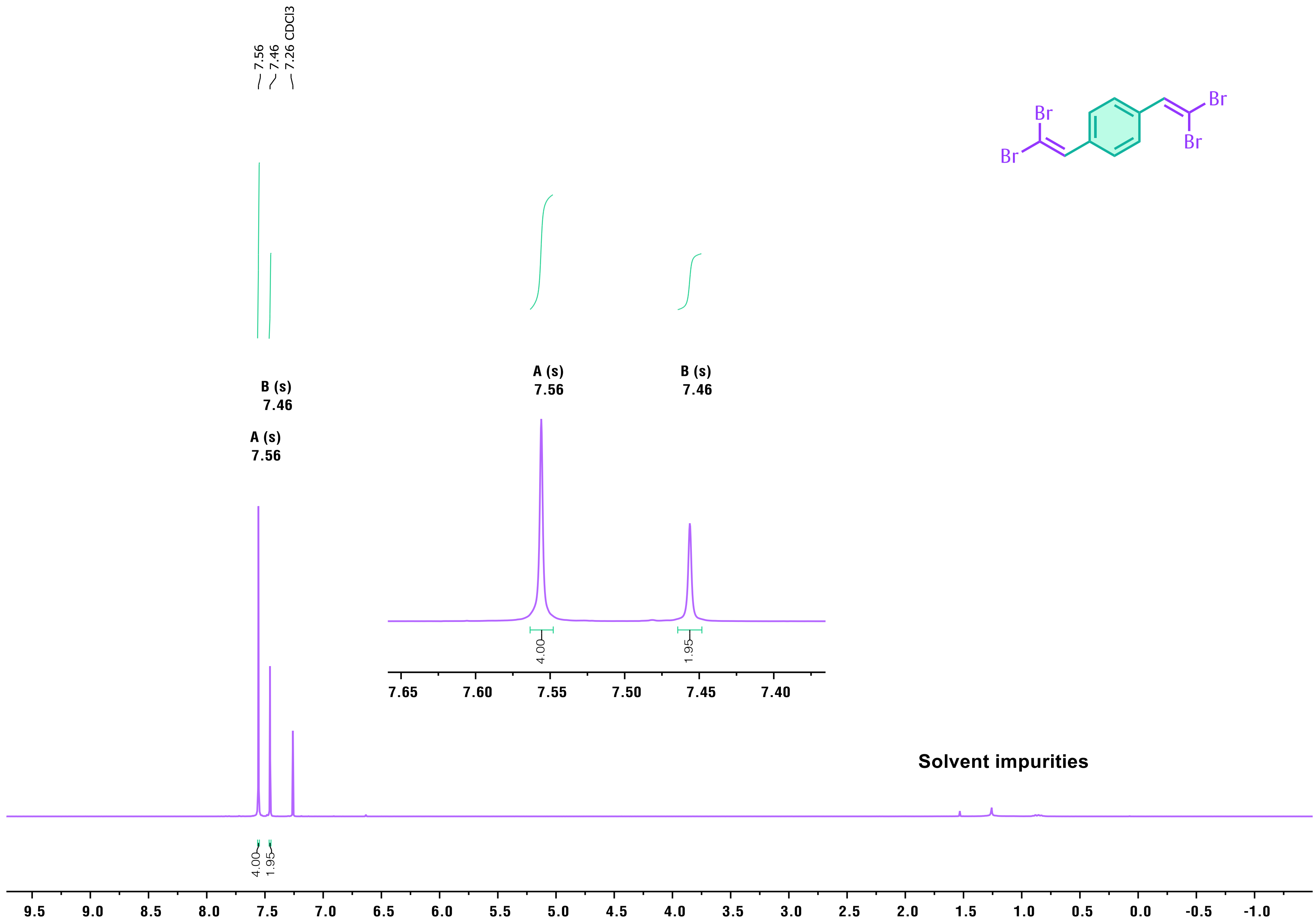

Figure S33. ^{1}H NMR spectrum (300 MHz, $CDCl_3$) of compound **4g**.

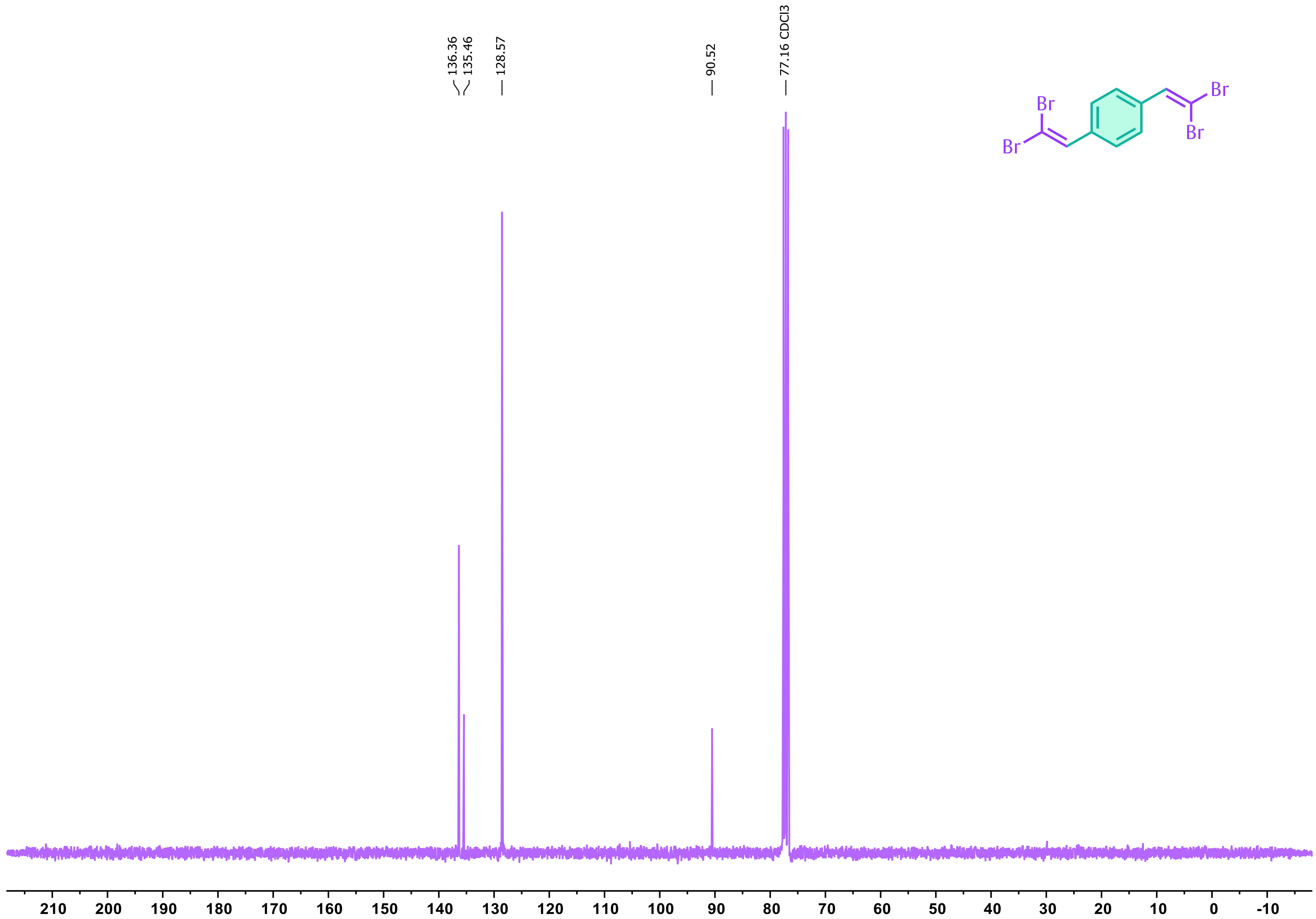

Figure S34. $^{13}C\{^{1}H\}$ NMR spectrum (101 MHz, $CDCl_3$) of compound **4g**.

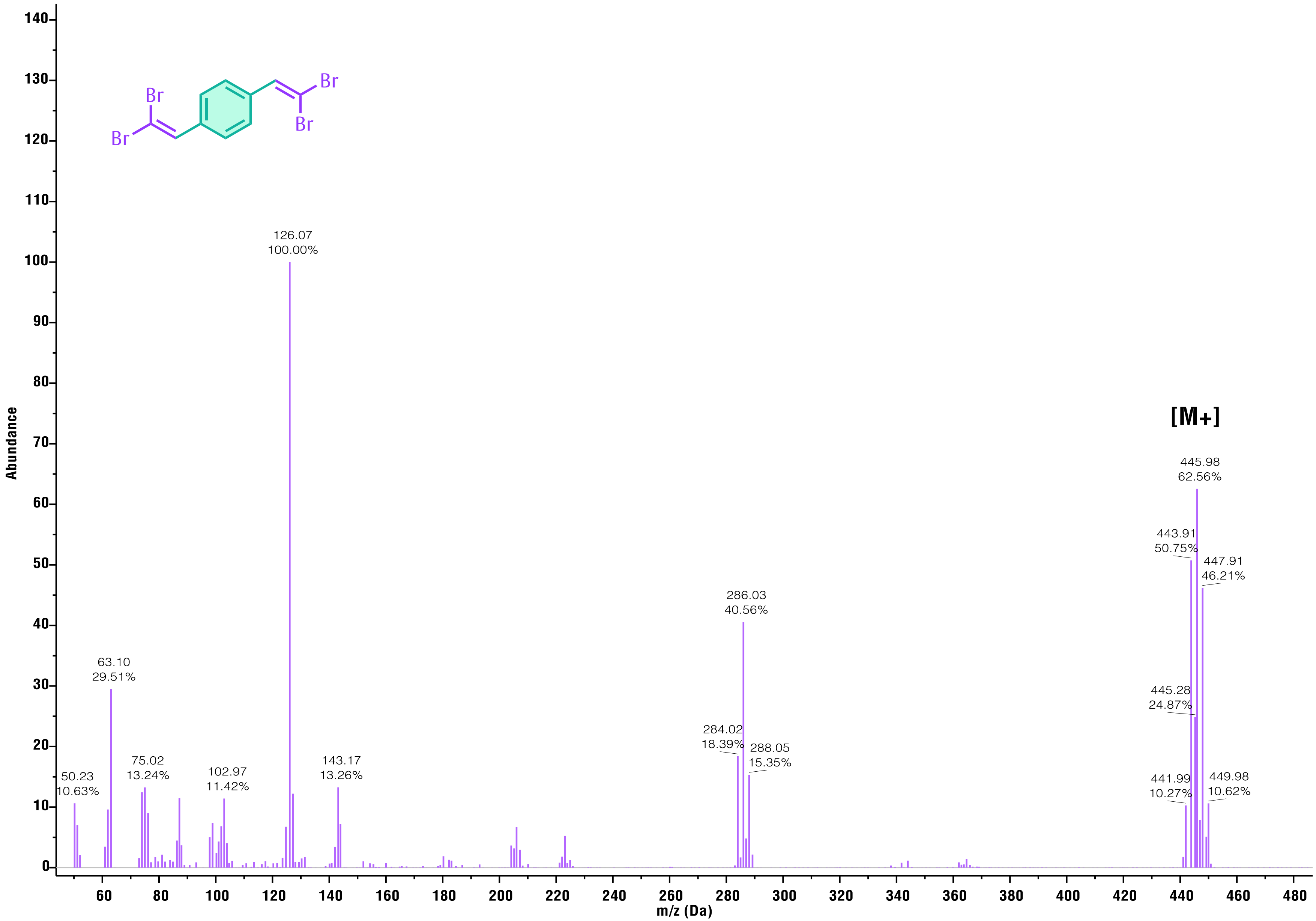

Figure S35. Mass spectrum of compound **4g**.

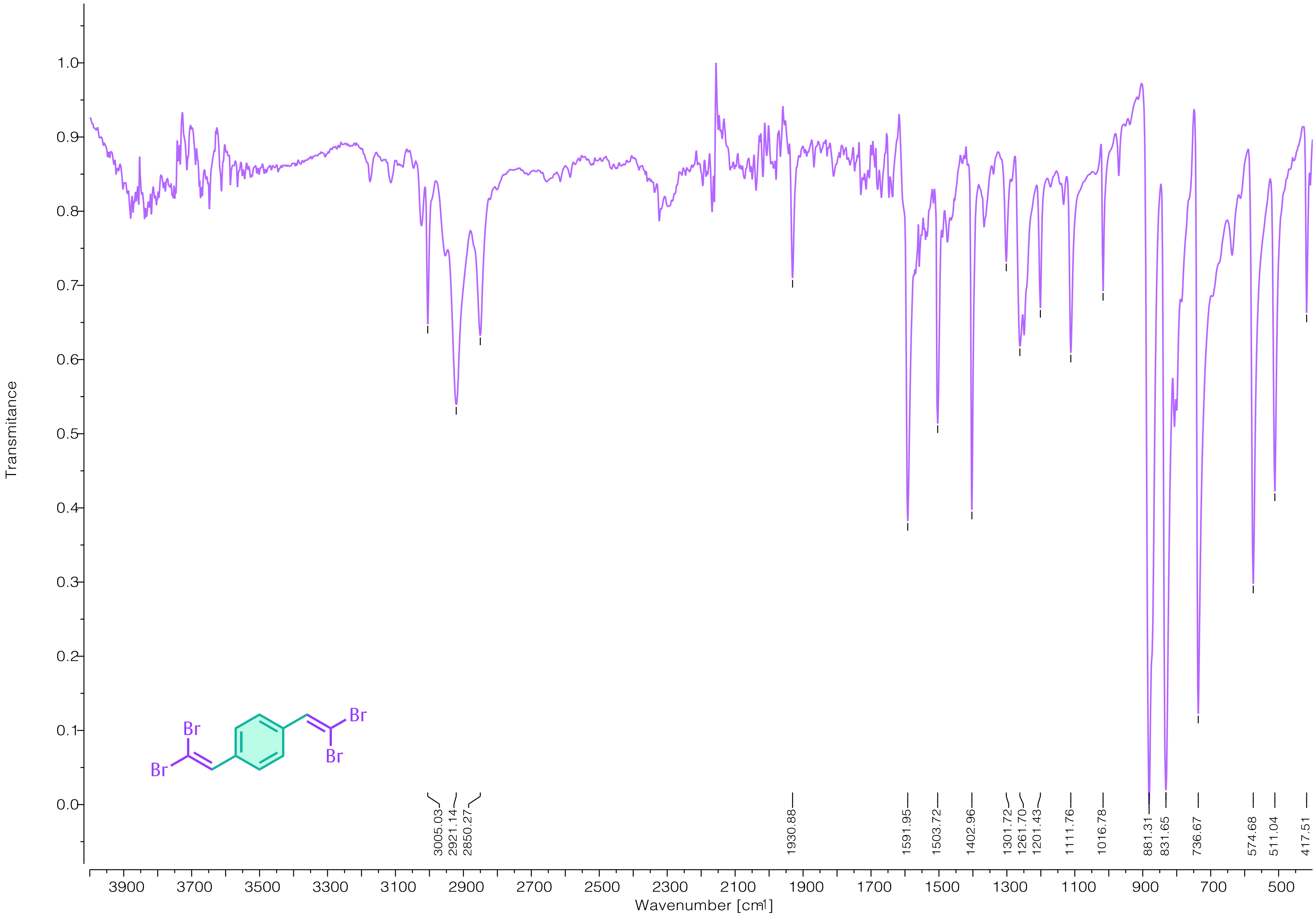

Figure S36. FT-IR spectrum of compound **4g**.

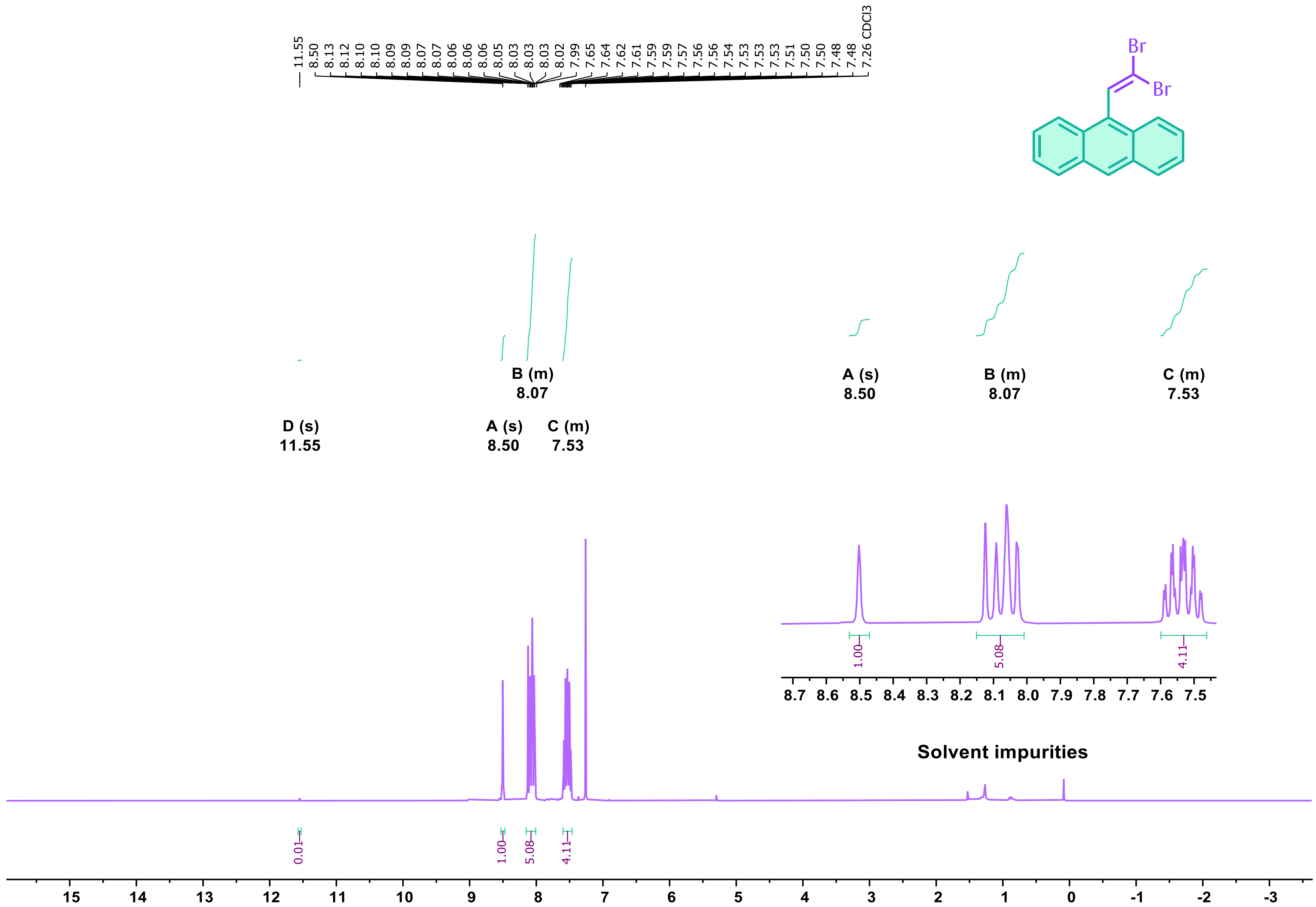

Figure S37. ^{1}H NMR spectrum (300 MHz, $CDCl_3$) of compound **4h**.

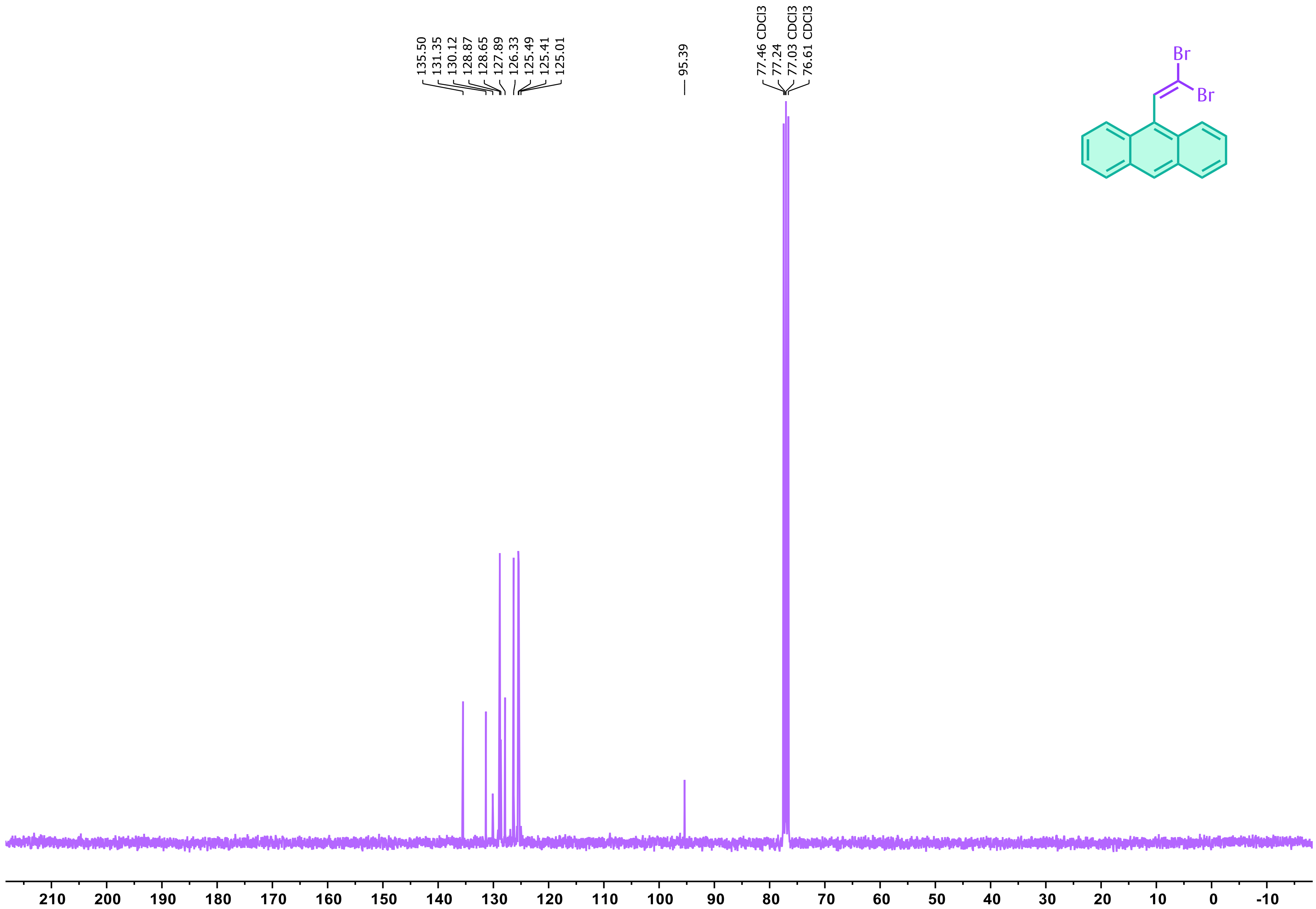

Figure S38. $^{13}C\{^1H\}$ NMR spectrum (101 MHz, $CDCl_3$) of compound **4h**.

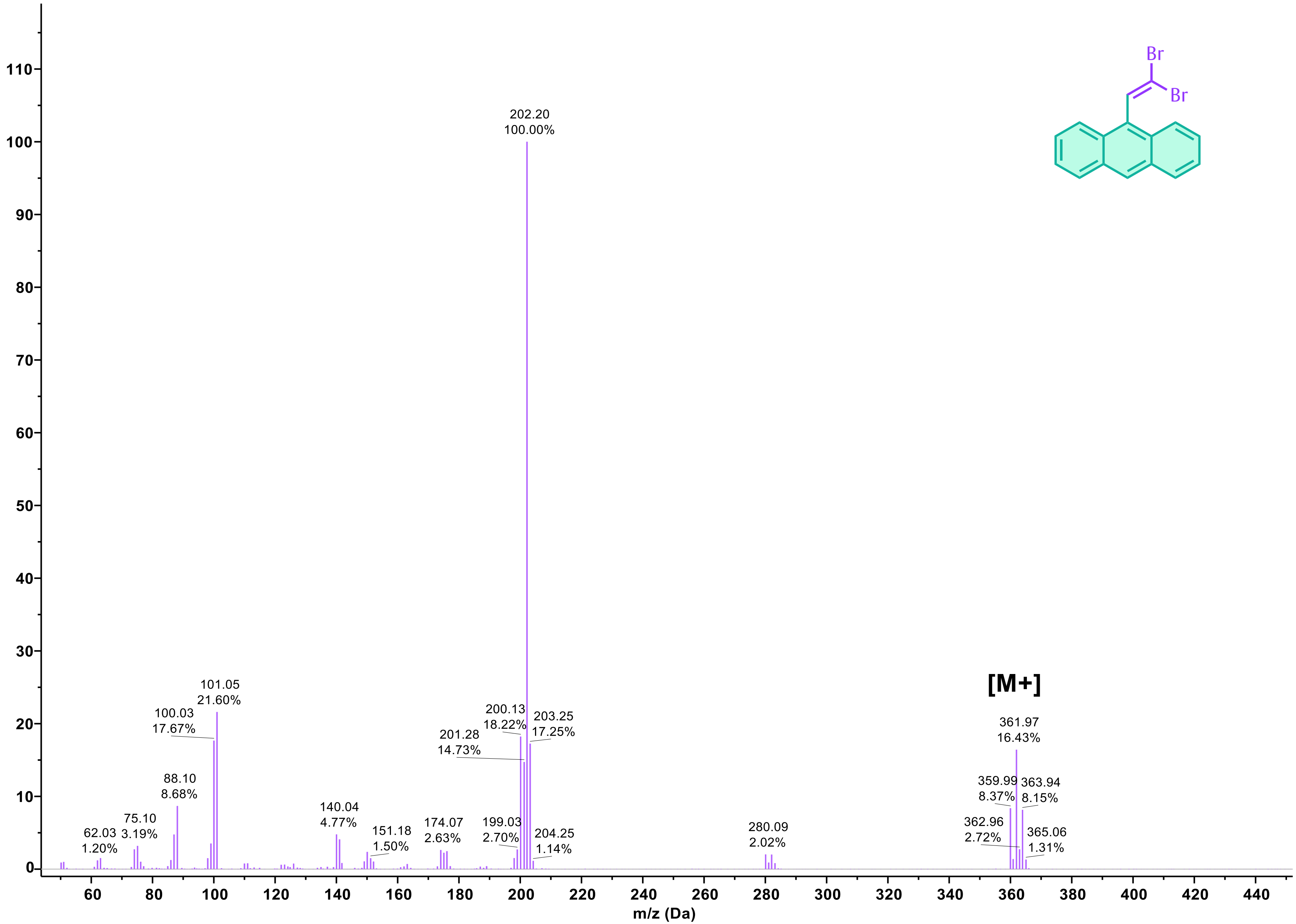

Figure S39. Mass spectrum of compound **4h**.

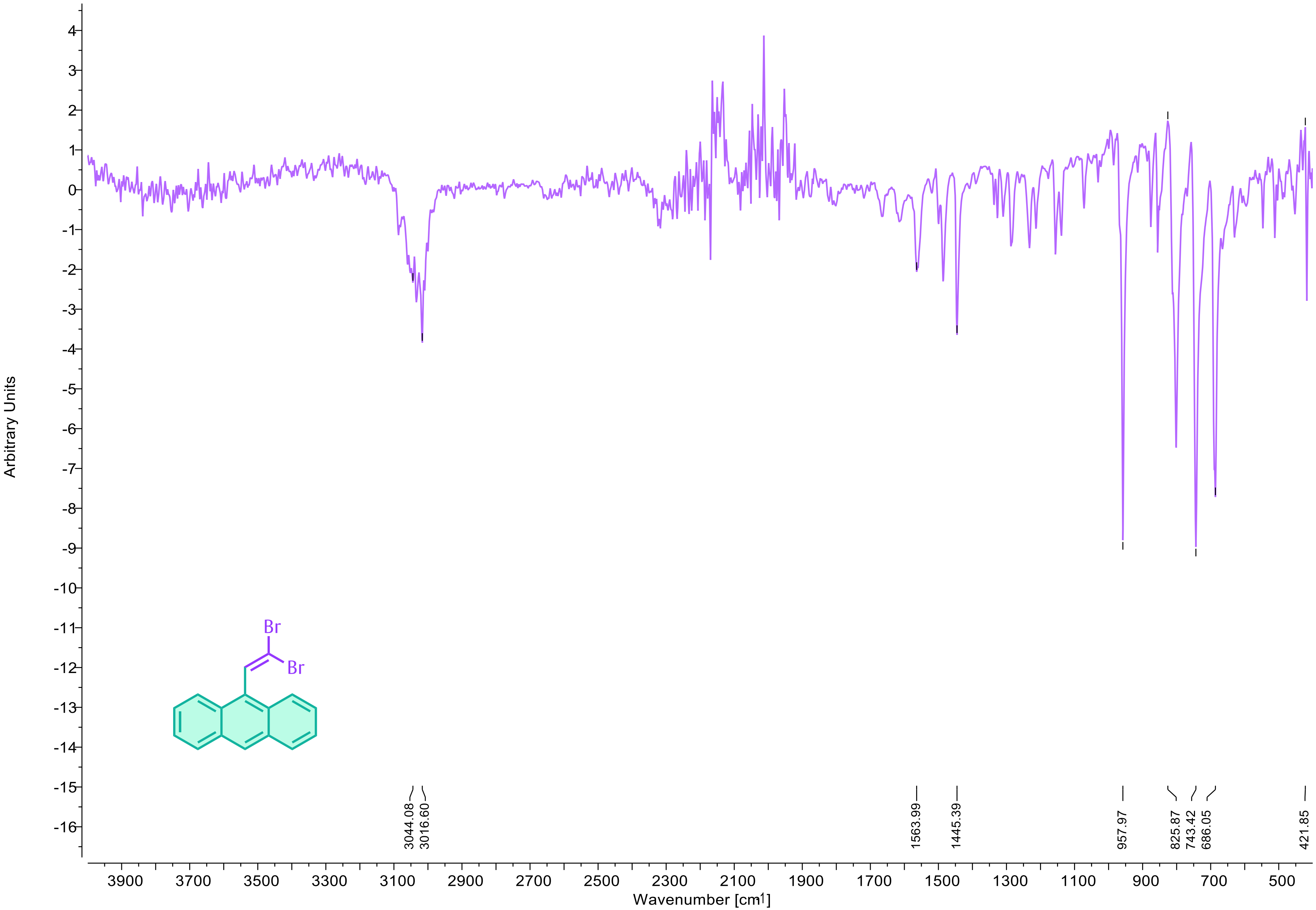

Figure S40. FT-IR spectrum of compound **4h**.

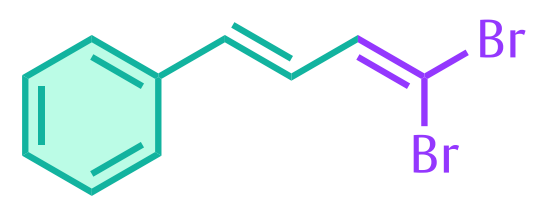

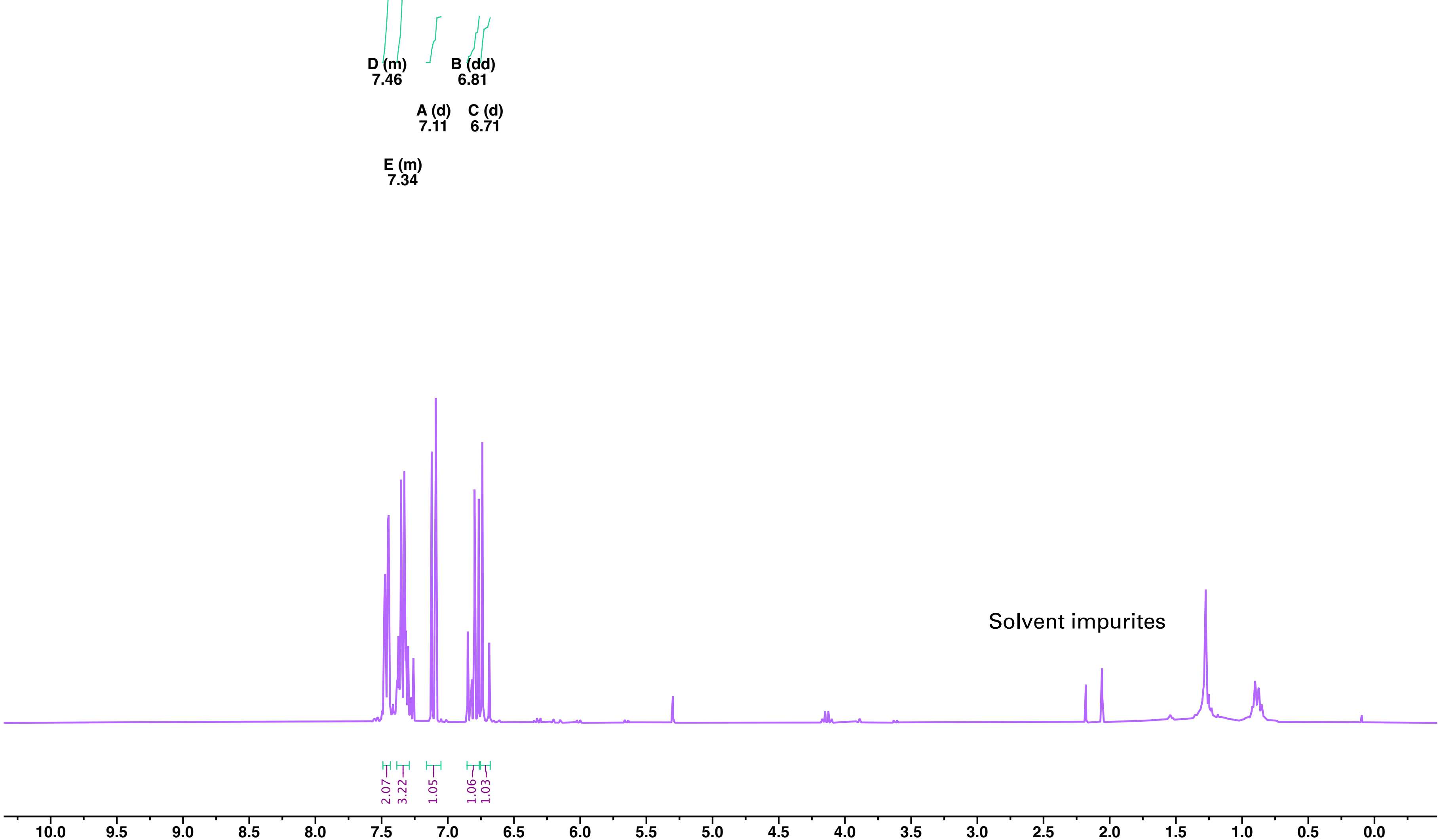

Figure S41. ^{1}H NMR spectrum (300 MHz, $CDCl_3$) of compound **4i**.

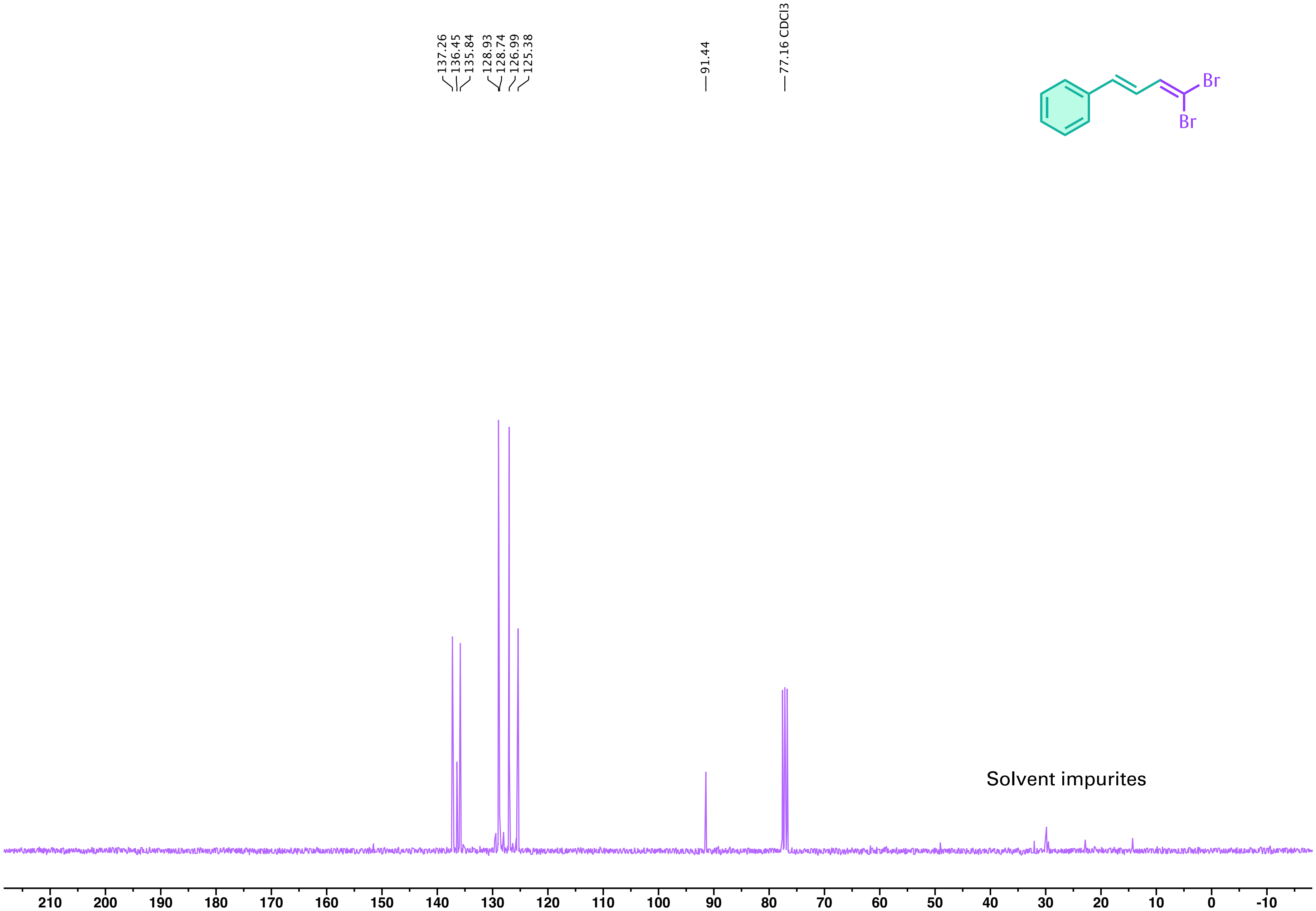

Figure S42. $^{13}C\{^{1}H\}$ NMR spectrum (101 MHz, $CDCl_3$) of compound **4i**.

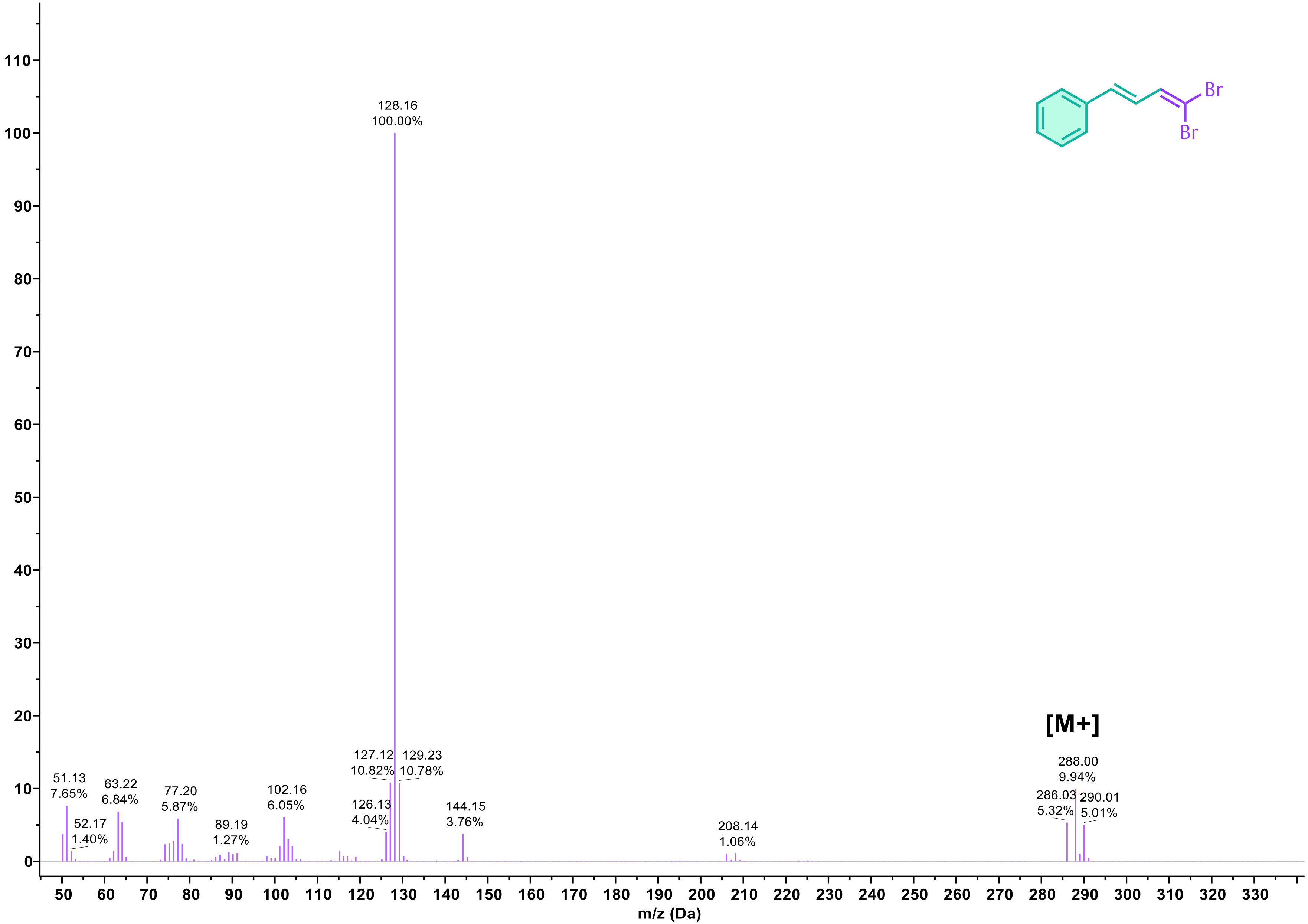

Figure S43. Mass spectrum of compound **4i**.

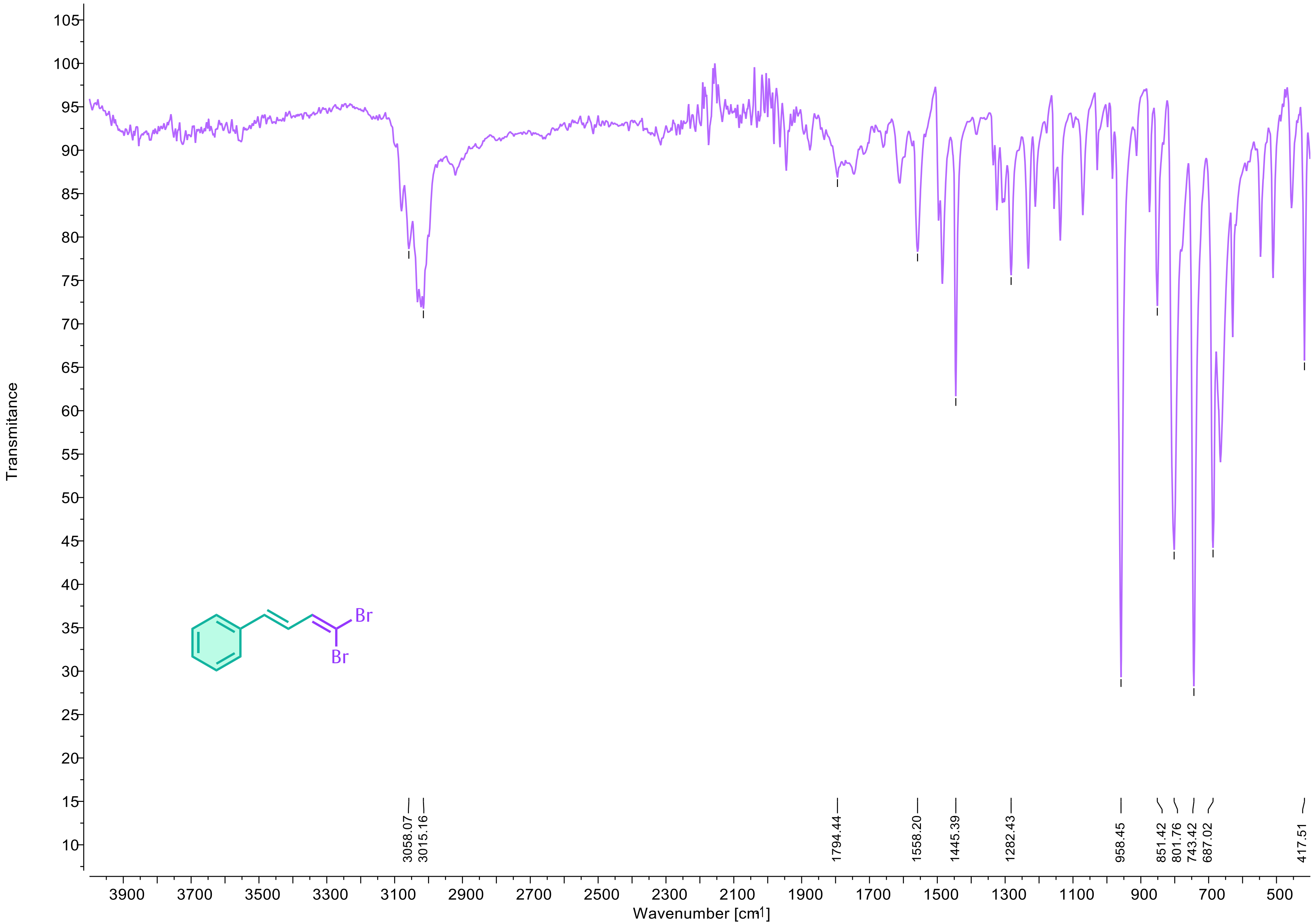

Figure S44. FT-IR spectrum of compound **4i**.

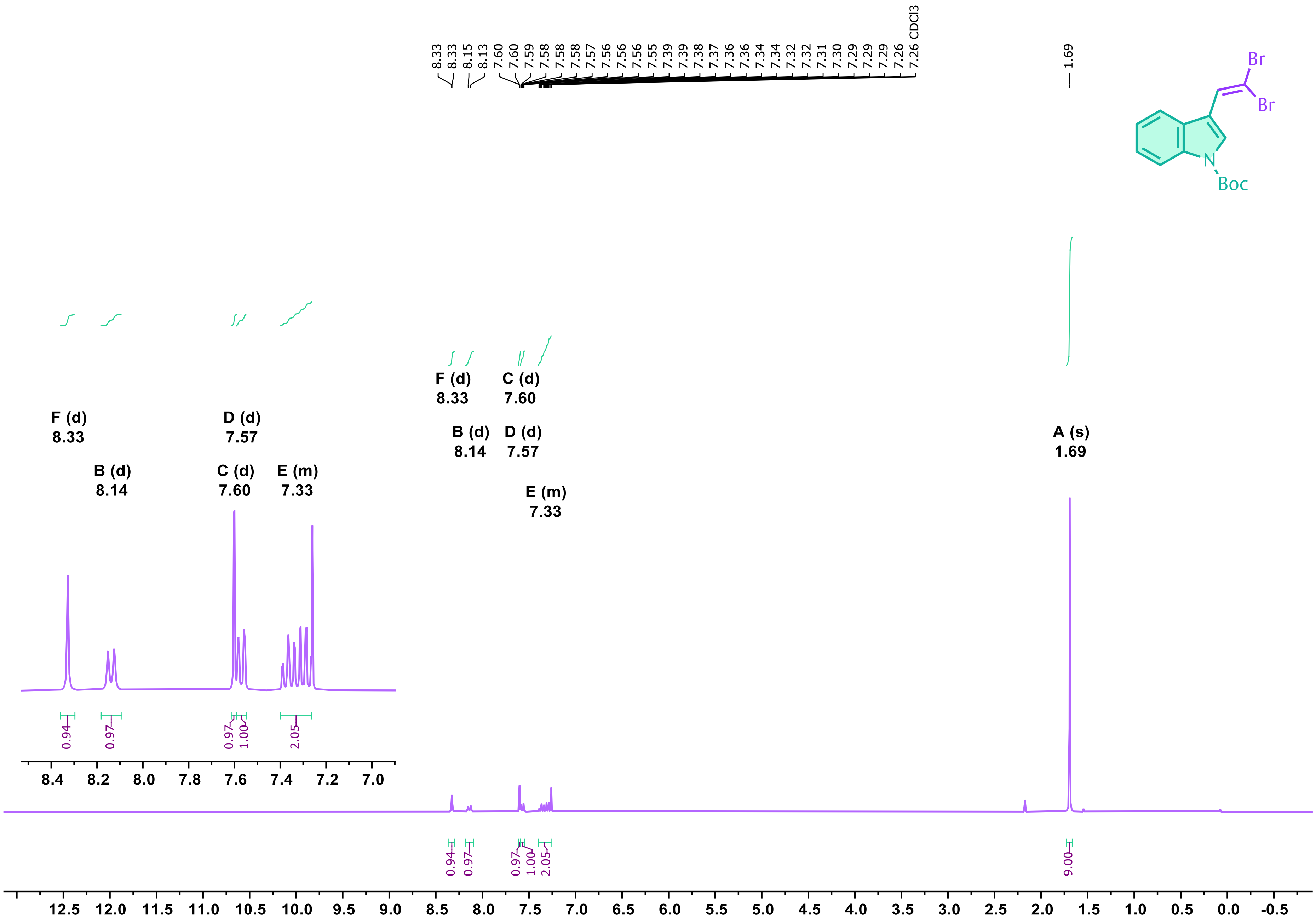

Figure S45. ^1H NMR spectrum (300 MHz, $CDCl_3$) of compound **4j**.

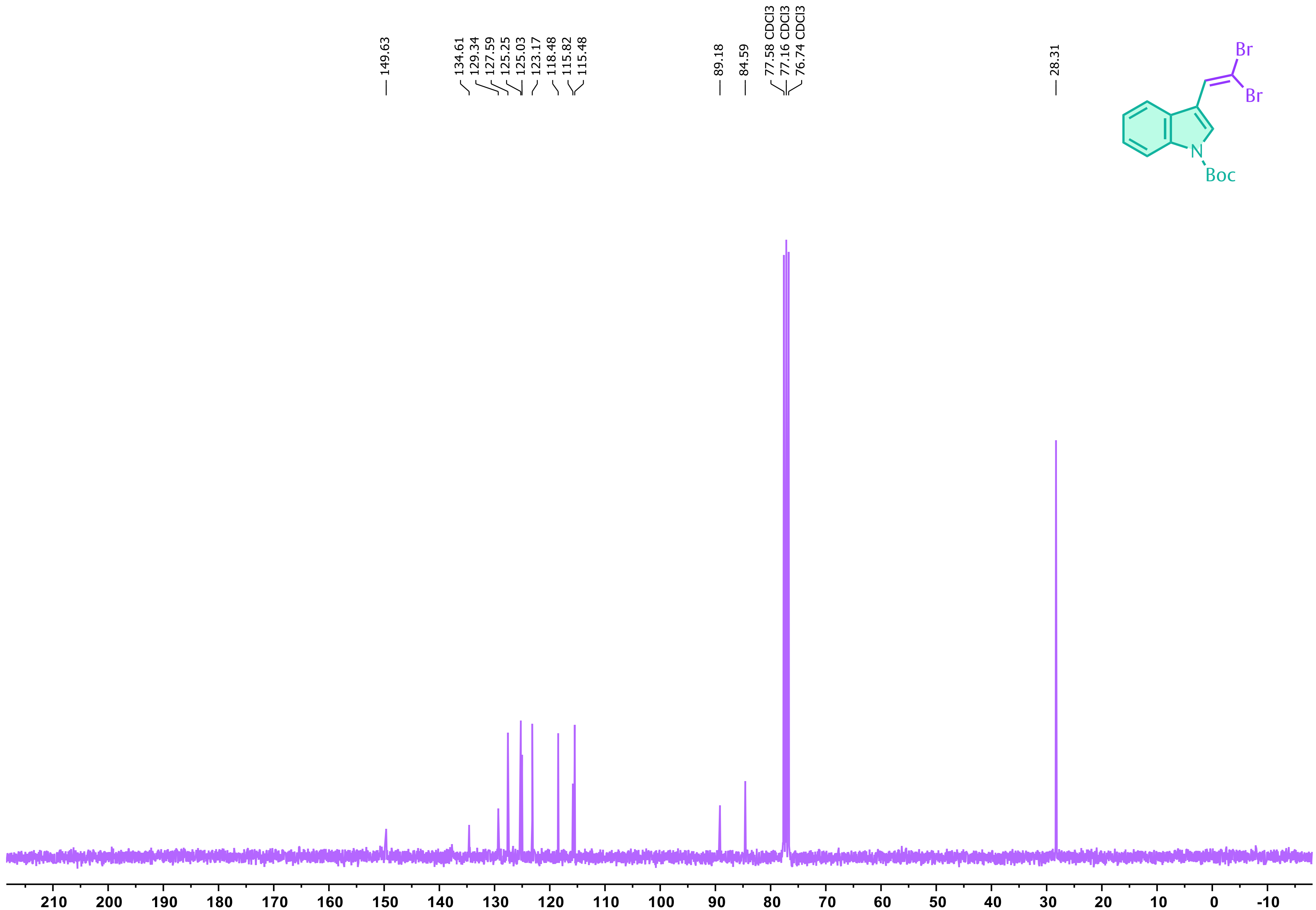

Figure S46. $^{13}C\{^1H\}$ NMR spectrum (101 MHz, $CDCl_3$) of compound **4j**.

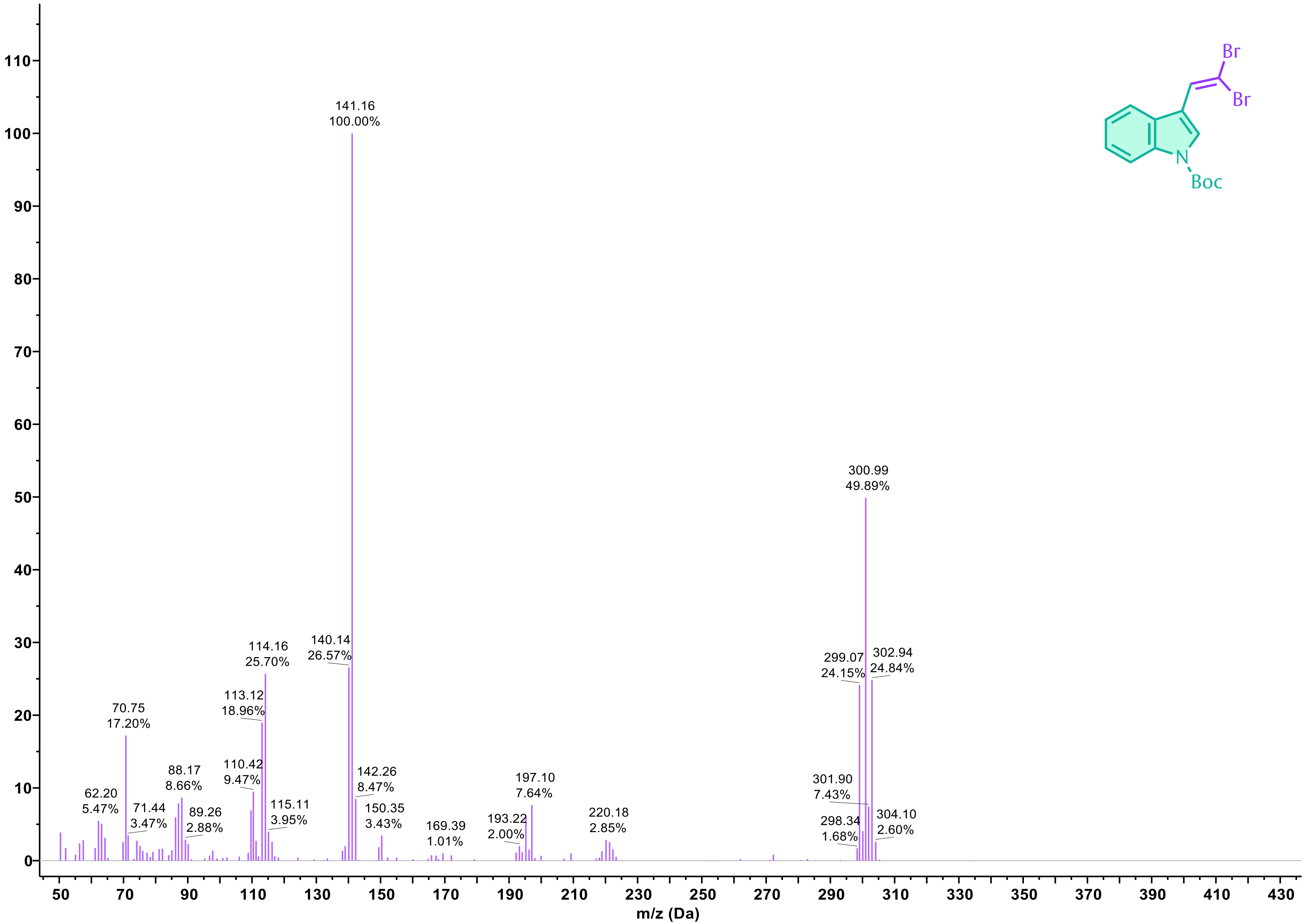

Figure S47. Mass spectrum of compound **4j**.

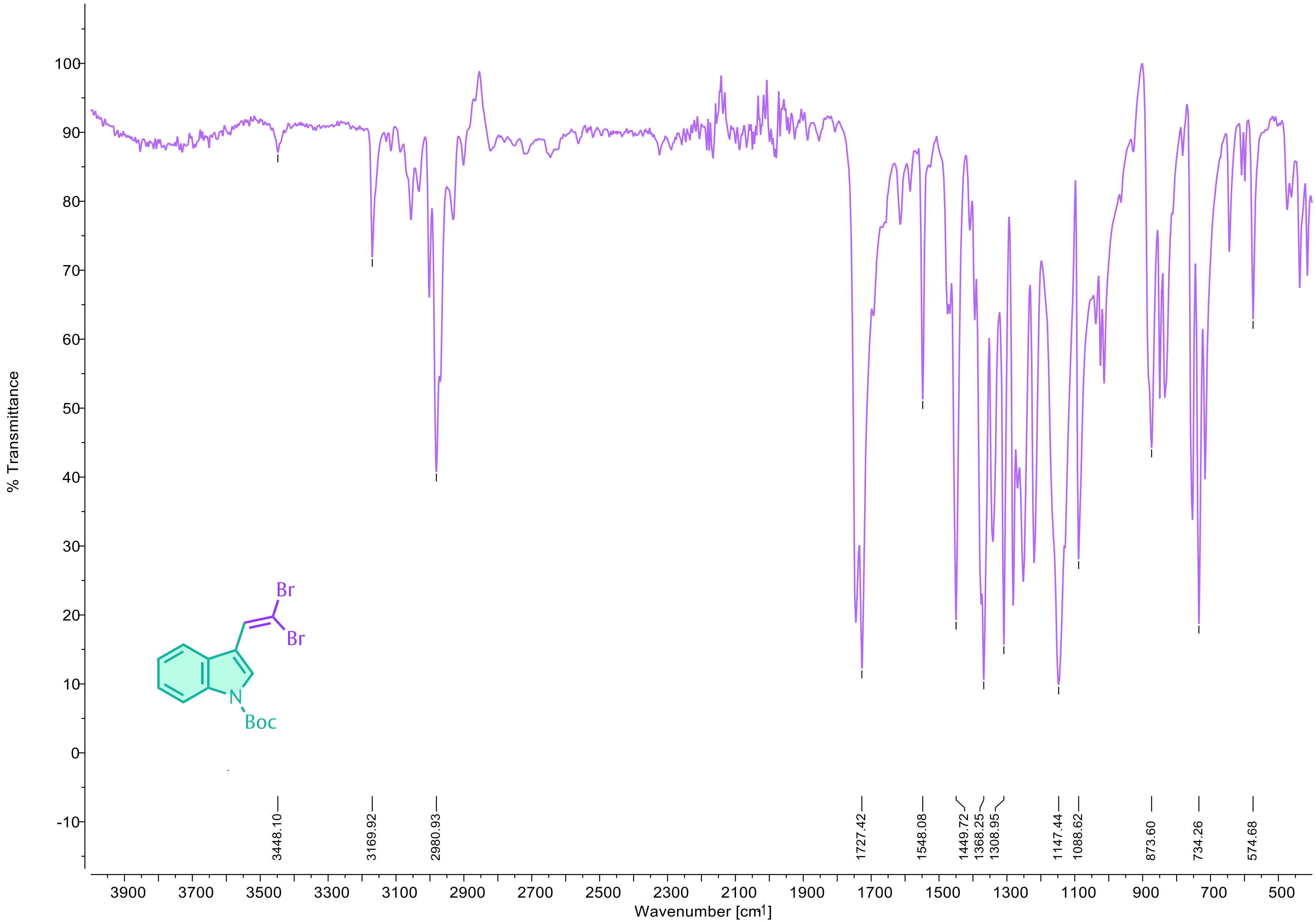

Figure S48. FT-IR spectrum of compound **4j**.

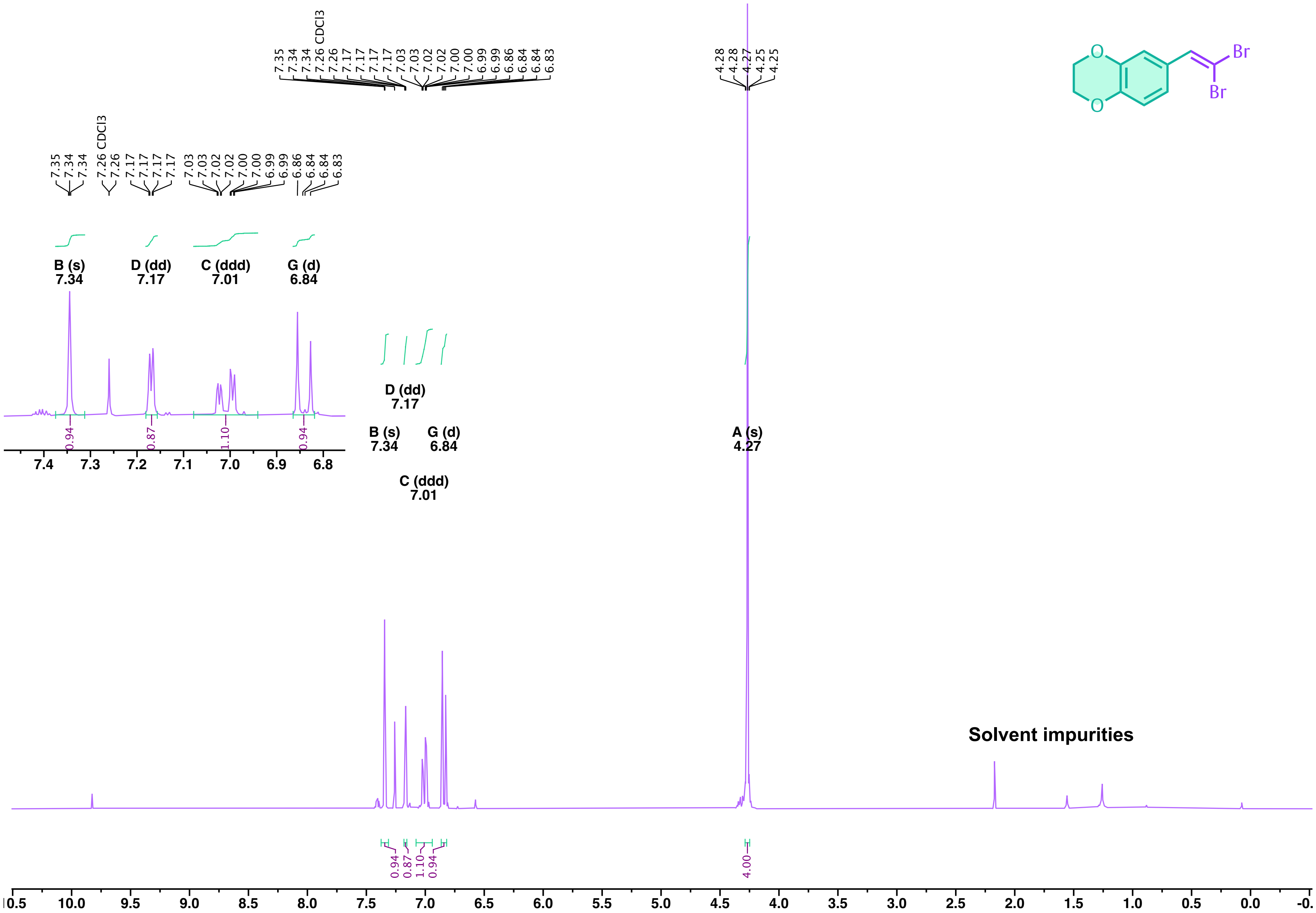

Figure S49. ^{1}H NMR spectrum (300 MHz, $CDCl_3$) of compound **4k**.

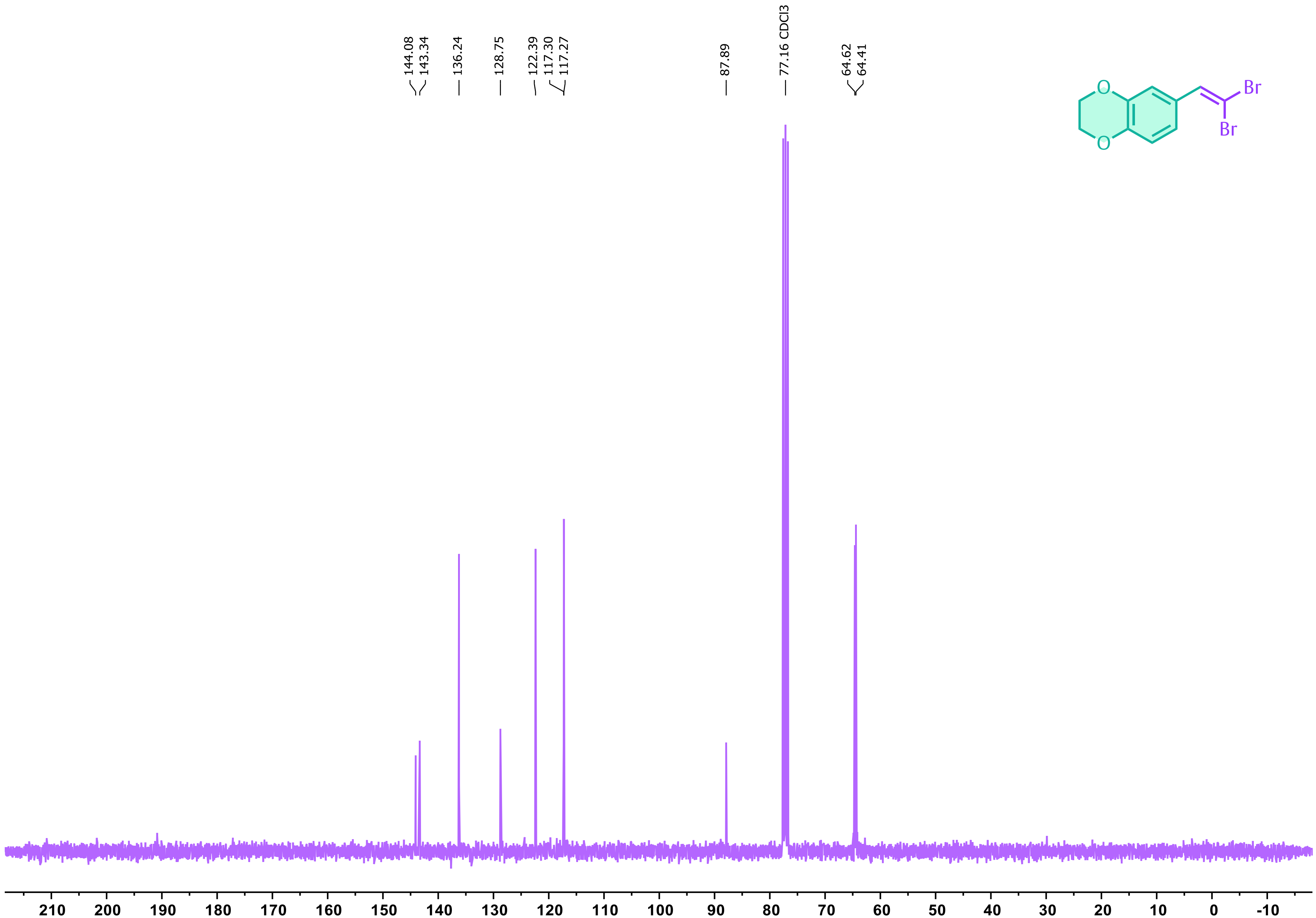

Figure S51. $^{13}C\{^{1}H\}$ NMR spectrum (101 MHz, $CDCl_3$) of compound **4k**.

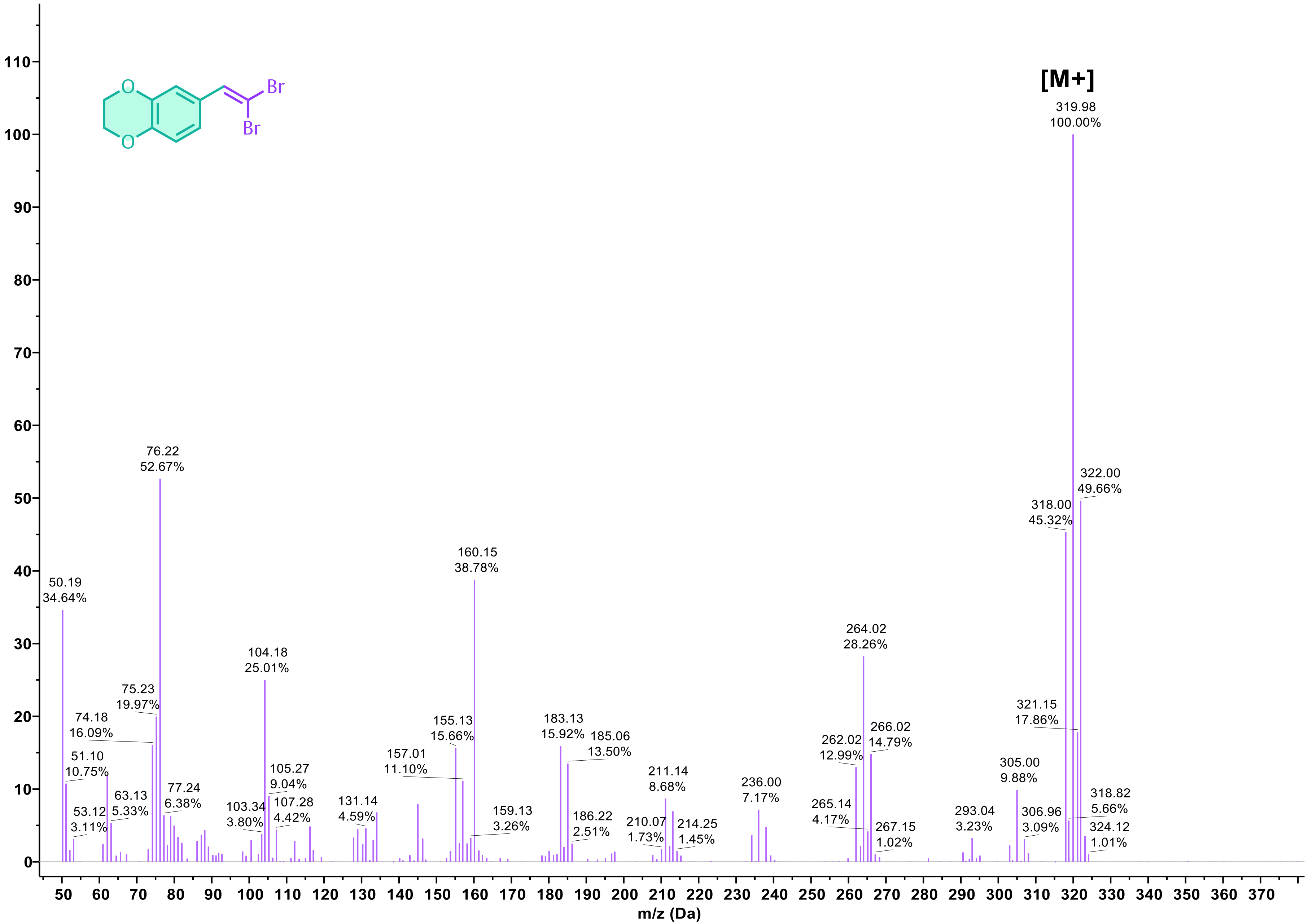

Figure S52. Mass spectrum of compound **4k**.

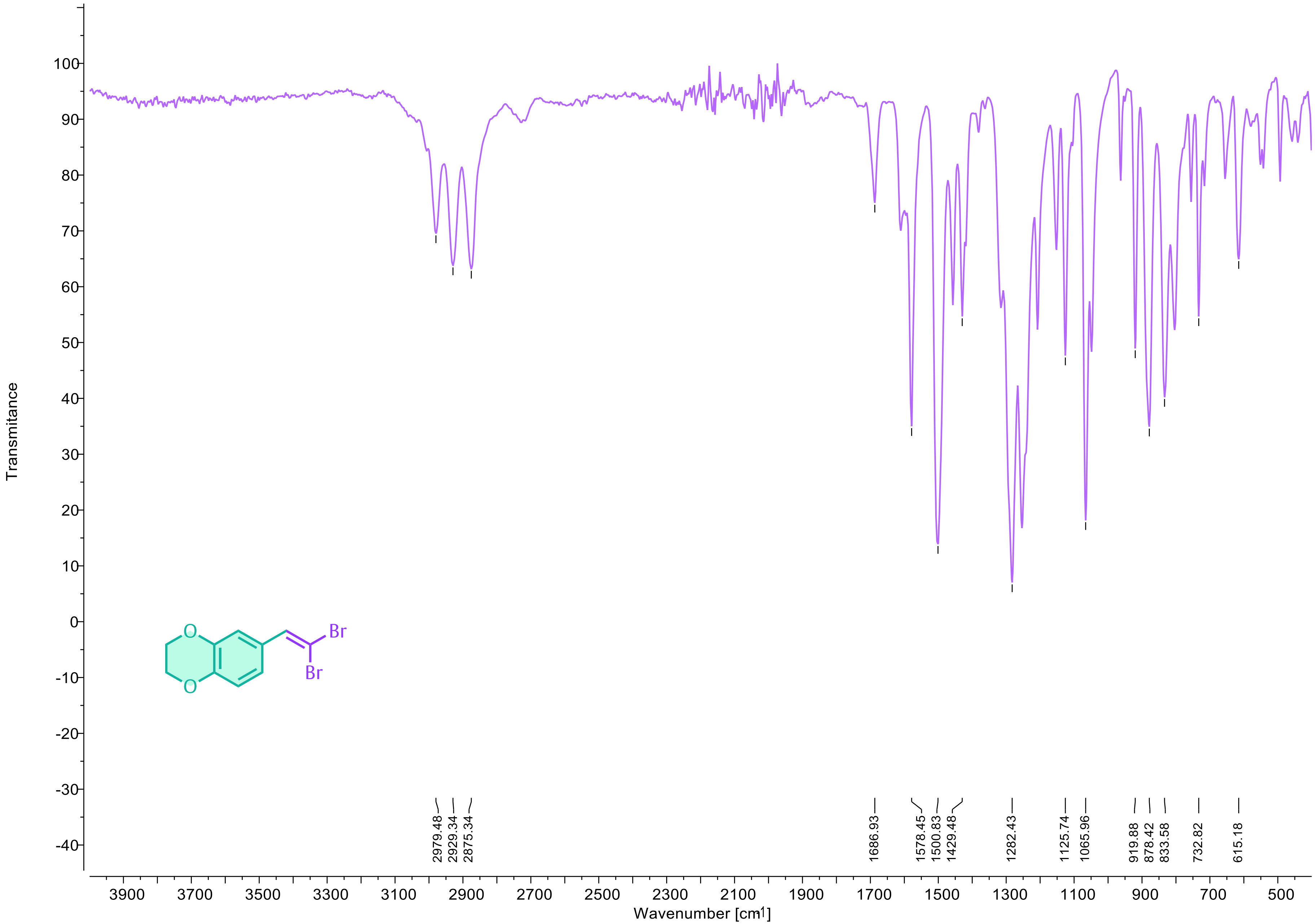

Figure S53. FT-IR spectrum of compound **4k**.

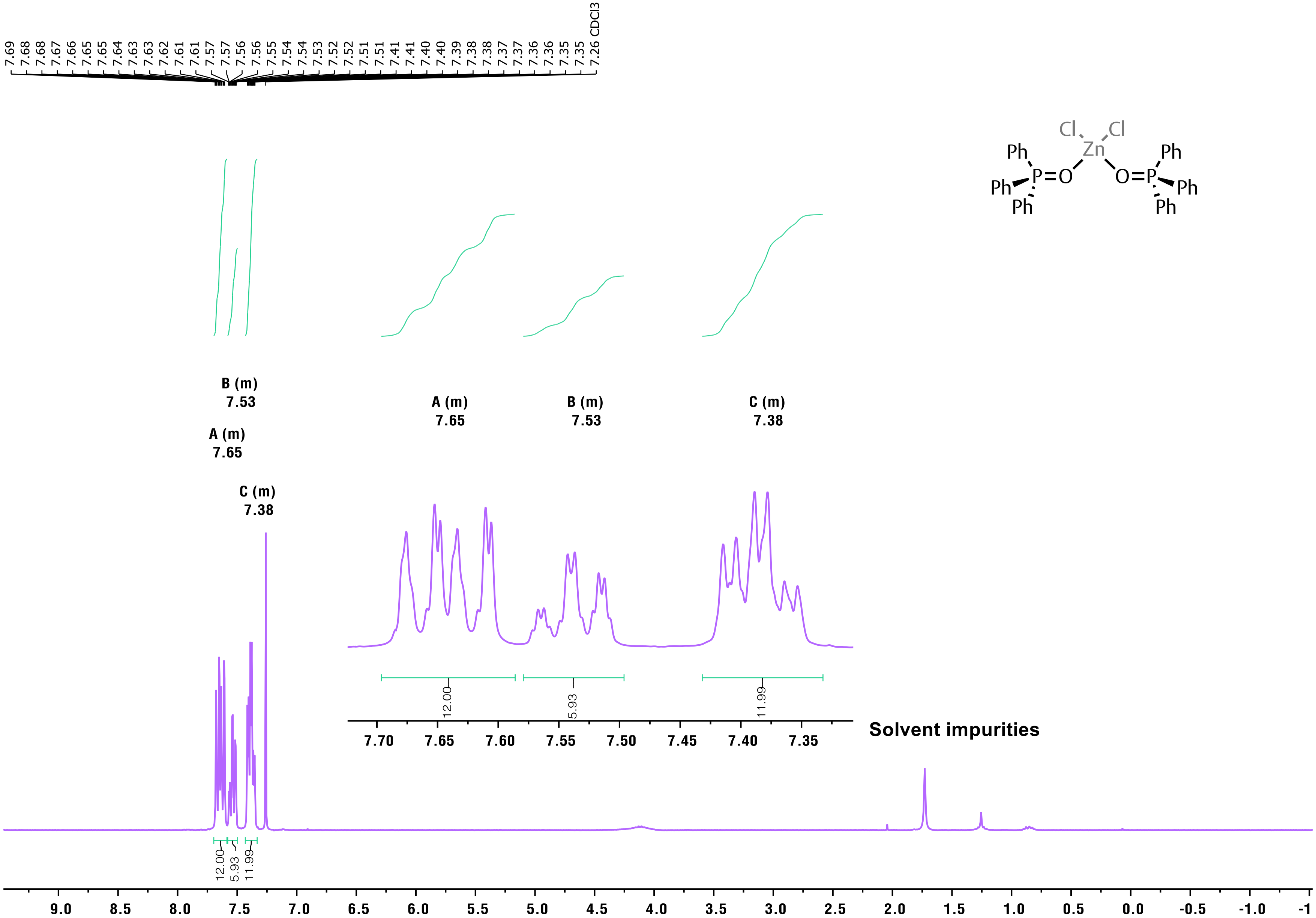

Figure S54. ^{1}H NMR spectrum (300 MHz, $CDCl_3$) of compound **5**.

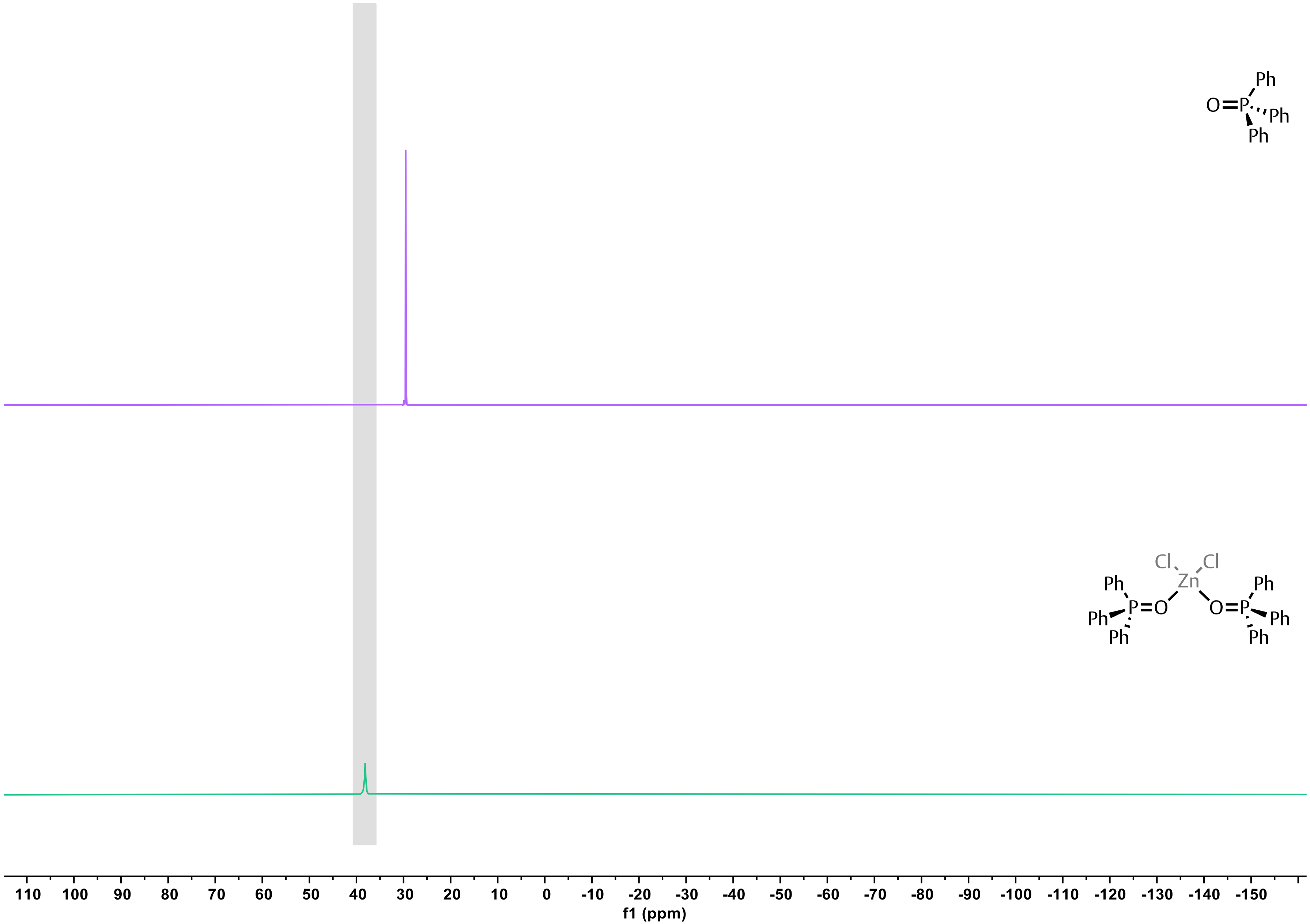

Figure S55. Comparison of $^{31}P\{^1H\}$ NMR spectra (162 MHz, $CDCl_3$) of compound **TPPO** and compound **5**.

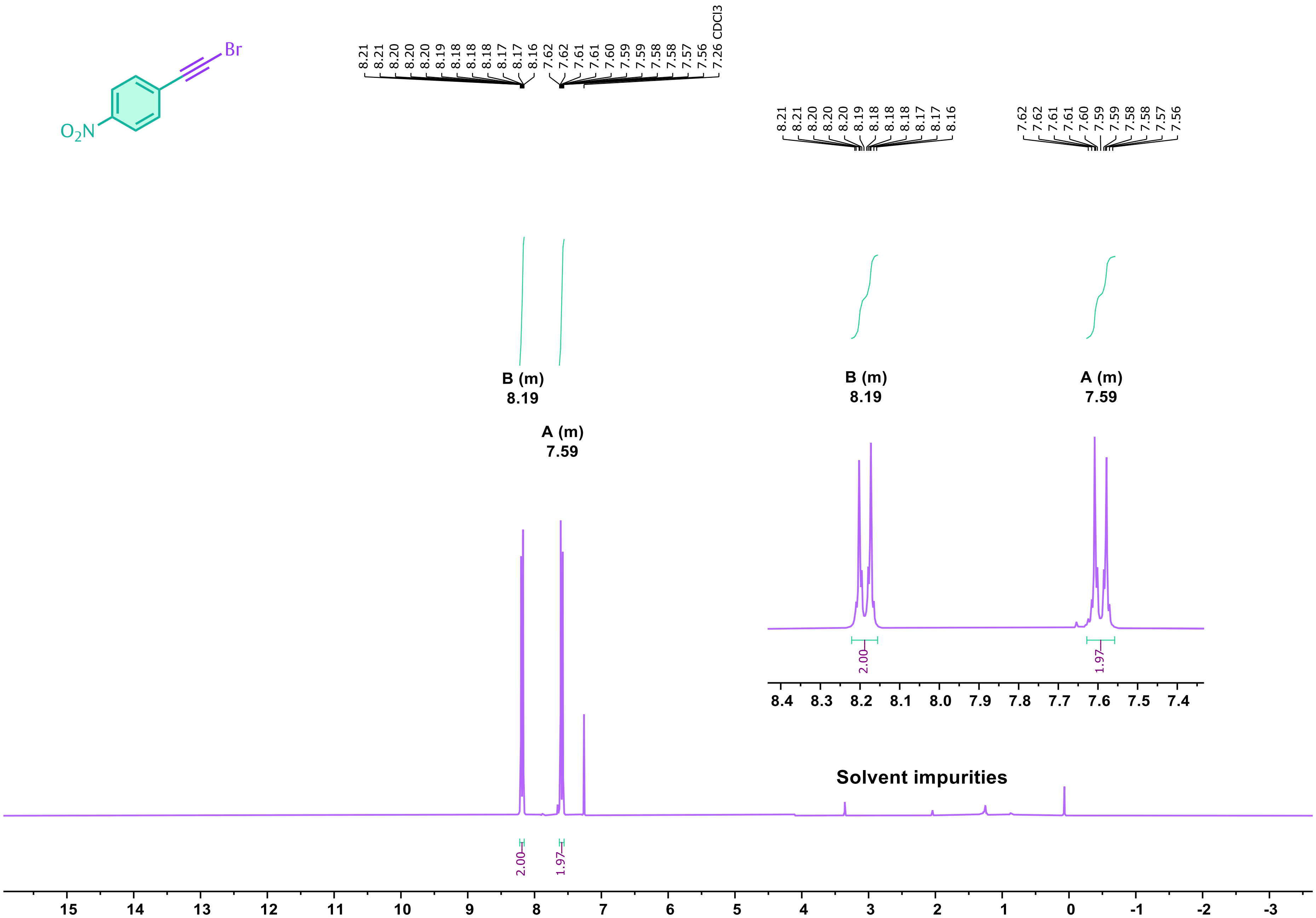

Figure S56. ^{1}H NMR spectrum (300 MHz, $CDCl_3$) of compound **6a**.

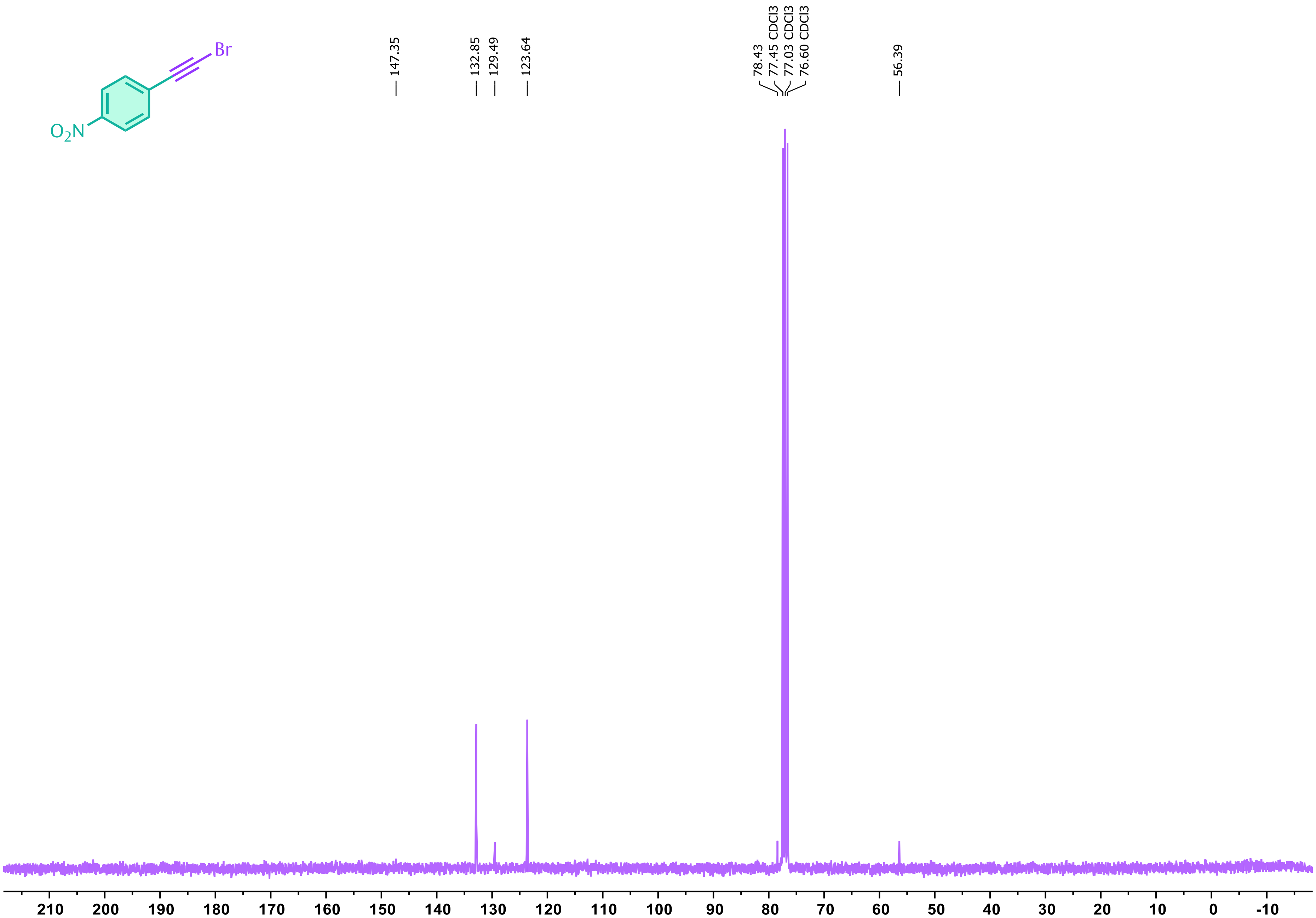

Figure S57. $^{13}C\{^{1}H\}$ NMR spectrum (75 MHz, $CDCl_3$) of compound **6a**.

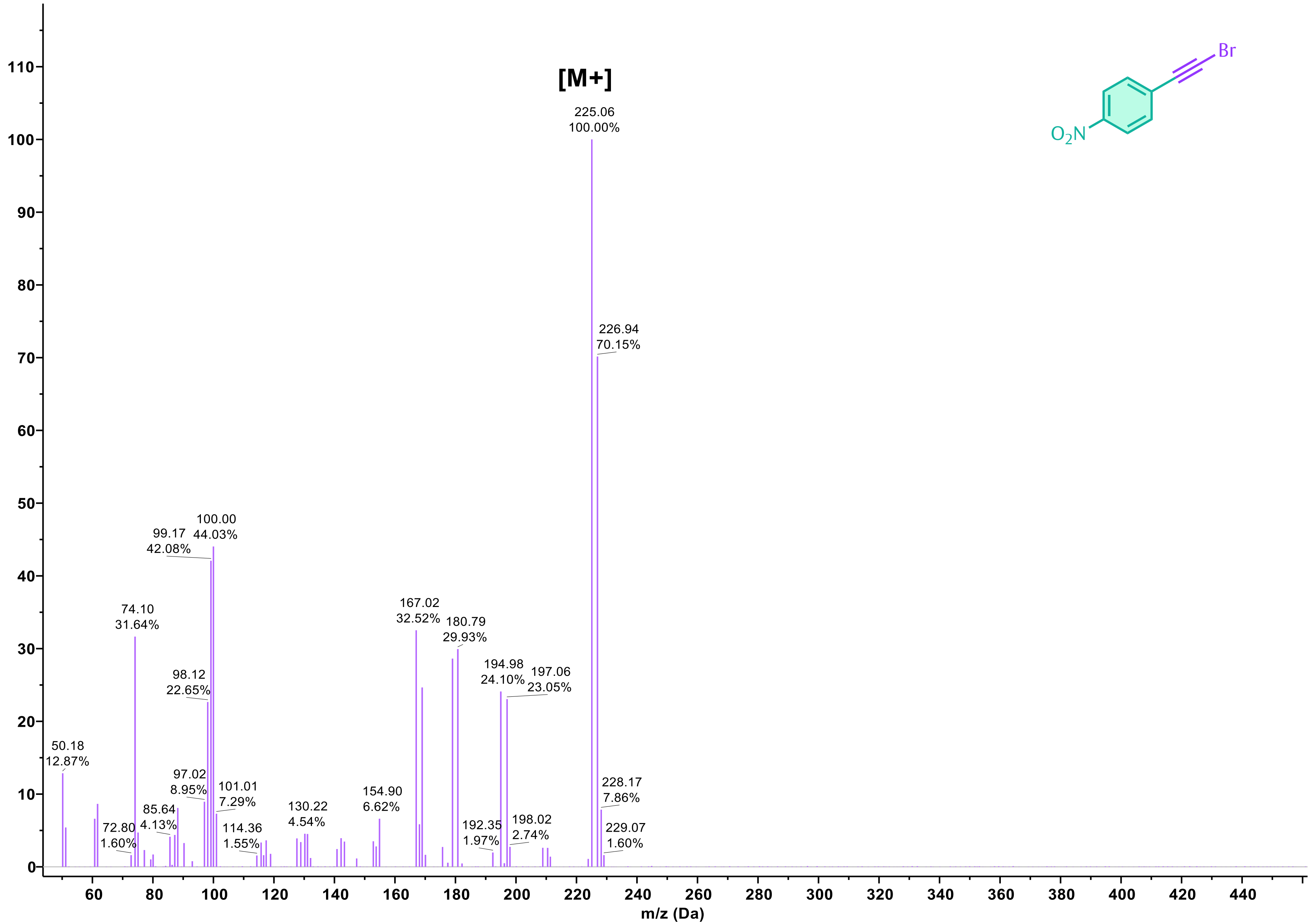

Figure S58. Mass spectrum of compound **6a**.

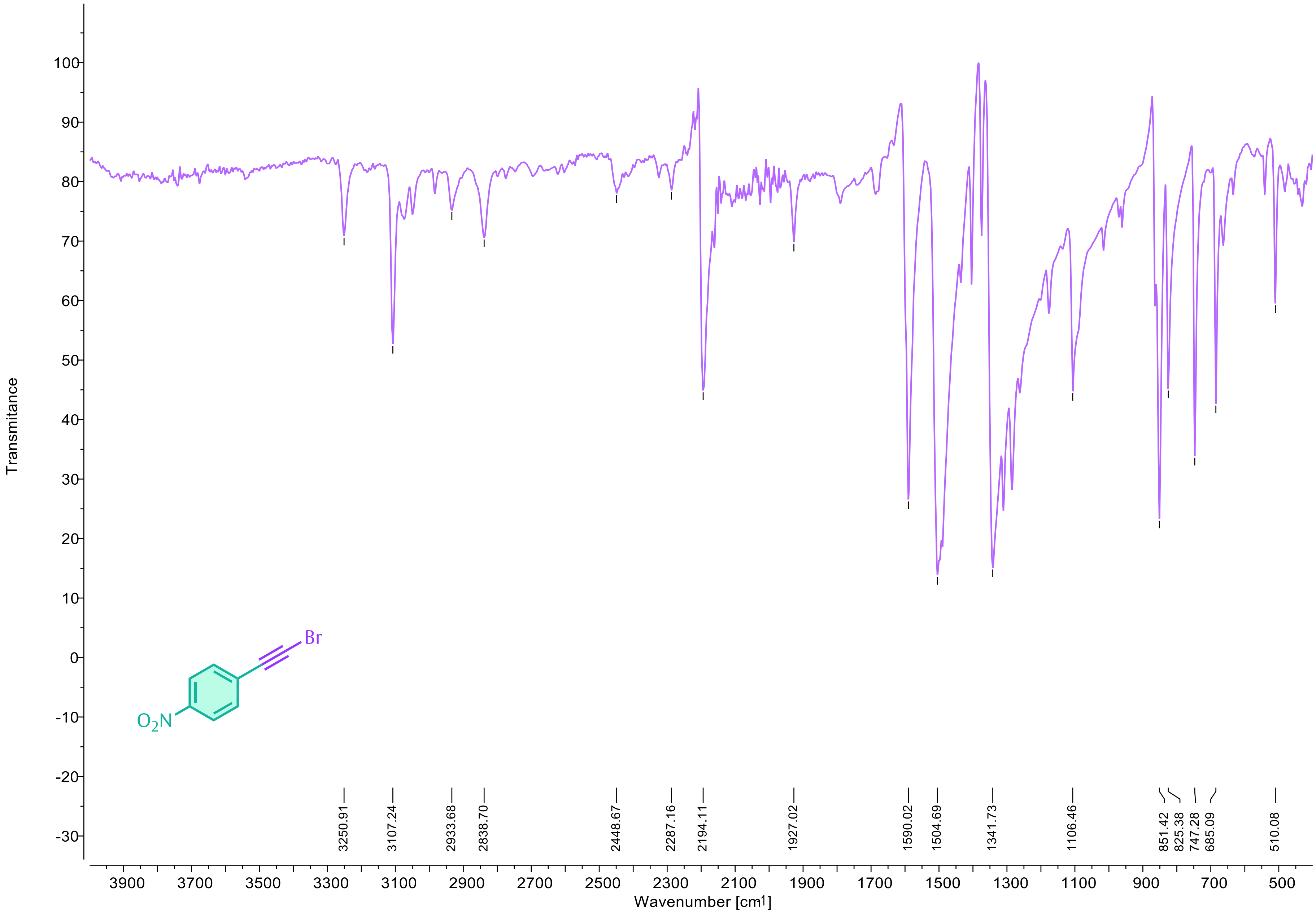

Figure S59. FT-IR spectrum of compound **6a**.

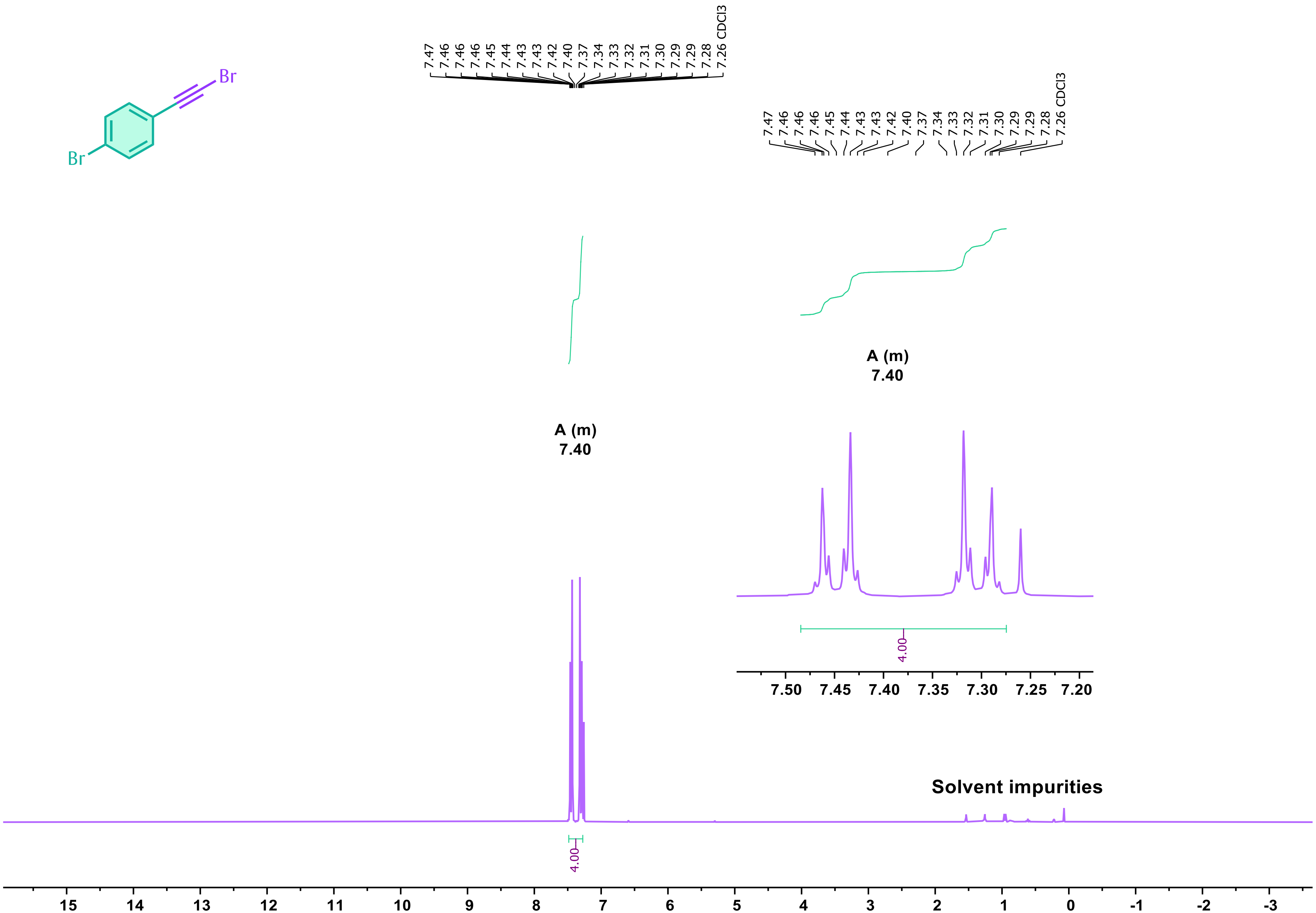

Figure S60. ^{1}H NMR spectrum (300 MHz, $CDCl_3$) of compound **6b**.

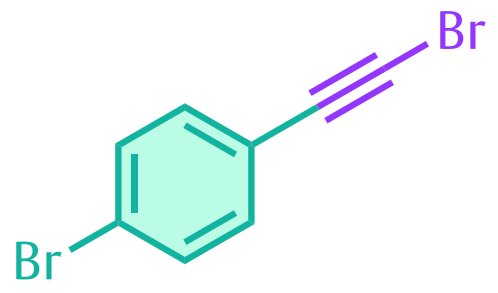

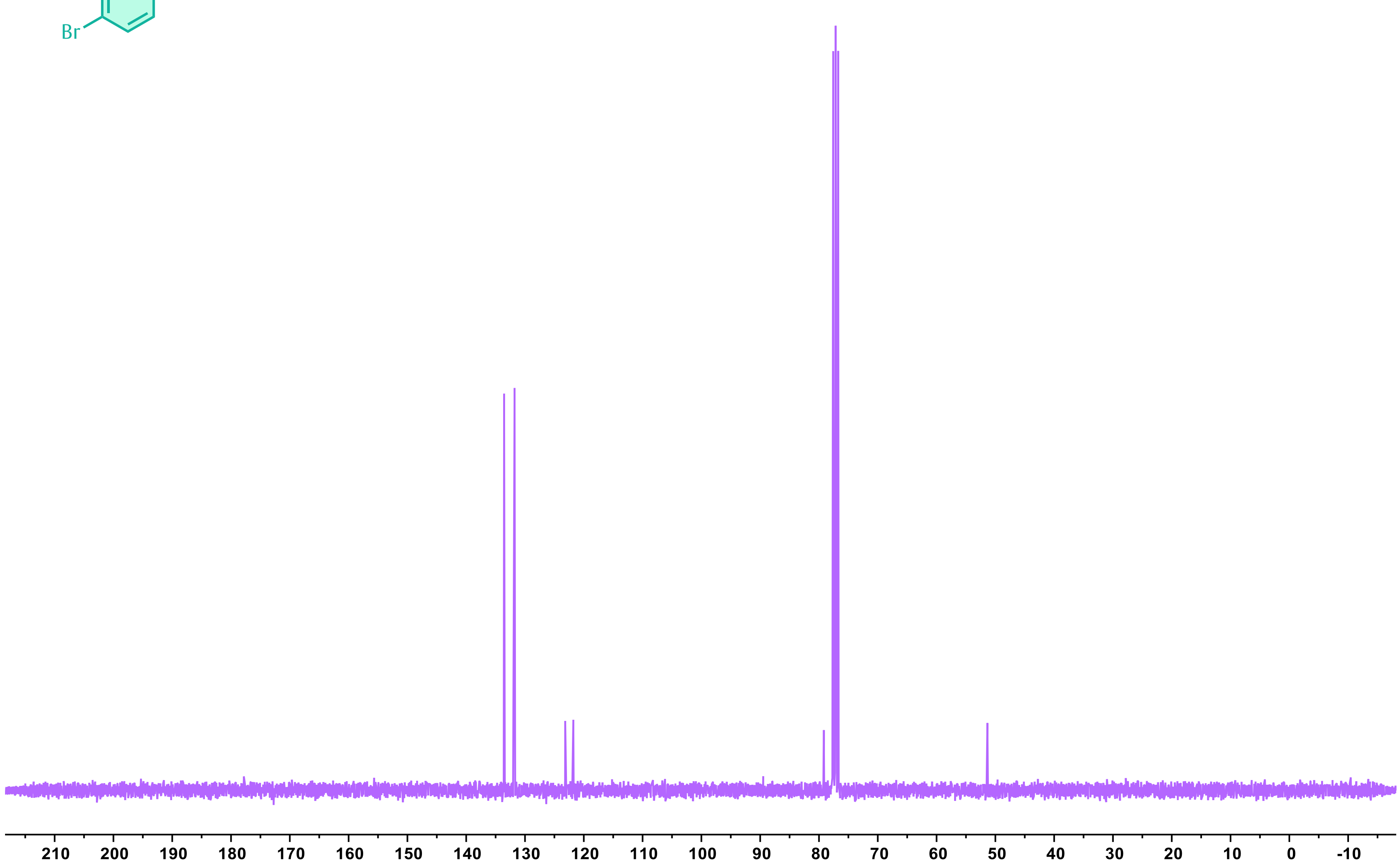

Figure S61. $^{13}C\{^1H\}$ NMR spectrum (75 MHz, $CDCl_3$) of compound **6a**

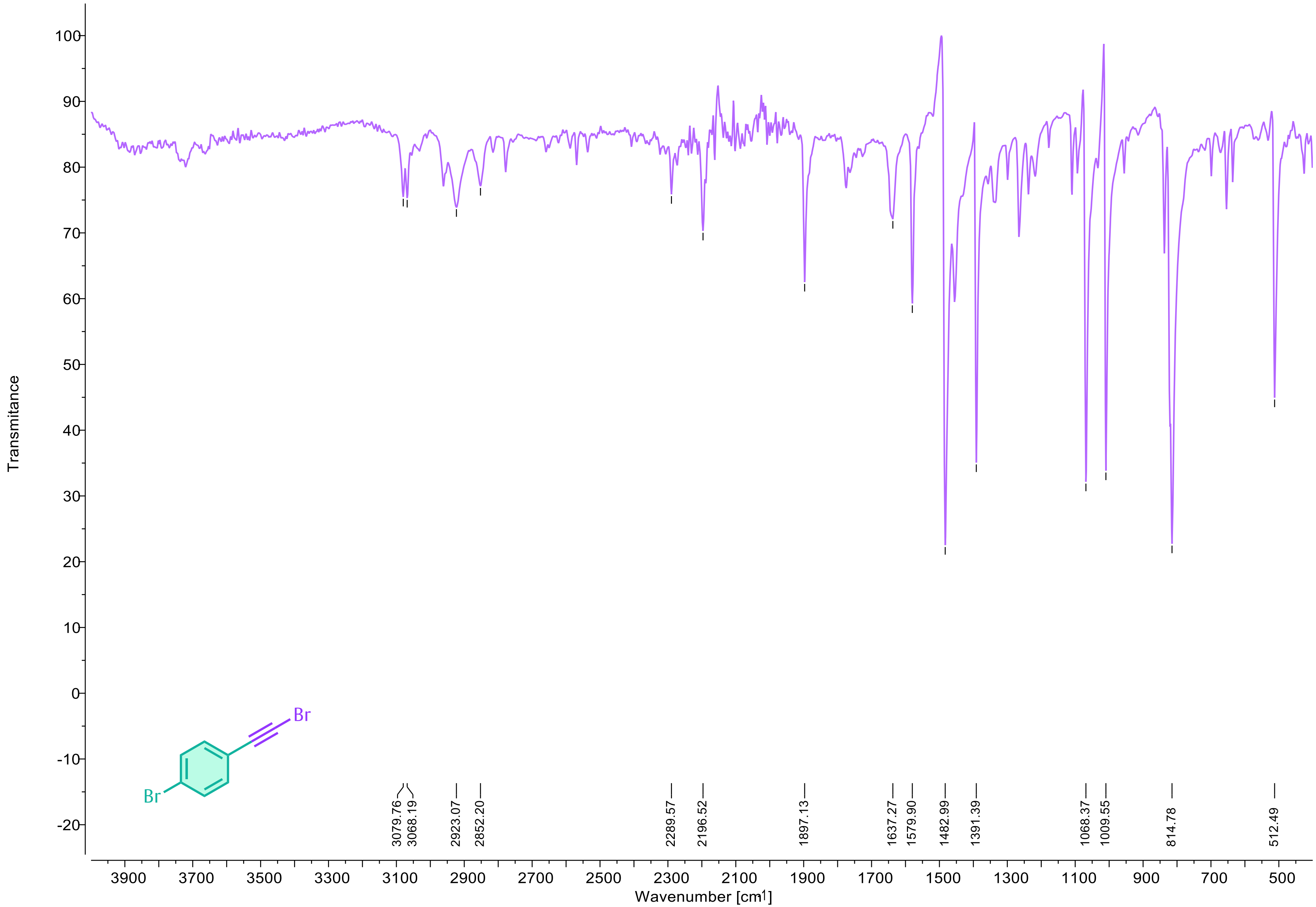

Figure S62. FT-IR spectrum of compound **6b**.

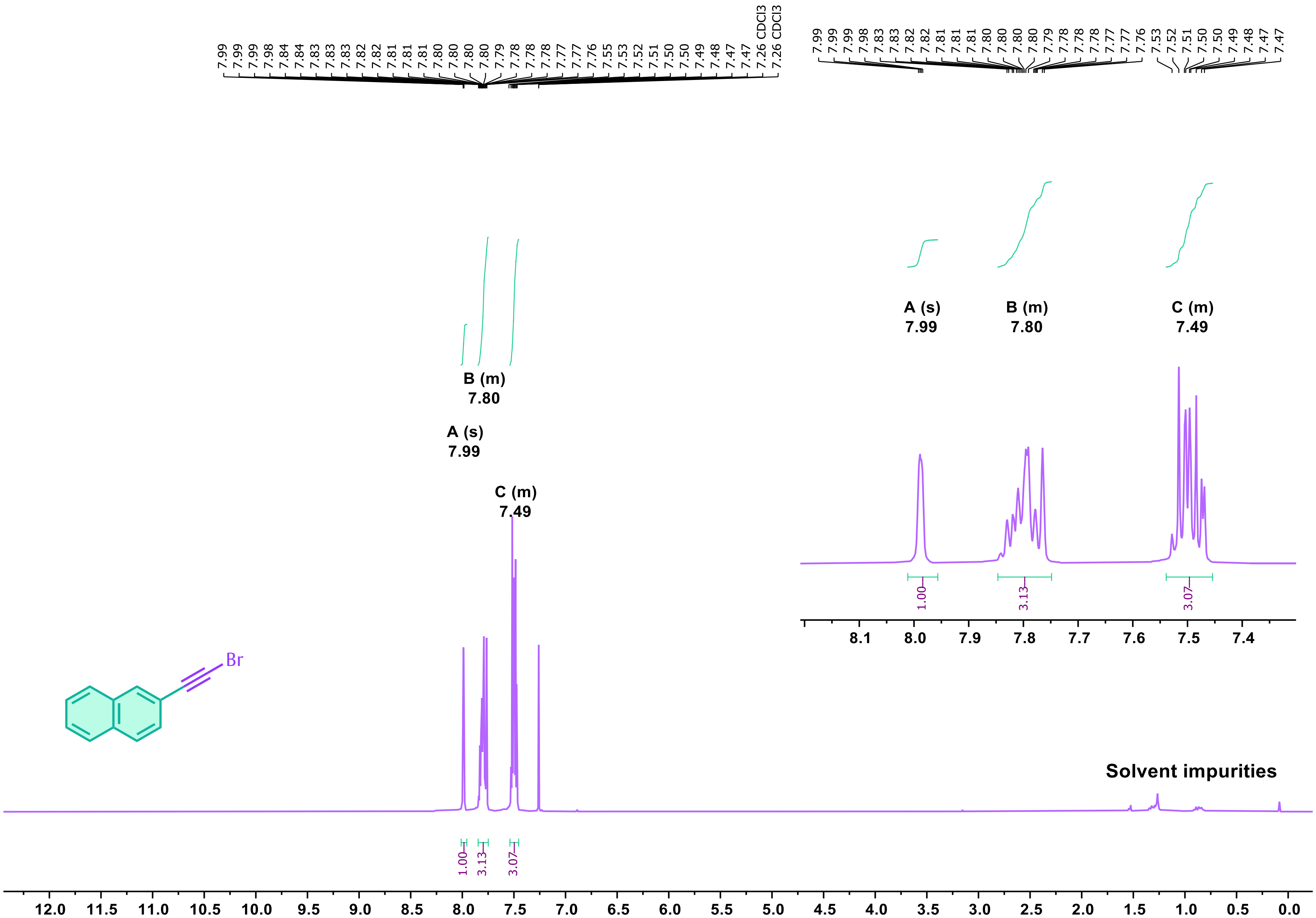

Figure S63. ¹H NMR spectrum (300 MHz, $CDCl_3$) of compound **6c**.

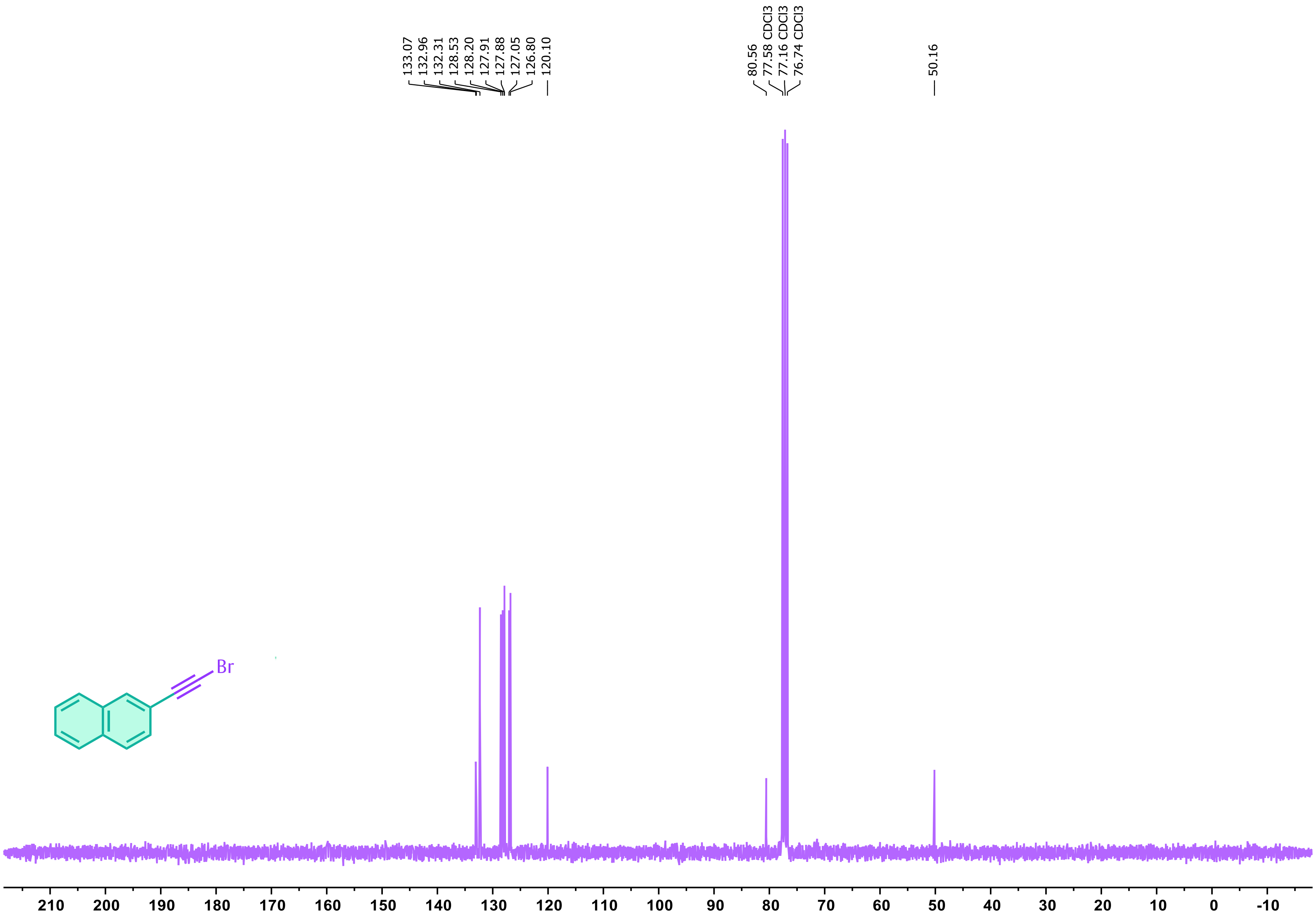

Figure S64. $^{13}C\{^1H\}$ NMR spectrum (75 MHz, $CDCl_3$) of compound **6c**.

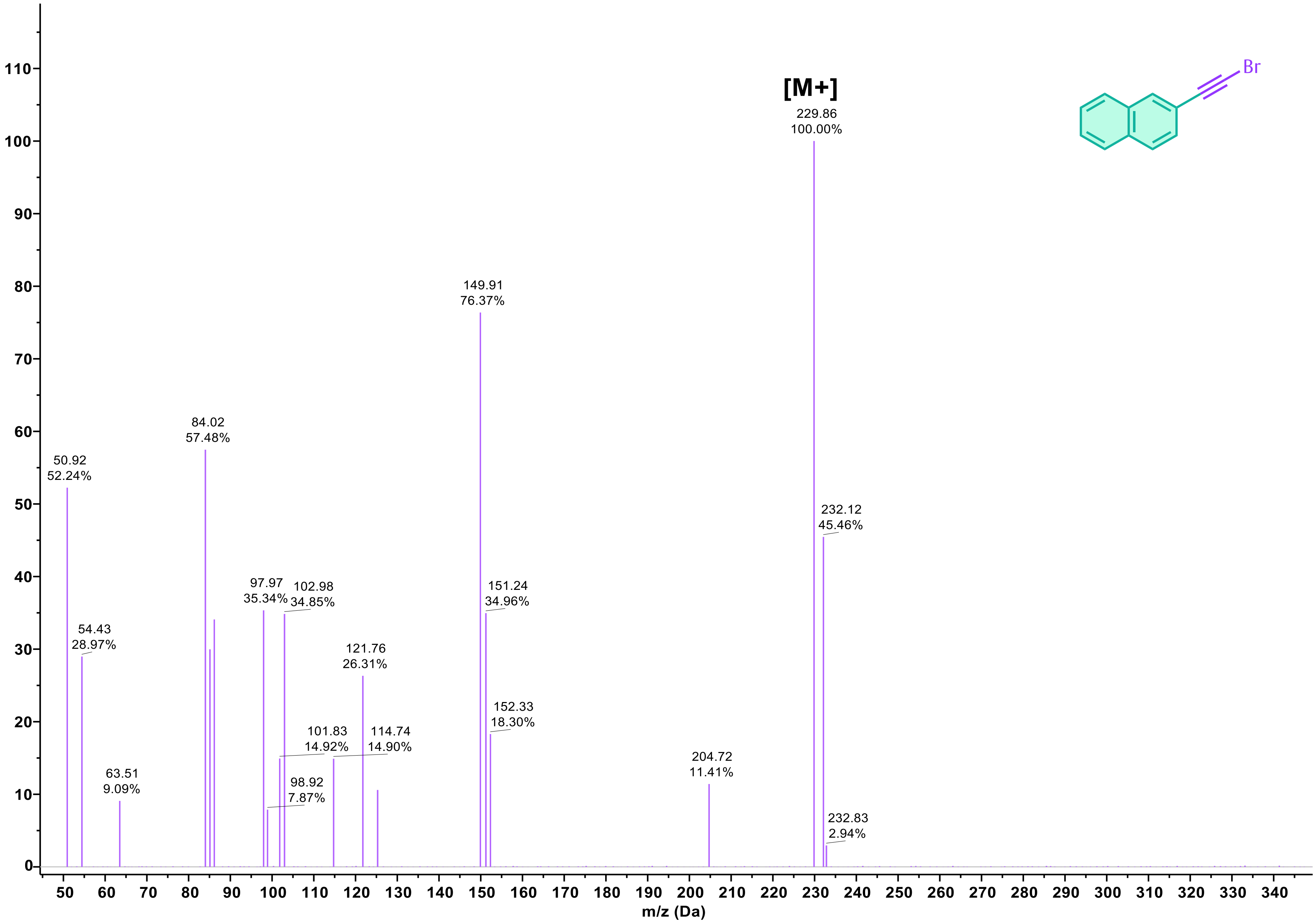

Figure S65. Mass spectrum of compound **6c**.

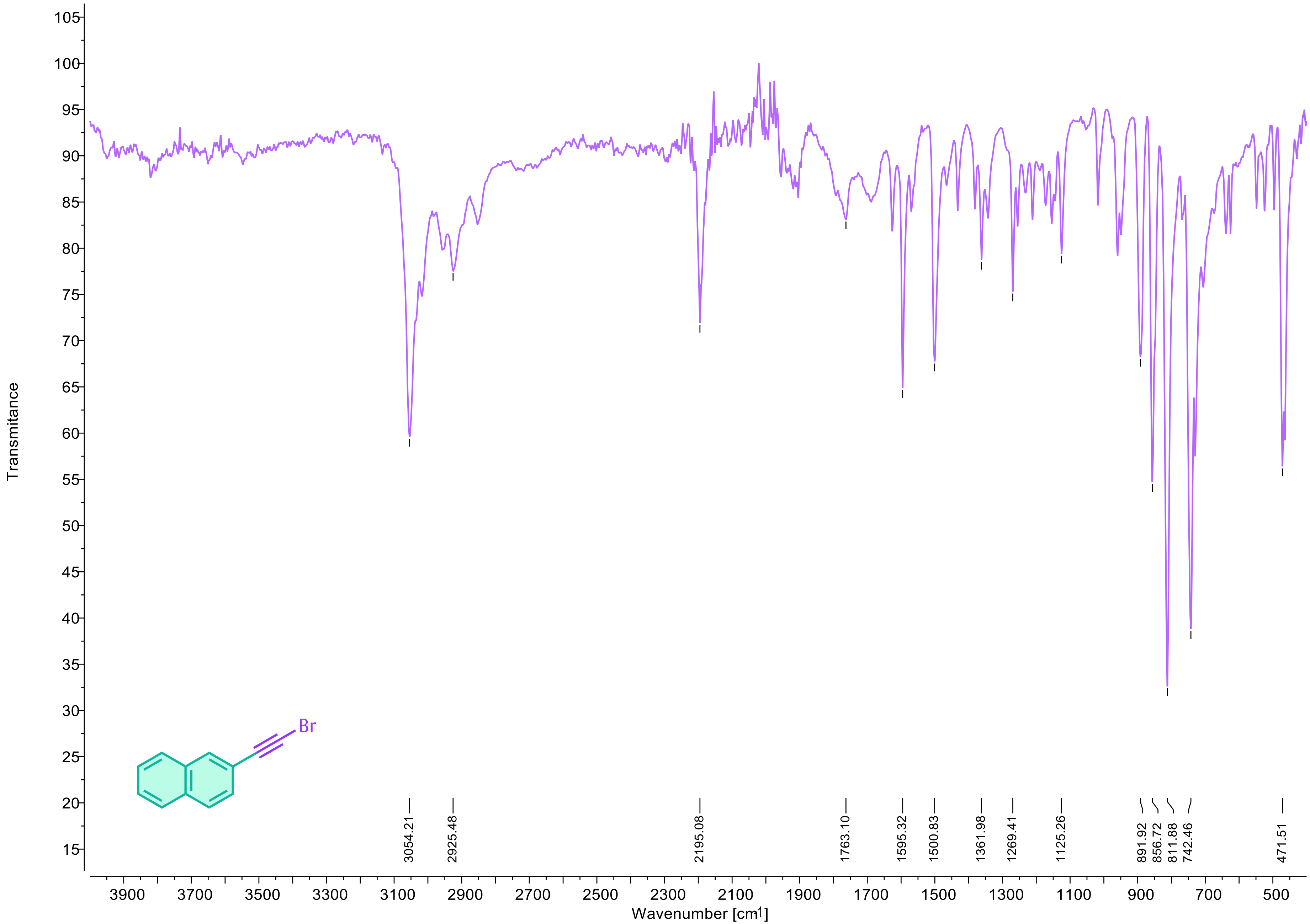

Figure S66. FT-IR spectrum of compound **6c**.

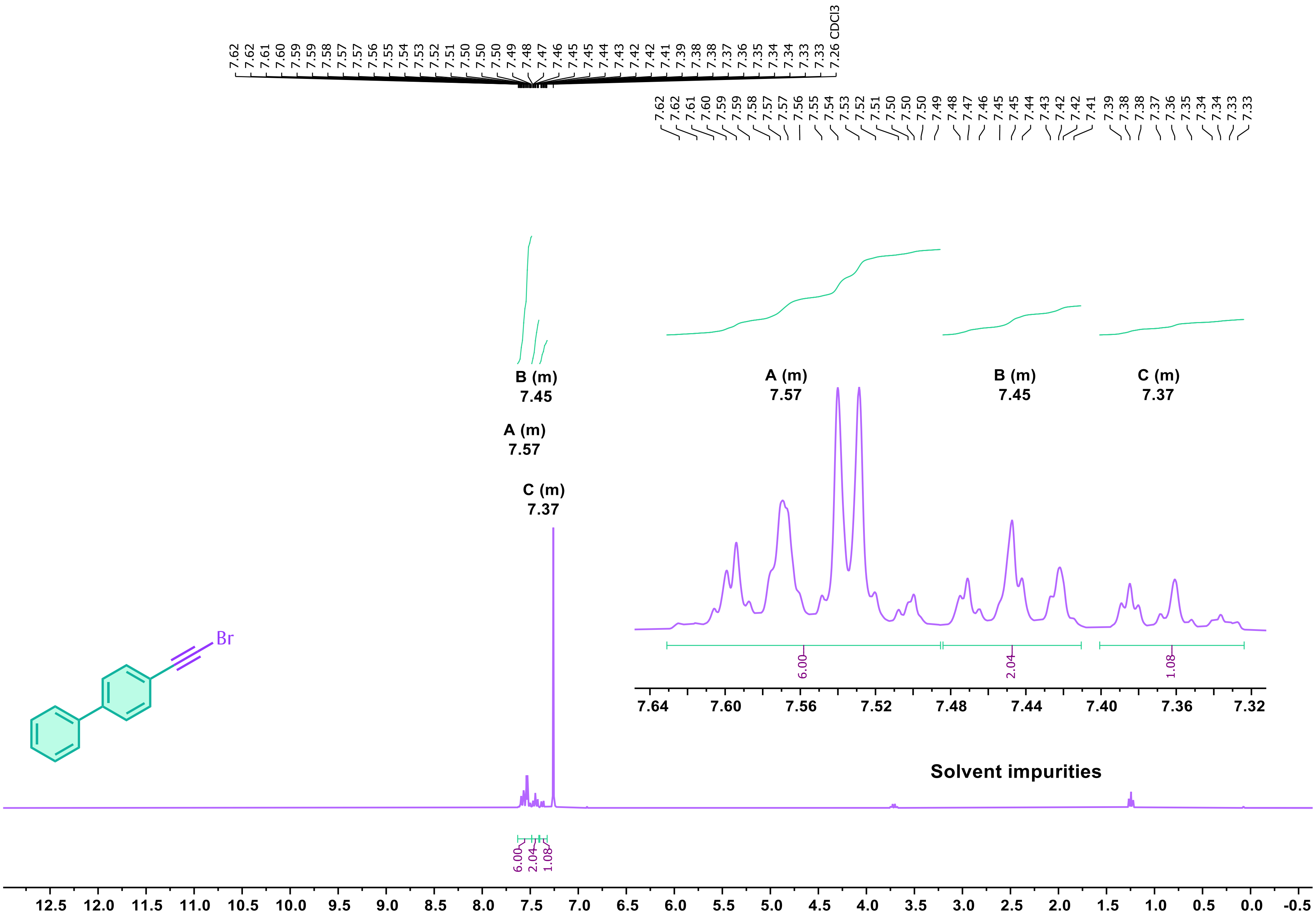

Figure S67. ^1H NMR spectrum (300 MHz, $CDCl_3$) of compound **6d**.

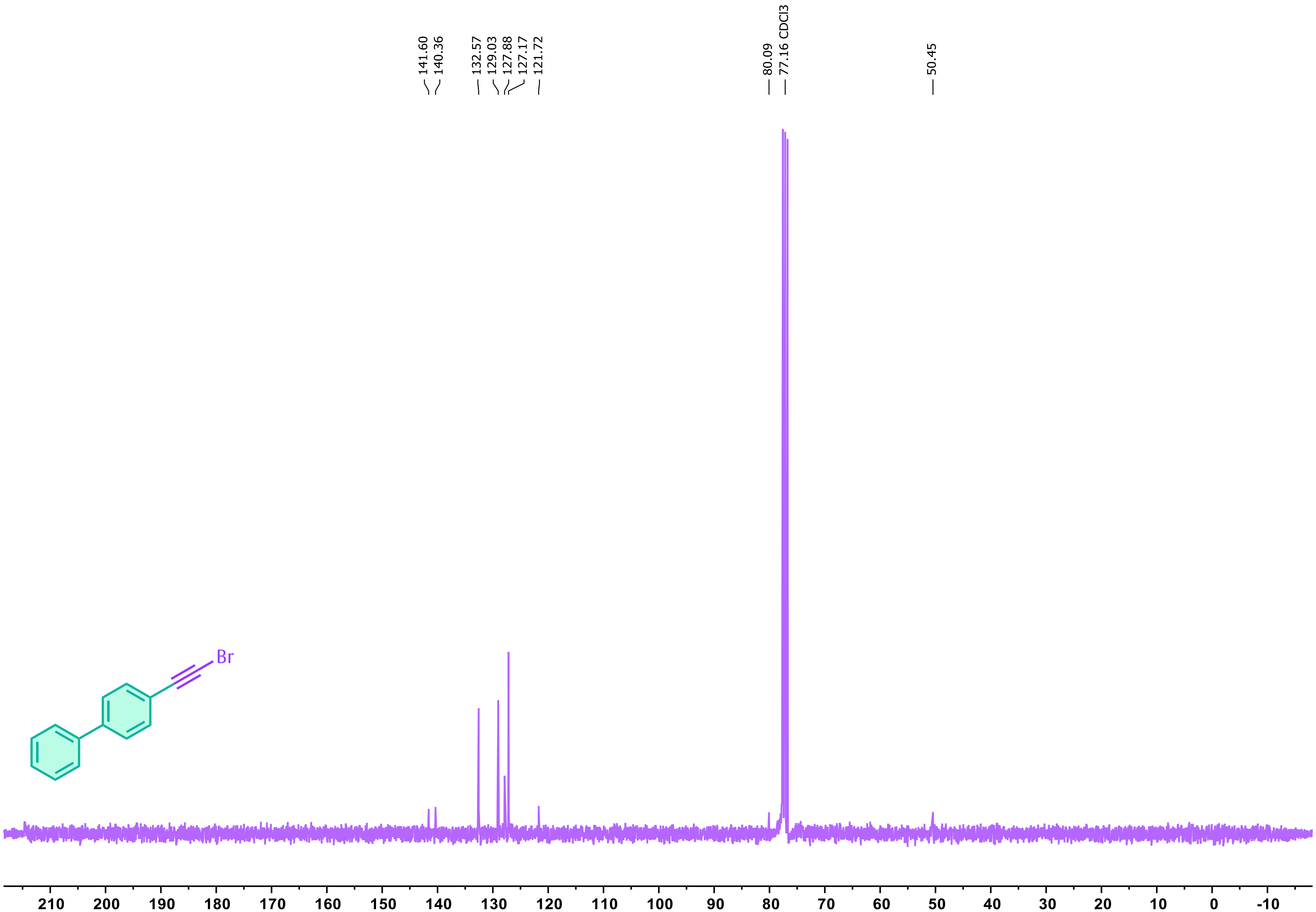

Figure S68. $^{13}C\{^{1}H\}$ NMR spectrum (75 MHz, $CDCl_3$) of compound **6d**.

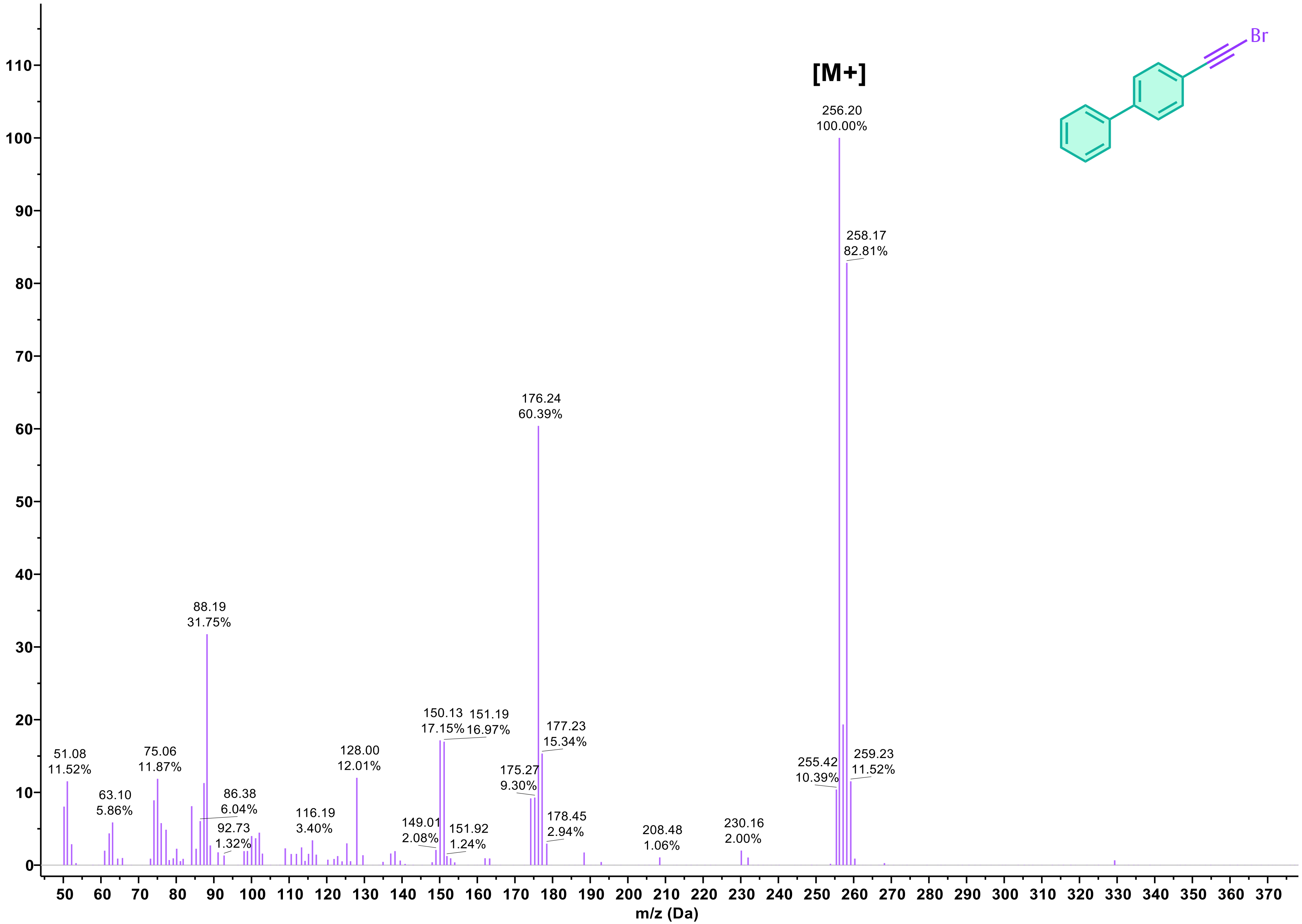

Figure S69. Mass spectrum of compound **6d**.

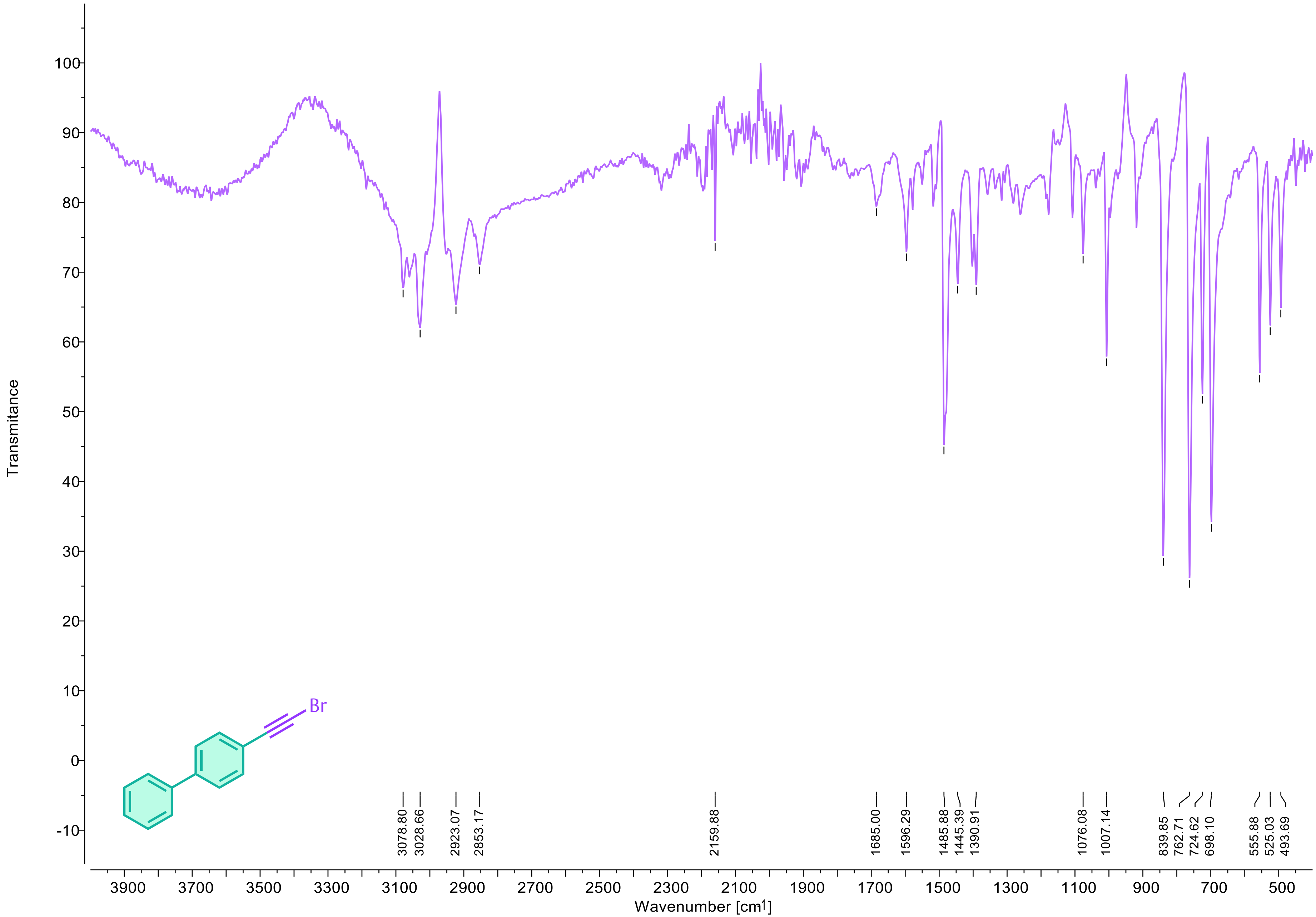

Figure S70. FT-IR spectrum of compound **6d**.

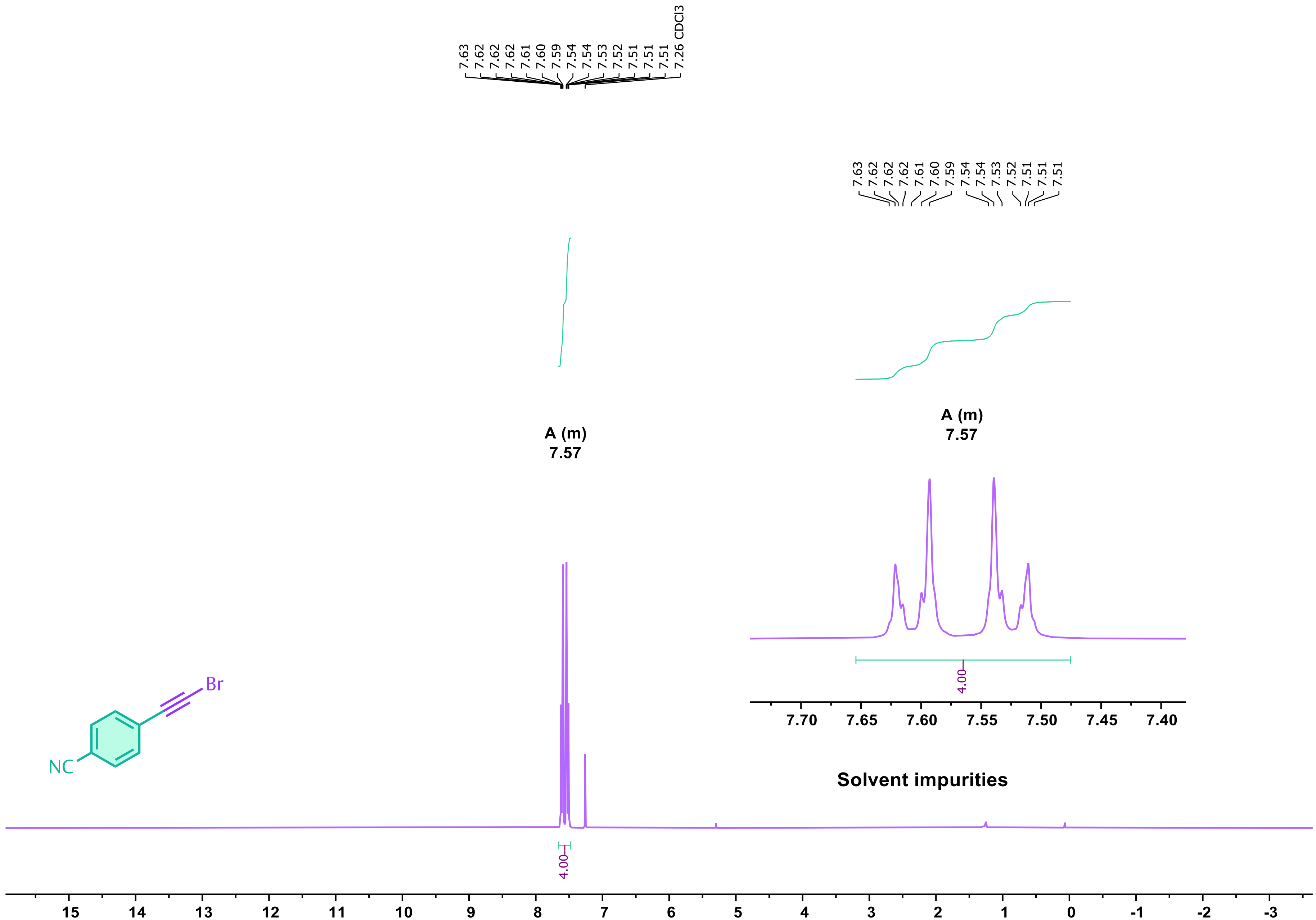

Figure S71. ^{1}H NMR spectrum (300 MHz, $CDCl_3$) of compound **6e**.

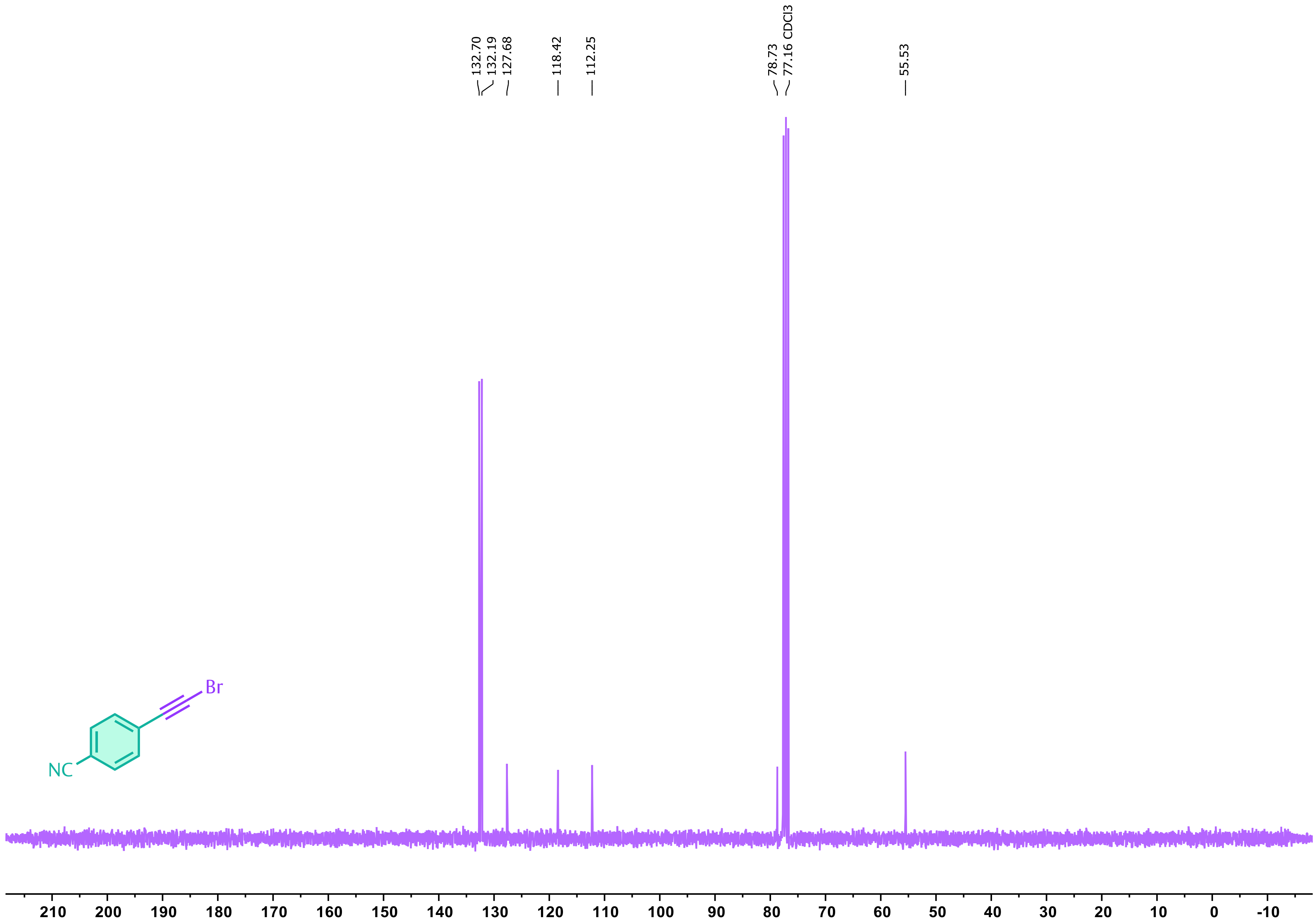

Figure S72. $^{13}C\{^1H\}$ NMR spectrum (75 MHz, $CDCl_3$) of compound **6e**.

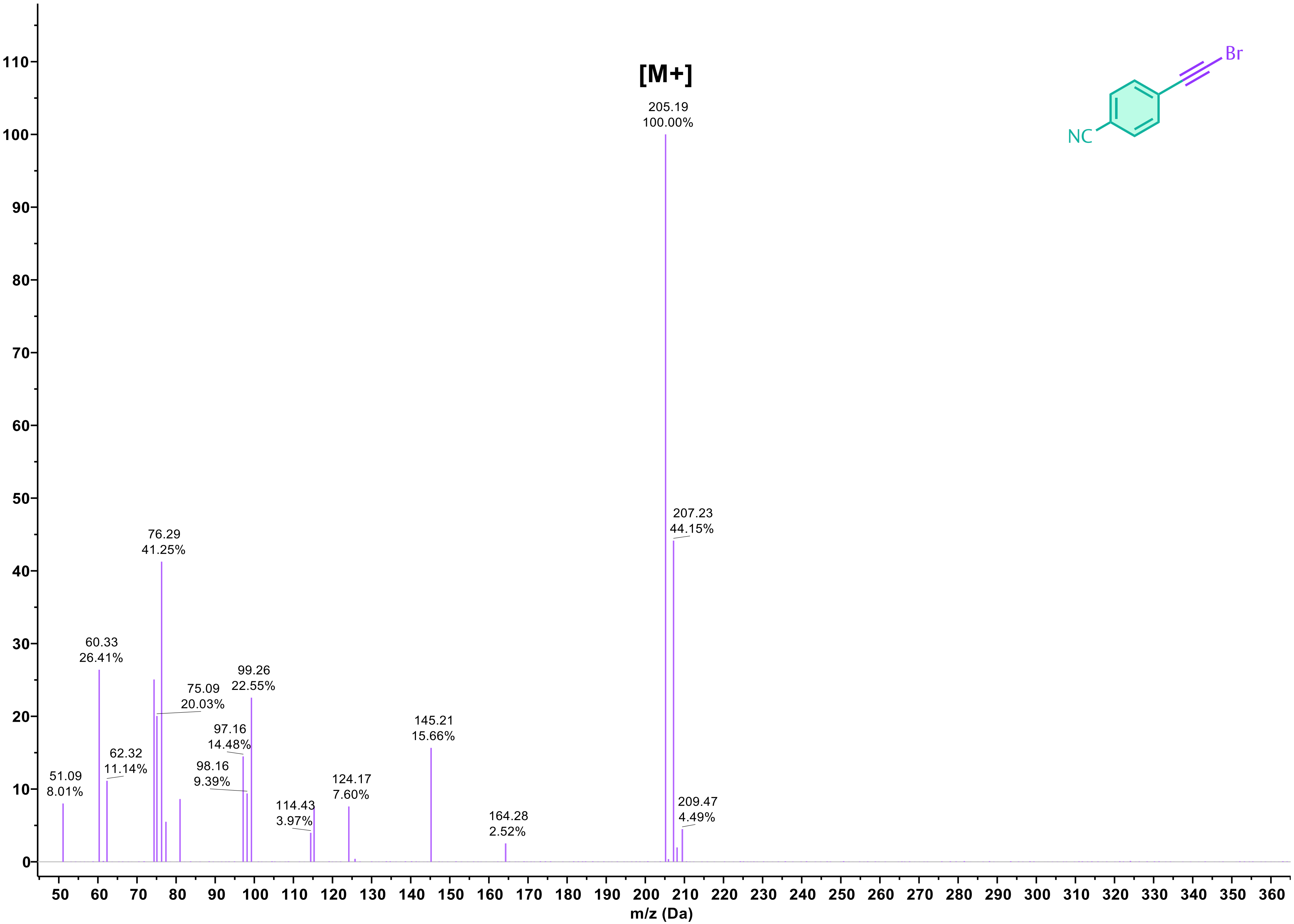

Figure S73. Mass spectrum of compound **6e**.

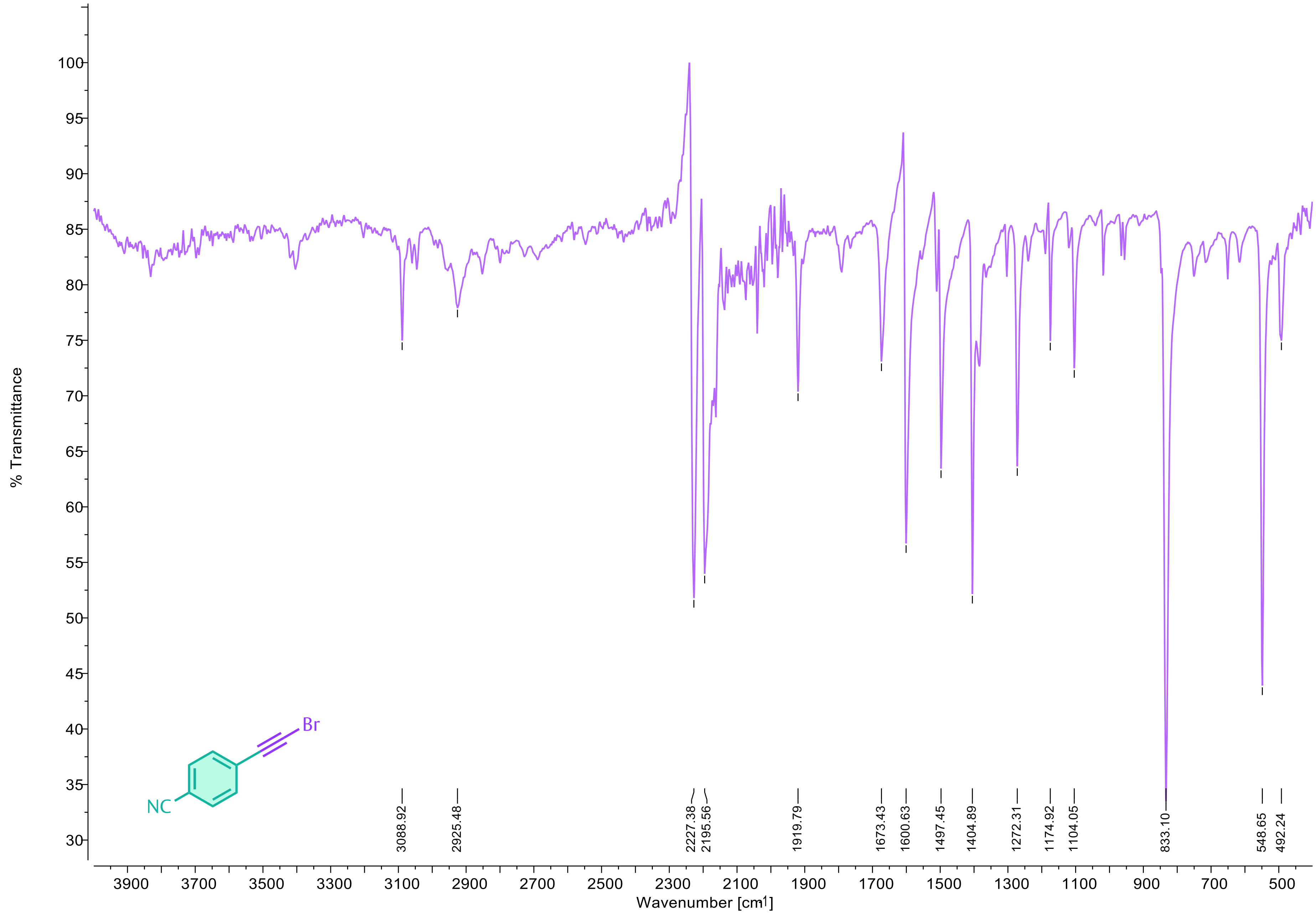

Figure S74. FT-IR spectrum of compound **6e**.

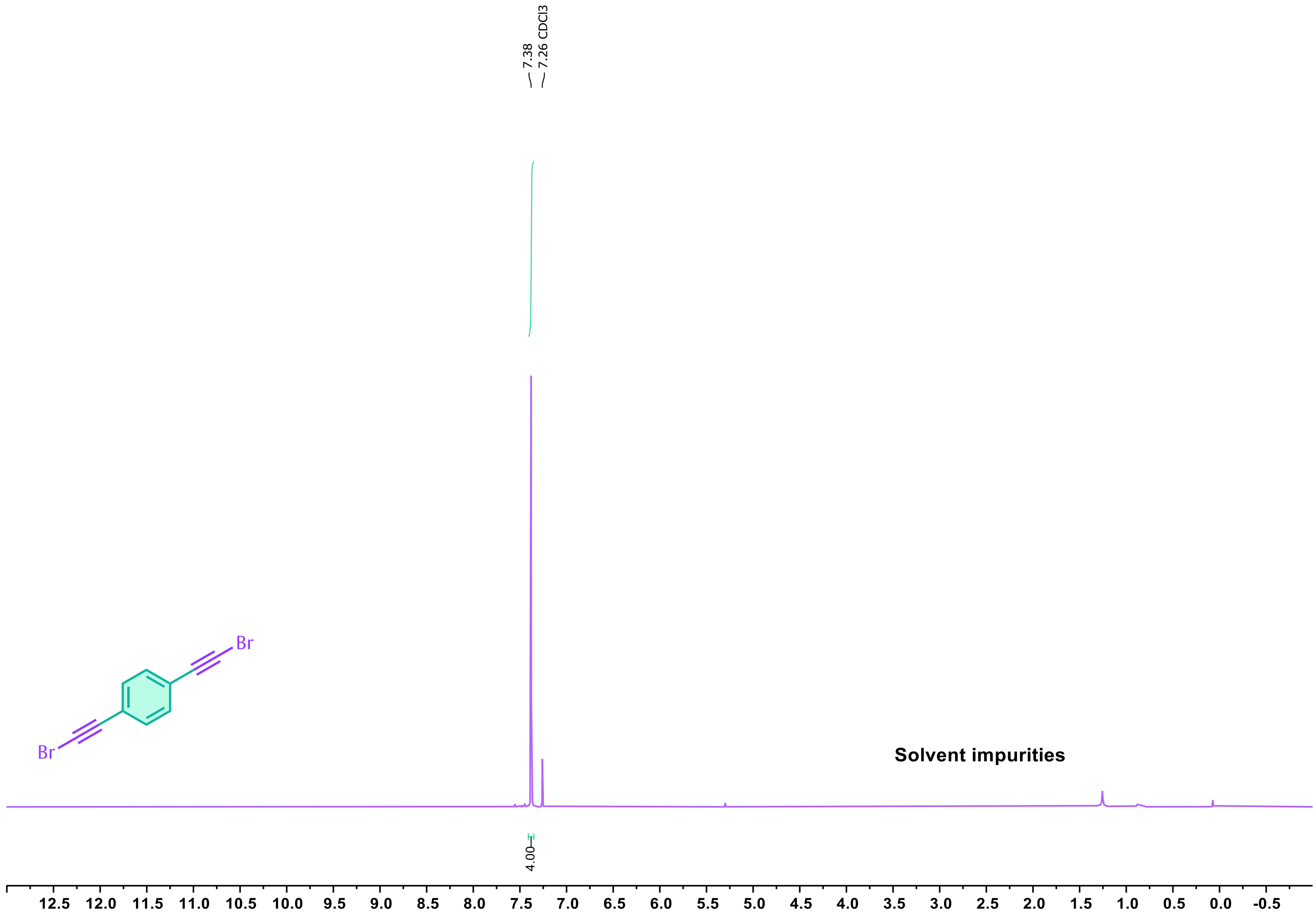

Figure S75. ^{1}H NMR spectrum (300 MHz, $CDCl_3$) of compound **6g**.

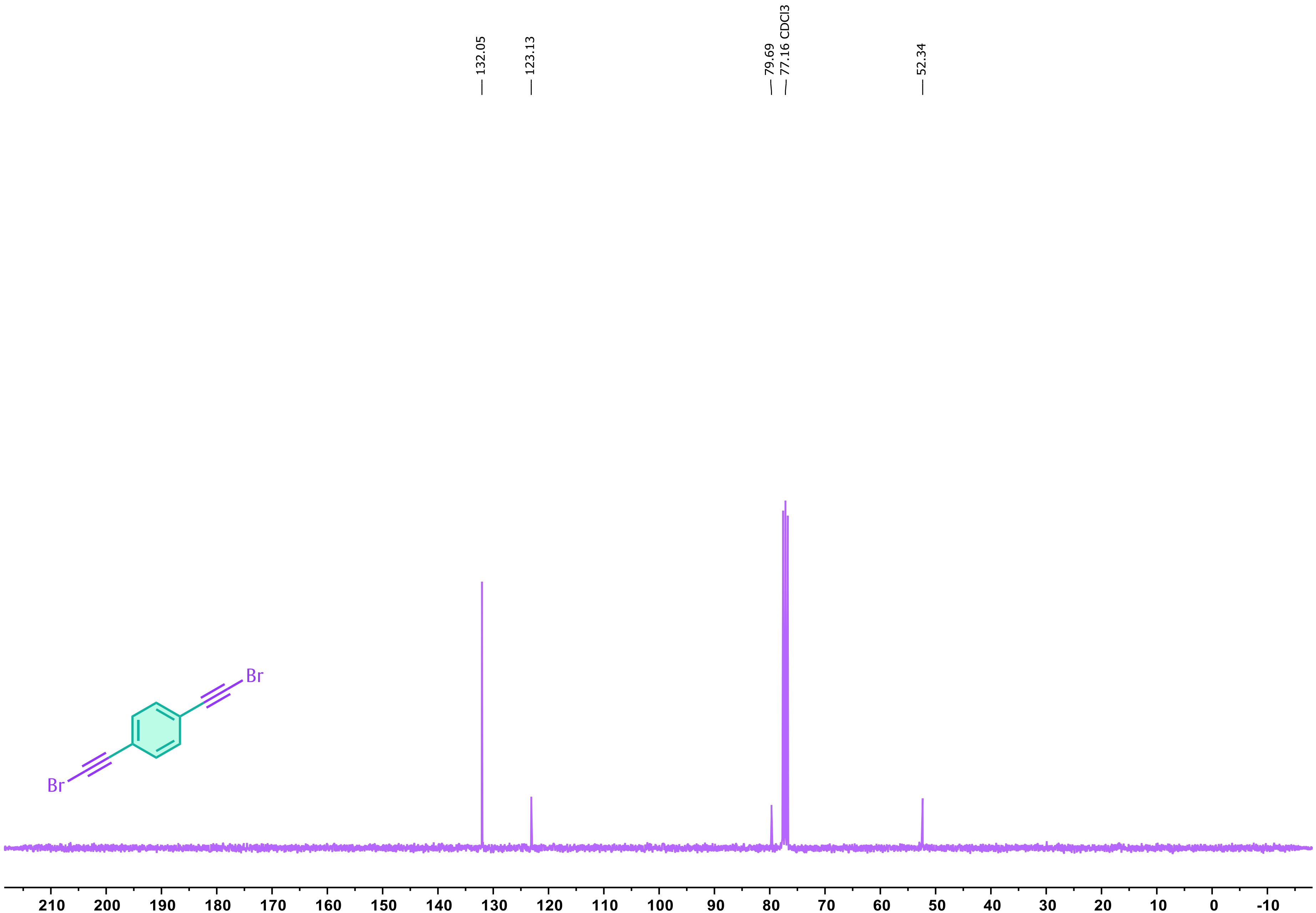

Figure S76. ^{13}C NMR spectrum (75 MHz, $CDCl_3$) of compound **6g**.

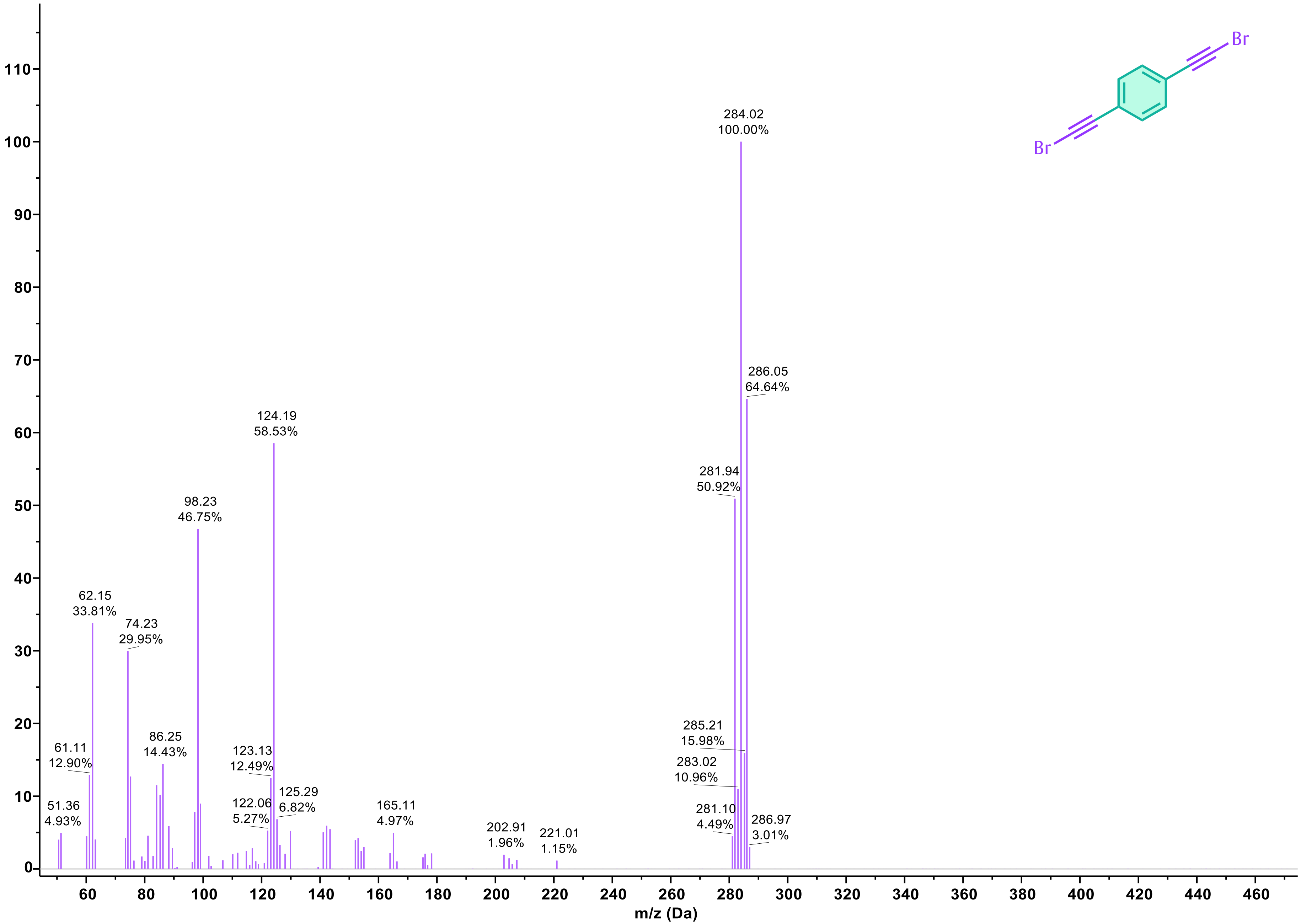

Figure S77. Mass spectrum of compound **6g**.

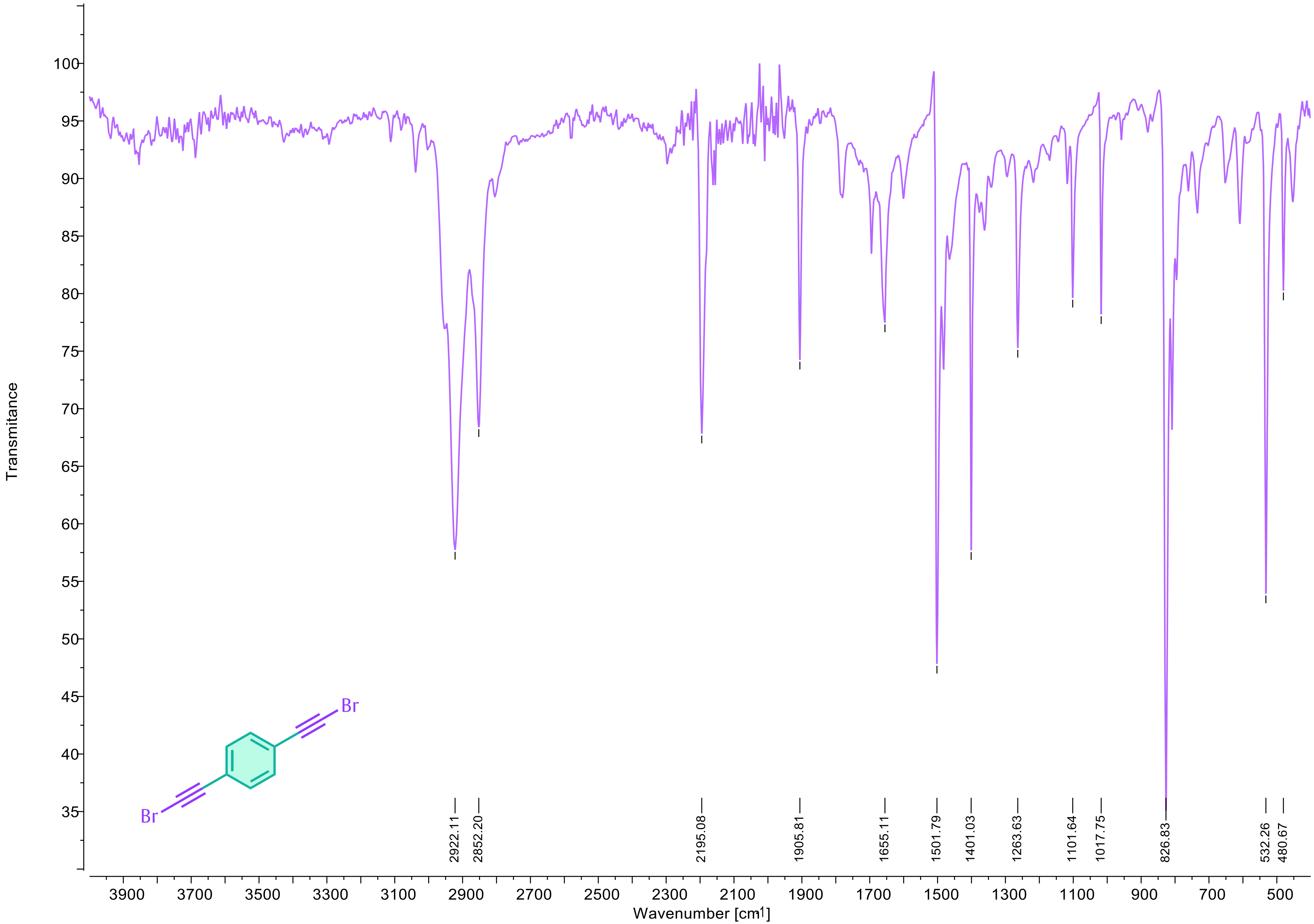

Figure S78. FT-IR spectrum of compound **6g**.

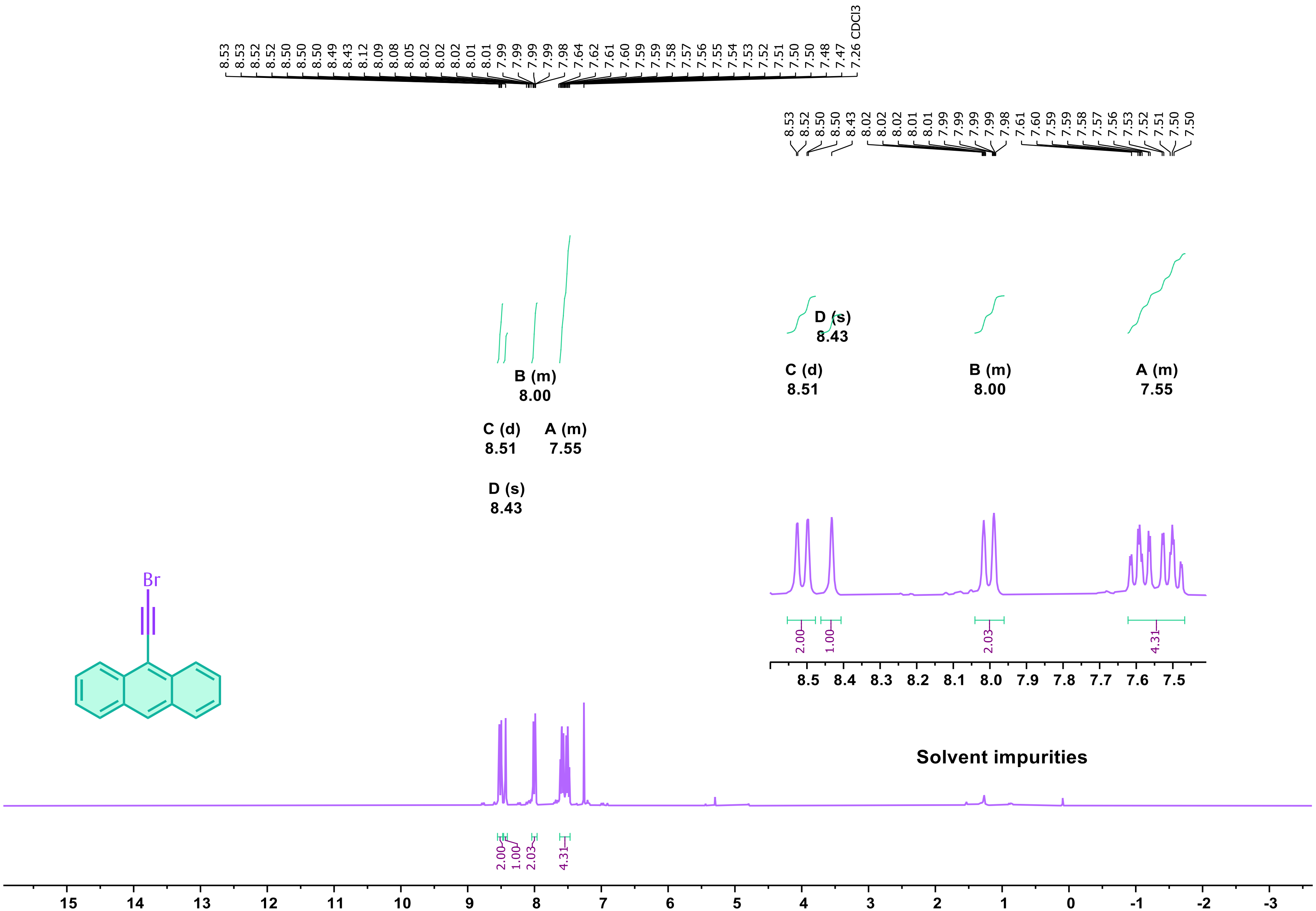

Figure S79. ^{1}H NMR spectrum (300 MHz, $CDCl_3$) of compound **6h**.

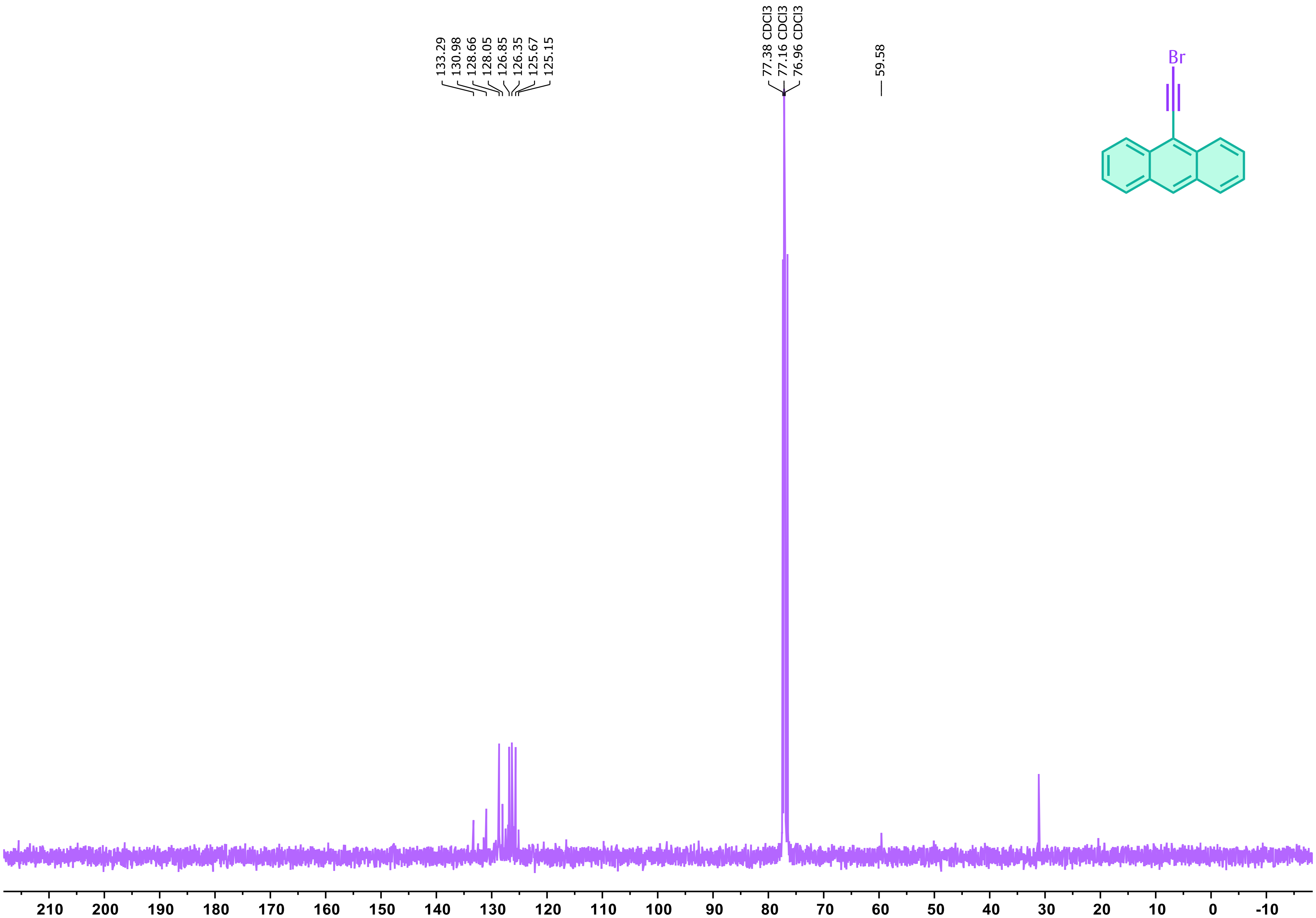

Figure S80. ^{13}C NMR spectrum (75 MHz, $CDCl_3$) of compound **6h**.

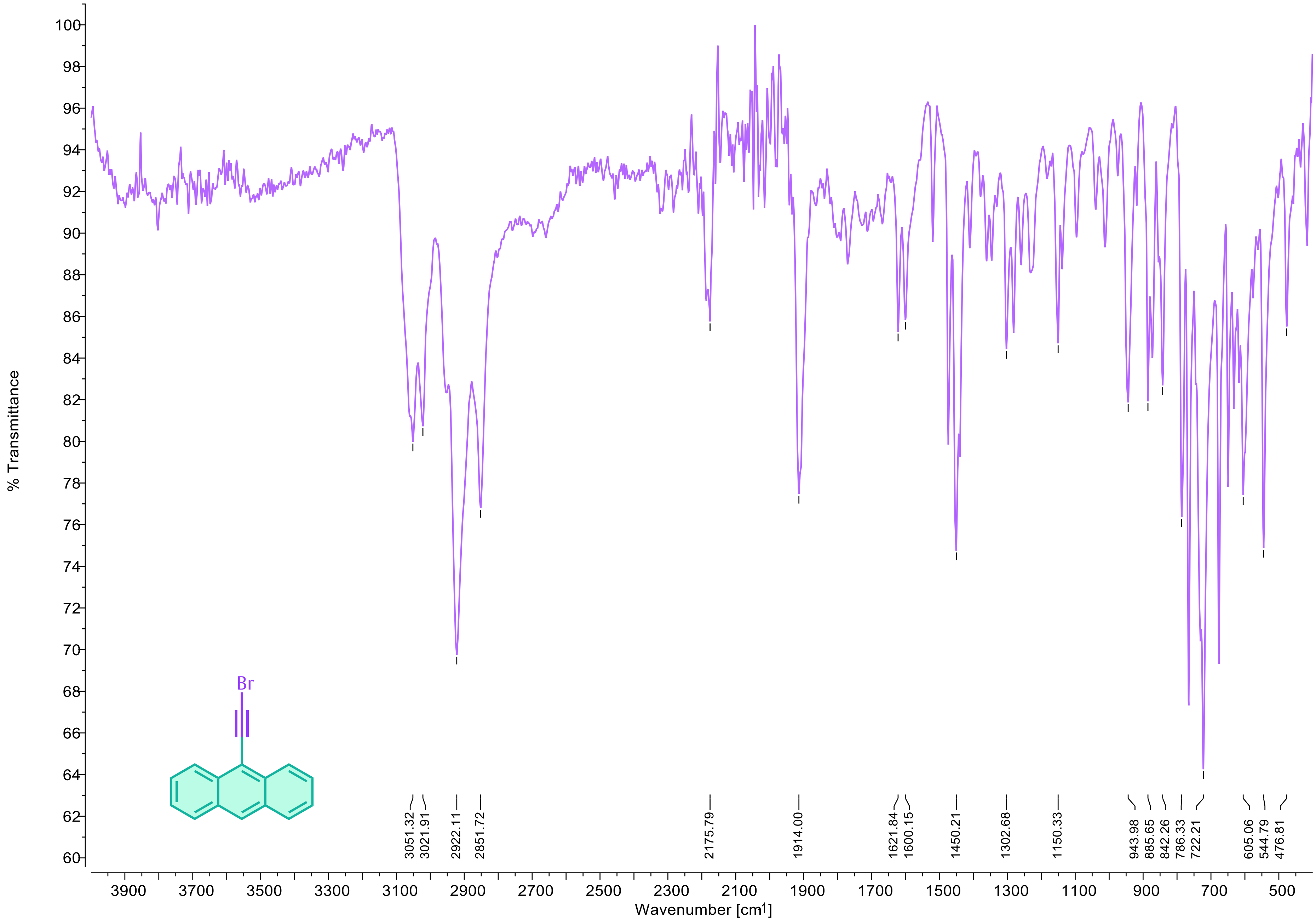

Figure S81. FT-IR spectrum of compound **6h**.
